# Exploring nursing assistants’ competencies in pressure injury prevention and management in nursing homes: a qualitative study using the iceberg model

**DOI:** 10.1186/s12912-025-02911-6

**Published:** 2025-03-27

**Authors:** Yanxia Guo, Wen Zhu, Plernpit Boonyamalik, Arpaporn Powwattana, Baolu Zhang, Junjun Sun

**Affiliations:** 1 School of Nursing and Midwifery, Jiangsu College of Nursing, Huai’an, 223005 China; 2 Department of Public Health Nursing, Faculty of Public Health, Mahidol University, Bangkok, 10400 Thailand; 3 Department of Neurology, Yancheng Third People’s Hospital, Yan Cheng, 224000 China; 4 School of Nursing, Southwest Medical University, Luzhou, 646000 China; 5 School of Nursing, Xinxiang Medical University, Xinxiang, 453003 China

**Keywords:** Pressure injury, Prevention and management, Nursing assistants, Competencies, Qualitative research

## Abstract

**Background:**

As the global population ages rapidly, pressure injuries (PI) in nursing homes have become a serious health concern. Nursing assistants play an important role in pressure injury prevention and management (PIPM).

**Objective:**

This study aimed to explore the PIPM competencies required by nursing assistants within nursing homes based on the Iceberg Model.

**Methods:**

We employed a qualitative descriptive study and collected data through a focus group with 12 nursing assistants and semi-structured interviews with 11 key stakeholders in China. Deductive content analysis was utilized based on the Iceberg Model.

**Results:**

Five main categories of nursing assistants’ PIPM competencies were identified: theoretical knowledge, comprehensive skills, self-concept, traits, and motives. Theoretical knowledge included PI basic and professional theoretical knowledge, emphasizing the importance of understanding PI mechanisms, risk factors, and prevention strategies. Comprehensive skills encompassed practical skills (e.g., repositioning techniques, wound care), communication skills, collaboration skills, and observation skills. Self-concept involves professional identity, ethical awareness, and a sense of responsibility. Traits included carefulness and love, and empathy and patience. Motives were driven by professional development opportunities and supportive institutional policies.

**Conclusion:**

The application of the Iceberg Model enhanced the understanding of competencies required for effective PIPM. The findings can guide the development of targeted training programs and assessment tools for nursing assistants in long-term care facilities. Future research should explore the long-term impacts of well-trained nursing assistants on patient outcomes and expand the PIPM competencies scope required by nursing assistants.

**Clinical trial number:**

Not applicable.

**Supplementary Information:**

The online version contains supplementary material available at 10.1186/s12912-025-02911-6.

## Introduction

The proportion of older persons aged over 65 years in China will reach 20% by 2050, which is estimated by the World Health Organization (WHO) as one of the most serious countries about the aging population issue [1]. With the Chinese population aging rapidly, the number of people with disabilities also increases. According to the “Long-term Care Survey Report 2018–2019”, among older persons over 60 years old, 4.8% are severely disabled in daily activities; 7% are moderately disabled, and the total disability rate is 11.8% [2]. The demands for professional long-term care (LTC) are increasing nowadays. The WHO referred to LTC as indispensable in the healthcare system and social services, which consists of a range of medical, personal care, and assistance services that are provided with the primary goal of alleviating pain and reducing or managing the deterioration in health status for people with a degree of long-term dependency [3]. Nursing homes are the main LTC facilities, and nursing home residents are susceptible to pressure injury (PI), due to skin aging, limited mobility, and sensory impairment [4]. PI is also referred to as bedsores, decubitus, pressure ulcers, and pressure sores. It is described as localized damage caused by pressure or pressure combined with shear to the skin and/or underlying tissue [5]. PI is one of the most frequently occurring and costly, yet preventable adverse events in institutions and is of particular concern for elderly, which is a well-recognized complication of nursing homes [4]. PI could trigger a tremendous burden not only on individuals but also on the healthcare systems [4]. The significance of pressure injury prevention and management (PIPM) in nursing homes is widely recognized by consumers and stakeholders, assuming that the incidence and prevalence of PI are indicators of poor nursing care quality [4]. Studies showed that the PI prevalence in nursing homes varied globally; in Ireland, the prevalence of PI in nursing homes was reported 9% [6]; in Japan, 5.8% [7]; in Italy, 11.6% [8]; and in China, 17.2% [9]. The PI incidence in nursing homes varied from various countries. In United States, it was reported 3.6% [10]; in Brazil, 39.4% [11]; in Netherlands, 33.3% [12]; and in China, 28.9% [9].

Nursing assistants are essential members of the frontline team who devote a lot of time observing and tracking the outcomes of LTC residents, and they make up the majority of the workforce and caregivers in nursing homes [13]. In addition, they provide 90% of nursing care services and frequently identify the first signs and symptoms of PI in LTC settings [13]. Therefore, nursing assistants assume significant roles and responsibilities in PIPM. For example, making sure residents are in a comfortable position; monitoring skin color, texture, and abnormalities and reporting findings to nurses; making sure that residents are eating healthy, documenting intake and reporting to nurses, and assisting patients to walk; implementing a plan of care with the supervision of registered nurses; initiating use of pressure-relieving devices; turning and repositioning patients and documenting, performing moisture/skin care, notifying nurses of skin changes, and so on [14, 15]. Consequently, competent nursing assistants with good attitudes, knowledge, and skills are essential to provide high-quality nursing services [16]. The Chinese Health Commission launched a notice on strengthening the training and standardized management of nursing assistants in 2019, which mentioned that institutions should use trained and qualified nursing assistants to engage in corresponding work, employ legal and standardized labor, and require active training based on the training outline [17]. However, there are no related studies probing the PIPM competencies requirements for nursing assistants in nursing homes globally, which makes it hard for nursing homes to establish and implement training interventions effectively. Most research mainly focused on nurses’ competencies or nursing assistants’ competencies in other areas. Competency refers to the capacity to perform a job effectively, combining knowledge, skills, attitudes, and values that contribute to efficient and excellent performance in a professional or occupational domain [18, 19]. The International Council of Nurses identified the competencies of generalist nurses under three domains: (1) professional, ethical, and legal practice, (2) care provision and management, (3) professional, personal, and quality development [20]. Several studies and guidelines have identified specific competencies required for effective PIPM. For instance, Peršolja et al. [21] emphasized healthcare employees are knowledgeable about the development, classification, and characteristics of PI, the knowledge of preventive measures, tools, prevention, and modern PI care. Aquino et al. [22] highlighted the capabilities of assessment and differentiation of PI from other wounds and prevention interventions for nursing staff. Understanding the competencies required for PIPM is not only crucial for current practice but also for anticipating future changes in healthcare delivery. As healthcare practices evolve, nursing assistants’ capacities will need to adapt to new technologies and methodologies, such as the use of electronic health records, telehealth technologies, and advancements in wound care technology [23, 24]. Therefore, it is necessary to identify nursing assistants’ PIPM capabilities deeply from nursing assistants’ and key stakeholders’ perspectives. Competency models have been applied to job analysis, performance evaluation, and competency training [25]. The Iceberg Model is a widely recognized competency theory, where knowledge and skills represent the surface characteristics of the “iceberg,” that are easily observed and measured. In contrast, factors such as traits, self-concept, and motives are identified as hidden characteristics, making them more difficult to assess [26, 27]. The Iceberg Model can offer a thorough viewpoint on exploring capacities and competency-based nursing education [26].

Nursing assistants’ PIPM competencies in nursing homes have not been extensively studied so far. To deepen our understanding of this knowledge, we carried out a qualitative study based on the Iceberg Model. This approach offers valuable clinical reference and a practical framework for assessing and training nursing assistants’ abilities in PIPM. Understanding these competencies will not only enhance current care practices but also prepare nursing assistants for future advancements in healthcare delivery and technology integration.

## Methods

### Design

We employed a qualitative descriptive design to conduct a focus group with nursing assistants and semi-structured interviews with key stakeholders. This design was specifically chosen to provide a straightforward and detailed description of the phenomenon under study [28].

### Participants

We used the purposive sampling method to recruit participants from Jiangsu Province of China at some institutions including nursing homes, community healthcare centers, hospitals, university institutions, and local government. The institutions were selected based on the contexts, ensuring accessibility and feasibility within the scope of the study. We collaborated with local healthcare networks and administrative contacts to identify and approach suitable institutions. Nursing assistants recruited for the focus group were those who work in nursing homes with at least 3 months of working experience and had the experience of performing PIPM for residents. The inclusion criteria of key stakeholders in the in-depth interviews were:1) nurses who are from hospitals, community healthcare centers, and nursing homes; at least 5 years of working experience; undergo the experience of providing PI training for nursing assistants. 2) nursing teachers who had at least 5 years of working experience and held associate senior profession titles with the PI field research interest. 3) nursing homes’ administrators and the policy-maker of local government. The exclusion criterion was unwilling to participate in the study for any reason and was not able to speak and understand Chinese Mandarin.

The researchers recruited potential participants through telephone, email, and WeChat, utilizing professional networks and collaborations with local healthcare institutions in Jiangsu Province. Initial contact was made by sending detailed information about the study’s purpose, procedures, and the expected time commitment. After expressing interest, participants were invited to review the consent form and were asked to confirm their willingness to participate in the focus group (for nursing assistants) or individual interviews (for key stakeholders). A total of 18 nursing assistants were initially approached to participate in the focus group, and of these, 12 agreed to participate. A total of 14 key stakeholders were invited for in-depth interviews, and of these, 11 agreed to participate.

### Data collection

One focus group comprising 12 nursing assistants and in-depth interviews comprising 11 key stakeholders involved in data collection and gathered their perspectives on PIPM competency. In July 2024, after obtaining participants’ permission, researchers made an appointment with them to choose the interview sites and time at their convenience via telephone or WeChat app. Before the interview, they were asked to sign the informed consent form and to provide information about their background. With the interviewees’ consent, the audio recording was used for the whole interview process. The interview questions were triggered by the Iceberg Model [26, 27]. The questions for the focus group are: (1) what PIPM knowledge and skills do nursing assistants need to care for the elderly? (2) what traits must nursing assistants have to perform PIPM for the elderly? (3) how important do you think PI prevention is in your role as a nursing assistant? (4) how do you view the impact of your work on residents’ overall health and quality of life, especially regarding PI? (5) what motivates you to prioritize PI prevention in your daily work? (6) what challenges do you face when performing PIPM for the elderly? (7) what resources or support do you feel are necessary for effective PIPM? The questions for the in-depth interviews are: (1) what specific competencies do you observe in nursing assistants that contribute most to effective PIPM? (2) what is your perspective on the importance of nursing assistants’ attitudes or values toward PI prevention? (3) what personality traits do you think drive nursing assistants to be proactive in PIPM? (4) how do institutional culture and policies influence nursing assistants’ motivation to perform PIPM? (5) what motives would further empower nursing assistants to perform PIPM effectively? Participants were encouraged to express themselves openly. The moderator ensured that the discussion remained focused on the actual topic and also posed follow-up questions, periodically verifying that the interviewer had accurately understood the informant’s meaning. Each interview lasted 30 ~ 60 min. The observation and field notes techniques were used to record the main situation and meaningful content to access more information.

### Data analysis

To systematically analyze the data collected from nursing assistants and key stakeholders, we employed deductive content analysis based on the Iceberg Model using the MAXQDA software. All audio recordings were transcribed verbatim. The transcripts were reviewed multiple times to gain a comprehensive understanding of the data. The analysis proceeded in two phases. Initially, the transcripts were subjected to concept-driven coding, which is a deductive approach for developing a coding framework/categorization matrix based on existing theoretical foundations, previous studies, or logical frameworks [29, 30]. In this study, the Iceberg Model served as the theoretical framework for the concept-driven analysis. The texts were categorized according to the Iceberg Model’s five dimensions of theoretical knowledge, comprehensive skills, self-concept, traits, and motives [26, 27]. In the second phase, after categorizing the content according to the five dimensions of the Iceberg Model, each category underwent independent qualitative content analysis, as outlined by Graneheim and Lundman [31]. Meaning units were identified, sentence content was condensed and coded. Following team reflection and discussion, these codes were organized into main categories and subcategories.

### Trustworthiness

To guarantee the trustworthiness with high credibility, transferability, dependability, and confirmability of the qualitative study, the triangulation methods were employed. Triangulation is the process of using several techniques or data sources to create a thorough understanding of a phenomenon [32]. We applied interviews, observation, and field notes to achieve method triangulation. The Investigator triangulation involved two or more researchers in the study to provide multiple observations and conclusions to achieve accurate data and diverse perspectives [32]. Each researcher independently coded and analyzed the transcripts, and discrepancies were discussed and resolved through consensus. We conducted regular team meetings where researchers shared their interpretations and insights to minimize individual biases and ensured that the findings were rigorous and reliable. Data source triangulation involved data collection from diverse participants, including nursing assistants, nurses, nursing teachers, administrators, and policymaker to obtain multiple perspectives. To minimize potential biases in participants’ responses, we ensured that the interview environment was comfortable and non-threatening, and the interview questions were open-ended and non-leading, applying probing questions to encourage participants to share their honest opinions and experiences, emphasizing that there were no right or wrong answers. To enhance the validity of our findings, we employed member checking to provide participants with the preliminary findings to verify their accuracy and completeness.

### Ethical considerations

This research was approved by the ethical review board at Mahidol University in Thailand (IRB number: MUPH 2024-066) and Jiangsu College of Nursing in China (IRB number: JSCN-ME-2024071801). We got each participant’s written informed consent before the study. Participant privacy and confidentiality were protected throughout.

## Results

A total of 12 nursing assistants and 11 key stakeholders were recruited in this study. Among nursing assistants, 91.7% were female, and they were aged 38 to 51 years (mean age 46±3.838 years). Seven nursing assistants (58.3%) had a junior high school degree, and others (41.7%) had a senior high school degree. 66.7% of nursing assistants had working experience of more than 5 years. 58.3% of them had the experience of taking care of patients with PI and 83.3% of them received PI training before. Besides, 66.7% of them got the senior professional level certificates, while others obtained the intermediate level.

Among the key stakeholders, one was the nursing home’s registered nurse, four were hospital registered nurses (3 wound care specialists, and 1 geriatric care specialist), one was a community registered nurse, two were nursing homes’ administrators, two were nursing teachers, and one was a policy-maker of local government. 81.82% of them were female and they were aged 33 to 50 years (mean age 39.82±6.447 years). Two of them (63.6%) had a bachelor’s degree, three (27.3%) had a master’s degree, and one (9.1%) had a doctoral degree. Six of them (54.5%) had an intermediate profession title, three (27.3%) had an associate senior profession title, and two (18.2%) had a senior profession title. The range of working experience and experience with taking care of PI patients varied from 8 to 26 years (mean 15.36±6.918 years) and from 4 to 15 years (mean 8.36±3.557 years), respectively.

**Fig. 1:**
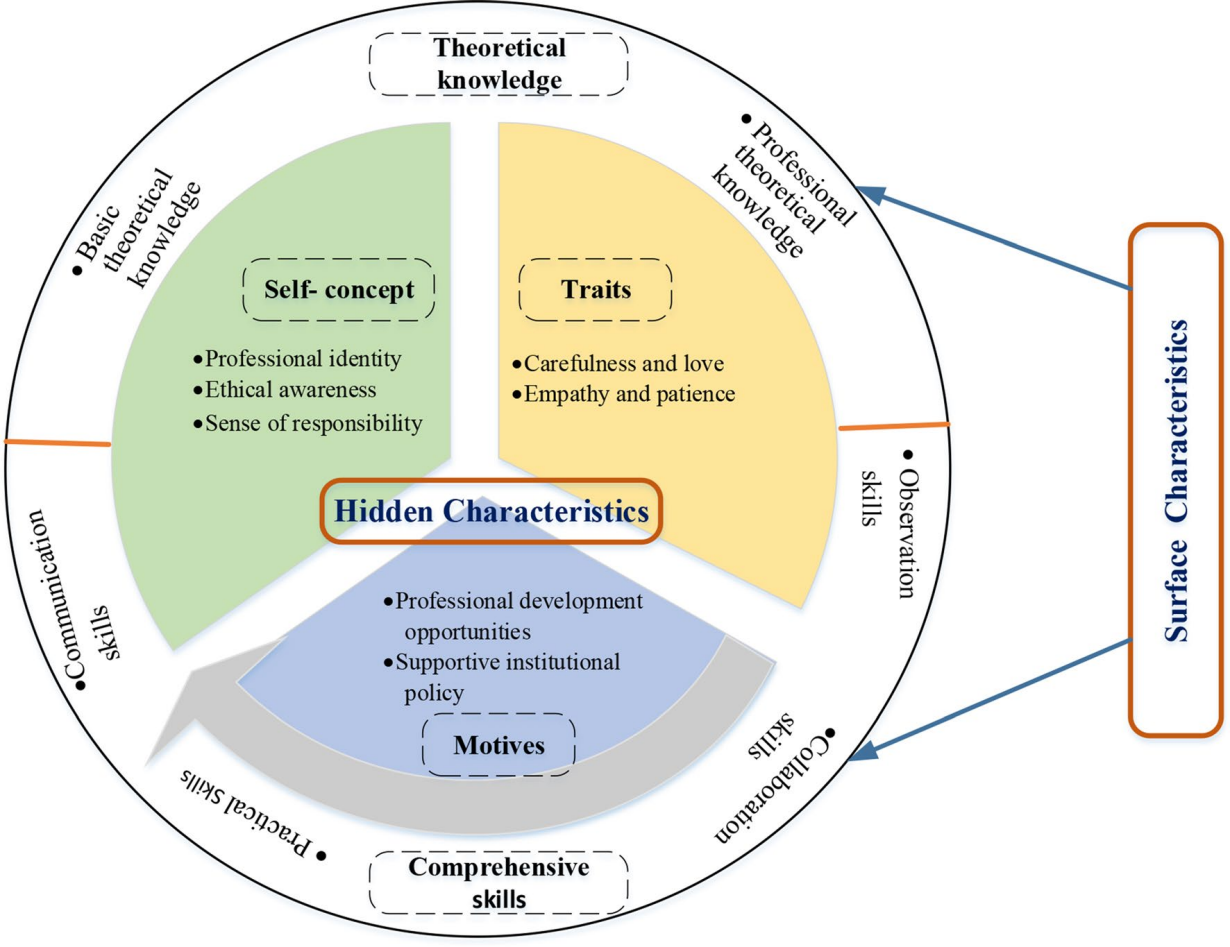
The Iceberg Model of nursing assistants’ PIPM competencies

Nursing assistants’ PIPM competencies based on Iceberg Model analysis were recognized into five main categories encompassing 13 subcategories and 47 codes (Table 1). The specific identified meaning units are attached in the Supplementary Material. The Iceberg Model of nursing assistants’ PIPM competencies is shown in Fig. 1.

**Table 1:** Main categories, subcategories, and codes forming nursing assistants’ PIPM competencies

Main Category	Subcategory	Codes
Theoretical knowledge	Basic theoretical knowledge	Professional ethics and code of conduct for nursing assistants
		Legal and ethical issues related to elderly care and PI
		Definition and epidemiological characteristics of PI
		The hazards and dangers of PI
		Mechanisms and risk factors of PI
		Staging and clinical manifestations of PI
		High-risk populations and body sites for PI
	Professional theoretical knowledge	Causes and characteristics of PI among the elderly
		Methods for risk assessment of PI
		Methods for differentiating PI from other common skin problems
		Nutrition care
		Skin care
		Treatment and management of PI
		Types and functions of pressure relief equipment
		Types and characteristics of dressings
		Psychological care for patients with PI
Comprehensive skills	Practical Skills	Tuning over and repositioning skills
		Position transfer techniques
		Wound dressing change technique
		Use of walking aids
		Selection and usage methods of dressings
	Communication skills	Communication with nurses
		Communication with the elderly and families
	Collaboration skills	Collaboration with nurses
		Collaboration with other nursing assistants
	Observation skills	Observing the skin condition
		Observing the elderly’s physical and mental condition
Self-concept	Professional identity	Enthusiasm for the elderly care profession
		Sense of belonging
		Commitment to work
	Ethical awareness	Comply with the laws and regulations
		Respect the elderly’s esteem and privacy
		Treat the elderly equally
	Sense of responsibility	Pay attention to PI prevention
		Emphasis on care quality
		Don’t shirk responsibility
Traits	Carefulness and love	Checking the elderly’s skin carefully
		Encourage the elderly to be self-reliant within their capabilities
		Do not blame the elderly
		Actively focusing on the needs of the elderly
	Empathy and patience	Ability to stand in elderly’ points
		Be patient with the elderly’s slow reaction
Motives	Professional development opportunities	Competency enhancement
		Job Promotion
	Supportive institutional policies	Assessment and evaluation mechanism
		Continuing education credits
		Reward mechanism

### Theoretical knowledge

Theoretical knowledge refers to the foundational understanding and awareness of concepts, principles, and facts related to PIPM. This includes PIPM basic and professional theoretical knowledge necessary for effective care in this study.

### Basic theoretical knowledge

Basic theoretical knowledge encompasses fundamental concepts such as PI definitions, professional ethics, legal and ethical issues, mechanisms, stages, risk factors, and high-risk populations and body sites. As some nurses said: *“Nursing assistants need to know what PI is and why it is an important indicator of quality control in nursing home management”; “Understanding the different stages of PI can help accurately assess the patient’s wound condition. The staging of PI is crucial for determining treatment options (such as dressing selection*,* turning frequency*,* etc.”*

### Professional theoretical knowledge

Professional theoretical knowledge includes specialized information related to the assessment, prevention, and management of PI. For example, participants mentioned the causes and characteristics of PI among the elderly, risk assessment methods, differentiating PI from other skin problems, nutrition care, skin care, treatment and management of PI, pressure relief equipment and dressings, and psychological care for patients with PI. As one nursing assistant said: *“Sometimes it is difficult for us to identify whether it is a PI skin problem during nursing care*,* and we are not sure to make a correct judgment. We don’t know whether it is a PI*,* incontinence-related dermatitis*,* or other skin problems*,* we can’t differentiate them correctly. Therefore*,* we often cannot implement preventive measures in time.”* Some nursing assistants said: *“We need to realize that PI is not only a physical condition*,* but also has an impact on the elderly’s psychological state. Elderly people with PI may feel depressed*,* anxious*,* or lose self-esteem. We need to learn to provide emotional support and encouragement.”*

### Comprehensive skills

Comprehensive skills refer to the practical abilities and competencies required to perform PIPM tasks effectively. These skills are observable and measurable. The following subcategories: practical skills, communication skills, collaboration skills, and observation skills were identified in comprehensive skills.

### Practical skills

Practical skills involve the physical techniques and procedures necessary for PIPM, such as tuning over and repositioning skills, position transfer techniques, wound dressing change technique, use of walking aids, and selection and usage methods of dressings were considered to be essential components of skills-related competencies. As one nurse said: *“Turning and changing body positions are basic techniques for preventing PI. Correctly turning techniques can help promote blood circulation in patients and reduce poor blood circulation caused by staying in the same position for a long time.”* Another nurse said: *“It is essential for nursing assistants to master basic wound dressing techniques to provide high-quality care*,* which helps ensure that patients’ wounds are properly managed.”*

### Communication skills

Communication skills involve the ability to convey information effectively with patients, families, and other healthcare providers. It was deemed by all informants to be a critical component of skills-related competencies. As one nurse mentioned: *“Effective communication between nursing assistants and nurses is the basis of teamwork. Nursing assistants are often the first to discover problems in daily patient care. They need to communicate with nurses in a timely manner about any changes in the patient’s skin condition*,* including risk factors for PI”.* Some nursing assistants said: *“Sometimes we encounter challenges in our work*,* such as it is difficult to communicate with some patients and their families. For example*,* some patients are not very cooperative in turning over*,* changing clothes*,* wiping their bodies to keep their skin clean*,* etc. We want to learn how to communicate effectively.”*

### Collaboration skills

Collaboration skills involve working effectively with other healthcare team members to provide coordinated care. For example, some informants highlighted the need for collaboration skills with nurses and other nursing assistants when conducting PIPM for the elderly effectively in nursing homes. One nurse said: *“The prevention and management of PI requires close collaboration between nurses and nursing assistants. The care plan developed by the nurses needs to be accurately implemented by the nursing assistants. This includes turning plans*,* skin care*,* nutritional support*,* and other PI prevention measures.”* Some assistants mentioned: *“Collaboration ability is required. PIPM is a team effort. We need to support each other*,* share responsibilities*,* and ensure that every nursing measure is properly implemented. By working together*,* we can share each other’s experiences and best practices and improve the level of care together.”*

### Observation skills

Observation skills involve the ability to notice and interpret changes in a patient’s condition, particularly related to skin integrity. For example, most participants emphasized that observing the skin condition and the elderly’s physical and mental condition is especially required for nursing assistants. As one nurse said: *“Nursing assistants’ ability to observe skin conditions is crucial for early identification of PI. Early detection of skin changes allows for preventive measures to be taken in advance. By observing skin color*,* temperature*,* moisture*,* and integrity*,* nurses can help us make accurate PI risk assessments.”* Another nurse said: *“By observing the patient’s behavior*,* expression*,* and speech*,* nursing assistants can detect possible psychological problems of the patient at an early stage*,* such as anxiety and depression*,* which may affect the patient’s physical health and wound healing*,* that ability is very important.”*

### Self-concept

Self-concept refers to the personal and professional identity of nursing assistants, including their attitudes and values towards their work in PIPM. For example, most nursing assistants and key stakeholders mentioned that nursing assistants should have a strong professional identity involving enthusiasm for the elderly care profession, a sense of belonging, and commitment to work. One nurse said: *“Identity acknowledge and passion for the elderly care profession is the driving force for nursing assistants to provide quality care services.”.* Some nursing assistants mentioned, *“We often take care of patients without fear of hardship*,* taking care of more than 5 elderly people every day*,* working more than 8 hours a day. This is our job. we must be prepared and committed to devote our time*,* energy and physical strength”.*

Ethical awareness was also emphasized by participants, which included complying with the ethical laws and regulations, respecting the elderly’s esteem and privacy, and treating the elderly equally. Nurses said: *“Many ethical laws and regulations set standards and requirements for nursing services. Nursing assistants need to comply with these standards when implementing PIPM to ensure the quality and safety of care”.*

In addition, the sense of responsibility mainly encompassed paying attention to PI prevention, emphasizing care quality, and not shirking responsibility. Nurses said: *“When faced with challenges in PIPM*,* nursing assistants need to proactively solve problems rather than avoid responsibility.”*

### Traits

Traits refer to the personal characteristics and qualities that influence a nursing assistant’s performance in PIPM. Personal traits are the essential, distinctive, and typical qualities that nursing assistants ought to exhibit while carrying out PIPM. The traits found in this study included carefulness and love, empathy and patience. Nurses said: *“Careful inspection of the skin is the key to early detection of PI and their risks. Each patient’s skin condition is unique*,* and careful inspection can help nursing assistants understand the individual differences of patients and provide personalized care plans.”* Nursing assistants mentioned: *“Elderly patients may not be able to fully control their behavior due to physical or cognitive limitations. We should treat them with respect and understanding with warm hearts*,* rather than blaming them.” “Elderly people may become slow to respond for various reasons. We need to treat them with patience. Patience can help me build a trusting relationship with the elderly and make them feel at ease.”*

### Motives

Motives refer to the underlying reasons and incentives that drive nursing assistants to perform their duties effectively in PIPM. Most nursing assistants and key stakeholders believed that nursing assistants succeeded in performing PIPM when they had potential professional development opportunities or were driven by supportive institutional policies such as assessment and evaluation mechanisms, continuing education credits, and reward mechanisms. Nursing assistants said: *“I want to make progress in my career*,* which motivates me to continuously improve my work standards. I hope to be a leader in the team.”* Policy-maker mentioned: *“Through regular and systematic assessment*,* we can ensure that the prevention and management measures for PI are effectively implemented. The assessment results help us identify the training needs of nursing assistants in PIPM*,* to motivate them to get targeted education and training.”* Nursing assistants said: *“The reward mechanism can significantly improve our work enthusiasm and encourage us to provide higher quality nursing services.”*

## Discussion

The qualitative study utilizing the Iceberg Model has shed light on the multifaceted competencies required by nursing assistants in the PIPM in nursing homes. Using the Iceberg Model, we classified theoretical knowledge and comprehensive skills as surface characteristics, while self-concept, traits, and motives were identified as hidden characteristics. Surface-level theoretical knowledge and comprehensive skills are easily understood, measured, and developed through training [26, 27]. In contrast, deeper-level self-concept, traits, and motives are harder to measure and less susceptible to change through external influences, yet they significantly impact behavior and performance [26, 27].

### The surface characteristics: theoretical knowledge and comprehensive skills

Two sub-competencies are included in the theoretical knowledge domain, namely basic and professional PIPM theoretical knowledge, which comprise 16 codes. This aligns with the work of He et al. [33], who explored oncology nurse competency in chimeric antigen receptor T-cell therapy employing the Iceberg Model and emphasized the need for a strong theoretical base to inform practice. Our study’s participants stressed the importance of understanding PI basic concepts, mechanisms, risk factors, and the significance of early identification, which is crucial for developing personalized care plans and conveying the importance of PI prevention to patients and families. This knowledge equips nursing assistants to recognize early signs of PI and to take appropriate preventive measures. These abilities conform to the theoretical knowledge requirements of National Occupational Skills Standards for Nursing Assistants of Elderly Care (2019 edition), which was released by the Ministry of Civil Affairs of the People’s Republic of China [34].

Comprehensive skills-related competencies consist of four sub-competencies: practical skills, communication skills, collaboration skills, and observation skills, and 11 codes were included. Practical skills such as turning and repositioning patients, wound dressing change techniques, and the use of walking aids are non-negotiable for effective PIPM. These skills are consistent with the 2019 International Clinical Practice Guidelines for the Prevention and Treatment of PI, which emphasize the importance of regular turning and repositioning especially for patients at high risk of developing PI, selecting appropriate dressings based on the type and stage of the wound, promoting mobility and independence to reduce the risk of PI [5]. Nursing assistants should be well-trained to provide high-quality care and prevent the development and progression of PI. Communication and collaboration skills are vital for a cohesive care team, while observation skills are paramount for early detection and intervention. These abilities empower nursing assistants to implement effective care strategies, function efficiently within multidisciplinary teams, and closely monitor the physical and psychological well-being of elderly residents [35]. The comprehensive skills are similar to those described in other studies for nursing assistants’ roles and job descriptions in PIPM, such as continuous monitoring, implementing a plan of care with the supervision of registered nurses, skin assessment, notifying nurses of skin changes, initiating the use of the mattress, repositioning patients and documenting, moisture, skin care, and so on [14, 15]. Additionally, Frances [36] examined organizational leadership and the communication and collaboration between registered nurses and certified nursing assistants in LTC facilities. The study highlighted the significance of effective communication and teamwork between nursing assistants and nurses in enhancing job performance, self-efficacy, and the overall work environment. Previous studies also have highlighted the pivotal role of nurses in interdisciplinary teamwork, with nursing assistants being key collaborators [37]. Effective collaboration especially the seamless information exchange between nurses and nursing assistants is crucial for delivering comprehensive, high-quality care [38].

These findings suggest that future research should focus on developing and implementing integrated training programs that enhance both the theoretical knowledge and comprehensive skills of nursing assistants in PIPM. These programs should be designed to align with national and international guidelines and standards, ensuring that nursing assistants are well-equipped to provide evidence-based care. Additionally, studies should evaluate the long-term impact of these training programs on patient outcomes, job satisfaction, and the overall quality of care in LTC settings.

### The hidden characteristics: self-concept, traits, and motives

The study underscores the importance of self-concept, including professional identity, ethical awareness, and a sense of responsibility, in shaping nursing assistants’ attitudes to PIPM. Our study’s participants expressed the need for a strong professional identity, including a passion for elderly care and commitment to work, which are essential for providing quality care and embracing the responsibilities inherent in PIPM. This is in line with Fergusson et al. who discussed the importance of professional identity in competency frameworks [39]. Previous studies have shown that residents were more satisfied with their relationships with nursing staff and their quality of life in units where a greater proportion of nursing assistants demonstrated strong job commitment [40]. In addition, the participants stressed that ethical awareness was crucial for nursing assistants, which aligns with other nursing-related fields. Ethical codes provide guidance that shapes ethical behavior, defining which values and beliefs should be upheld. These codes serve as practical guidelines in the nursing profession [41]. Tolosa et al. emphasized the need for health education and training on “nursing codes of ethics” to enhance the practice of ethical standards among nursing staff [42].

Personal traits such as carefulness and love, and empathy and patience, along with motives like professional development opportunities and supportive institutional policies, emerged as key drivers of quality care in PIPM. These findings resonate with Oeseburg et al., who noted that personal attributes significantly influence patient care outcomes [12]. Carefulness and love are foundational traits that ensure nursing assistants perform their duties with precision and compassion. For example, Careful inspection of patients’ skin is crucial for early detection of PI and their risks [5]. Empathy and patience are essential for building trust and smooth relationships with patients and families, particularly those who may be experiencing pain or discomfort. Empathetic nursing assistants can better understand and respond to residents’ emotional needs, providing comfort [43]. Apart from these traits, the desire for professional development opportunities such as competency enhancement and job promotion, along with supportive institutional policies such as evaluation mechanisms, continuing education opportunities, and reward mechanisms are powerful motivators for nursing assistants to excel in their roles. This aligns with a previous study that highlighted the importance of basic motivational factors for nursing assistants, such as opportunities for job enrichment, personal growth, recognition, and a sense of accomplishment [44]. This finding suggests that nursing homes should prioritize creating a conducive environment that fosters professional growth and recognizes the contributions of nursing assistants.

### Limitations

While this study provides valuable insights, it has several limitations that should be considered. First, our study involved a relatively small sample size, with 12 nursing assistants and 11 key stakeholders participating. While this sample provided rich and detailed data, it may limit the generalizability of our findings to broader populations. Second, participants were recruited from a single province in China, which may not fully represent the diverse experiences and perspectives of nursing assistants and stakeholders in other regions or countries. Third, we employed a purposive convenience sampling method, which may introduce selection bias, potentially leading to a non-representative sample. Future research should aim to address these limitations by using larger, more diverse samples, more rigorous sampling techniques, and considering the influence of cultural and contextual factors. This will enhance the generalizability and reliability of the findings, ultimately contributing to the development of comprehensive nursing assistant PI-related management and policies to improve the healthcare system.

## Conclusions

Our study developed a PIPM competency framework for nursing assistants through a focus group and semi-structured interviews grounded in the Iceberg Model. This framework comprises five main categories, 13 subcategories, and 47 codes. The findings provide valuable insights for the future evaluation and training of nursing assistants in PIPM, guiding the development of competency-based training programs and assessment tools. Future studies should expand the competencies needed by nursing assistants, such as exploring the technological competencies (the use of electronic health records, telehealth, and smart devices for PIPM) required by nursing assistants with the increasing integration of technology in healthcare. It is also recommended to examine the long-term impacts of well-trained nursing assistants on patient outcomes, which will help evaluate the benefits of targeted training programs.

## Electronic supplementary material

Below is the link to the electronic supplementary material.

Supplementary Material 1

Supplementary Material 2

## Data Availability

The datasets generated or analyzed during the current study are not publicly available but are available from the corresponding author upon reasonable request.

## Abbreviations

PI: Pressure injuries
PIPM: Pressure injury prevention and management
LTC: Long-term care
