# Supplementary material for: Exploring nursing assistants’ competencies in pressure injury prevention and management in nursing homes: a qualitative study using the iceberg model
Source: BMC Nurs. 2025 Mar 27;24:333. doi: 10.1186/s12912-025-02911-6 (PMC11948734; doi:10.1186/s12912-025-02911-6)
Supplement: Supplementary file 1 — Supplementary Material 1 [file 12912_2025_2911_MOESM1_ESM.zip › Community nurse in-depth interview transcript.docx]

**Community nurse in-depth interview transcript**

**Interviewer:**

Hello, Mrs ***. I am from ***. My name is ***. We are currently doing a study to gain an in-depth understanding of the nursing assistant's pressure injury prevention and management capabilities, training status, training needs and training suggestions from the perspective of community nurse, so as to provide a reference for nursing homes to formulate feasible training plans and carry out pressure injury management. During this interview, we need to record the entire interview process, but all information will be kept confidential, personal information will not be disclosed, and the interview content will only be used for research. Are you willing to participate in this interview?

**Interviewee:**

OK, no problem

**Interviewer:**

Thank you very much. Here is an informed consent form. Please sign it.

**Interviewee:**

OK

**Interviewer:**

First, please introduce your professional background and work experience, especially the experience related to the prevention and management of pressure injury.

**Interviewee:**

I am a community nurse. I graduated from a local nursing school. I have been working since 2011 and have 13 years of nursing experience. In my work experience, the prevention and management of pressure injury has always been a very important part. I have participated in the health management of many elderly people in the community. In these works, I pay special attention to their skin conditions. For those elderly people who have been bedridden for a long time or have difficulty in moving, I will regularly check their bodies to see if there are signs of pressure injury and take preventive measures in time. I will also teach family members and nursing assistants some knowledge and skills of pressure injury prevention, such as how to turn over correctly and keep the skin clean and dry. At the same time, I will develop personalized care plans according to the specific conditions of the elderly to ensure their skin health. In my contact with patients with pressure injury, I have accumulated rich experience and can detect problems in time and take effective measures to deal with them. I know the importance of pressure injury prevention and management, and I will continue to work hard to provide better care services for community residents.

**Interviewer:**

Have you ever participated in or provided training on pressure injury prevention and management? If so, what types of training or courses have you provided?

**Interviewee:**

I have also had experience in training nursing assistant in nursing homes. During the training, I will explain in detail the causes, hazards, prevention and treatment methods of pressure injury. I will emphasize that nursing assistant should carefully observe the skin condition of the elderly, turn them over and massage them in time, and keep their skin clean and dry. I will also guide nursing assistant on how to use nursing equipment correctly to reduce the risk of pressure injury. At the same time, I will also share some cases from actual work so that nursing assistant can more intuitively understand the importance of pressure injury prevention and management. Through these trainings, I hope that nursing assistant can improve their professional quality and provide better care services for the elderly. In my contact with patients with pressure injury, I have accumulated rich experience, and can detect problems in time and take effective measures to deal with them. I know the importance of pressure injury prevention and management, and I will continue to work hard to provide better care services for community residents.

**Interviewer:**

What role do you think nursing assistant in nursing homes play in the prevention and management of pressure injury?

**Interviewee:**

In the prevention and management of pressure injury, nursing assistant in nursing homes can be said to play a very important role. They are the people who live with the elderly day and night and have the most frequent contact with them, and can very keenly observe the subtle changes in the elderly's skin. The careful care and timely treatment of nursing assistant are of vital importance to reducing the risk of pressure injury. They need to turn the elderly over regularly according to scientific and correct methods to avoid long-term pressure on the same part of the body; they also need to carefully clean the skin of the elderly to keep the skin dry and clean; at the same time, they need to pay attention to the nutritional status of the elderly and ensure that they take in enough nutrition to enhance the resistance of the skin. In their daily work, nursing assistant also need to pay attention to the physical condition of the elderly at all times. Once they find signs of pressure injury, they must take immediate measures and provide feedback to our community nurses in a timely manner. Our community nurses will discuss with them based on the information they provide to develop a more complete and targeted care plan. It can be said that nursing assistants play an indispensable role in all aspects of pressure injury prevention and management. Their efforts and contributions are directly related to the health and quality of life of the elderly.

**Interviewer:**

What specific competencies do you observe in nursing assistants that contribute most to effective PIPM?

**Interviewee:**

Nursing assistants in nursing homes should have multiple capabilities in the prevention and management of pressure injury. Nursing assistants need to know what PI is and why it is an important indicator of quality control in nursing home management. Therefore, they need to understand the current epidemiological characteristics of PI, the prevalence of PI in hospitals, communities, and nursing homes, and the prevalence of PI in the elderly population. They need to know this data to better implement PIPM. Nursing assistants need to know what PI is and why it is an important indicator of quality control in nursing home management. Therefore, they need to understand the current epidemiological characteristics of PI, the prevalence of PI in hospitals, communities, and nursing homes, and the prevalence of PI in the elderly population. They need to know this data to better implement PIPM. Nursing assistants need to understand the pathogenesis can help nursing assistant detect the symptoms of PI early and intervene in time. Timely prevention and treatment can improve the overall health and prognosis of patients. Understanding the pathogenesis can help nursing assistants identify the signs of PI early and intervene in time. Timely prevention and treatment can improve the patient's overall health and prognosis

They need to have extremely keen observation skills and be able to observe the skin condition of the elderly in detail, even the slightest change cannot be missed. This observation ability allows them to detect potential risks of pressure injury at the first time and take preventive measures in time. In terms of turning over, nursing assistants should be proficient in various correct turning over techniques, and reasonably arrange the time and frequency of turning over according to the physical condition and needs of the elderly, to ensure that all parts of the elderly's body can be properly supported and avoid prolonged pressure on a certain part. Moreover, in the process of turning over for the elderly, the movements should be gentle and smooth to avoid discomfort or injury to the elderly due to improper movements. It is also crucial to keep the elderly's skin clean and dry. Nursing assistants should know how to choose appropriate cleaning products and clean the skin of the elderly in a gentle way. At the same time, they should pay attention to keeping the skin dry and changing damp clothes and bedding in time. In addition, it is necessary to understand the characteristics of different skin types in order to better care.

In addition, nutrition is very important for the elderly, especially for wound healing. Proper nutrition can enhance the resistance of skin and tissues and reduce the risk of PI. Knowledge of nutrition enables nursing assistants to provide personalized dietary advice based on the patient's specific situation. Nursing assistants can teach patients and families about the importance of nutritional care and help them understand how to improve their health through diet.

**Interviewer:**

Anything else?

**Interviewee:**

In addition, It is essential for nursing assistants to master basic wound dressing techniques to provide high-quality care, which helps ensure that patients' wounds are properly managed. Nursing assistants are an important part of our medical team. Their mastery of this technique allows them to collaborate more effectively with doctors and nurses to promote patient recovery. Effective communication skills are also essential. Nursing assistants should be able to communicate smoothly with the elderly and their families, explain to them the importance and methods of pressure injury prevention, and let them actively cooperate with nursing work. At the same time, they should be good at listening to the opinions and needs of the elderly and their families, timely feedback on the elderly's situation, and work together for the health of the elderly. Patience and responsibility are also qualities that nursing assistants should possess. They should treat every elderly with great patience and love, not be afraid of dirt or tiredness, and do every nursing work carefully and meticulously. In the process of pressure injury prevention and management, various measures should be strictly implemented, not perfunctory or coping, to ensure the safety and health of the elderly. In addition, nursing assistants should continue to learn and update the knowledge of pressure injury prevention and management, and improve their professional quality by participating in training, studying professional books, etc., so as to better cope with various challenges in their work. Only with these abilities can nursing assistants in nursing homes truly prevent and manage pressure injury and provide better care services for the elderly.

**Interviewer:**

What is your perspective on the importance of nursing assistants' attitudes or values towards PI prevention?

**Interviewee:**

In my opinion, the attitudes or values of nursing assistants in PI prevention are extremely important. A strong sense of responsibility is very important. Nursing assistants must deeply realize their responsibilities and regard PI prevention as a vital task in their work. They should take every detail of care seriously, from daily turning over, body position adjustment, to skin cleaning and care, and they must be meticulously implemented. Only with a high sense of responsibility can they always remain vigilant, discover potential risks in time, and take effective preventive measures to ensure the physical safety and health of the elderly. In addition, they should have a caring and respectful attitude. Nursing assistants should treat every elderly with sincere emotions and respect. They should understand the physical condition and psychological needs of the elderly, listen to their demands attentively, and pay attention to their emotional changes. Caring for the elderly with a caring heart and making them feel warm and cared for can not only enhance the trust and cooperation of the elderly, but also facilitate the smooth implementation of PI prevention. Respect the personality and living habits of the elderly, create a comfortable and warm nursing environment for them, and let the elderly spend every day in love and respect. It is essential to have professionalism. Nursing assistants need to have a rigorous professional attitude and constantly learn and update their knowledge and skills about pressure injury prevention. They should be familiar with the causes of pressure injury, prevention methods and treatment processes, and be able to use scientific theories and practical experience to guide their work. In actual operations, they should strictly follow the norms and standards and not act blindly to ensure the effectiveness and safety of preventive measures. Through continuous learning and improvement, they will continuously improve their professional quality and provide solid professional support for pressure injury prevention.

**Interviewer:**

Are there any other abilities?

**Interviewee:**

Teamwork is also very important. Nursing assistants should fully realize that they are a member of the nursing team and work closely with other nursing assistant to work together for pressure injury prevention. At work, they should cooperate and support each other, share experiences and information, and form a good working atmosphere. Everyone works together to jointly cope with various challenges in pressure injury prevention, improve the overall nursing level, and provide better services for the elderly. Being proactive is a necessary quality. Nursing assistants should be aware of proactively discovering problems and take proactive measures to solve them. We cannot wait for problems to occur or rely on others to solve them, but should proactively look for potential risk factors and take preventive measures in a timely manner. In the prevention of pressure injury, we must maintain a positive attitude, be brave to try new methods and strategies, and constantly explore and innovate to improve the prevention effect. Patience and carefulness are indispensable. Pressure injury prevention is a meticulous job that requires nursing assistants to have enough patience and care. They should patiently explain the importance and methods of pressure injury prevention to the elderly so that they can understand and actively cooperate. During the nursing process, we should carefully observe the skin condition of the elderly and not miss any subtle changes. We should take every care link seriously, ensure that every detail is in place, and not miss any factors that may cause pressure injury. Only with patience and carefulness can we really do a good job in pressure injury prevention.

**Interviewer:**

what personality traits do you think drive nursing assistants to be proactive in PIPM?

**Interviewee:**

In my opinion, they need to have a strong sense of responsibility. They regard the prevention of pressure injury as an important mission they shoulder, and treat every nursing work meticulously and seriously. Whether it is daily nursing operations or attention to the physical condition of the elderly, they will do their best and never be careless. This sense of responsibility allows them to always be alert, take the initiative to find problems, and take preventive measures to ensure the health and safety of the elderly.

In addition, they need to have keen observation and carefulness. They can observe the physical condition of the elderly in detail, including subtle changes in skin color, temperature, humidity, etc. At the same time, they can also pay attention to changes in the elderly's living habits, emotional state, etc., so as to timely discover potential risks of pressure injury and take corresponding measures quickly. They will carefully take care of every inch of the elderly's skin, provide them with meticulous care, and make them feel warm and caring.

The heart of caring for others is full of deep care for the elderly. This emotion prompts them to take the initiative to understand the needs of the elderly and care about the feelings of the elderly. They will take the initiative to communicate with the elderly, listen to their voices, and give them spiritual support and comfort. They will carefully provide a comfortable nursing environment for the elderly, so that they can spend every day in a warm atmosphere. This kind of care makes them willing to make more efforts to protect the health of the elderly.

**Interviewer:**

You just mentioned responsibility, love, carefulness, observation, etc. Do you need other personal qualities?

**Interviewee:**

They also need a strong sense of teamwork. They understand that they are part of the team and actively cooperate with other nursing assistants to work together to prevent pressure injury. They will take the initiative to communicate with colleagues, share experiences and insights, and support and help each other. They will focus on the interests of the team, jointly provide high-quality nursing services for the elderly, and create a good working atmosphere.

**Interviewer:**

How do institutional culture and policies influence nursing assistants' motivation to perform PIPM?

**Interviewee:**

Institutional culture and policies have an important impact on nursing assistants' motivation to perform PIPM. First, a positive institutional culture can create an atmosphere of caring for patients and focusing on nursing quality, which will subtly influence the values and work attitudes of nursing assistants and make them more motivated to do a good job in pressure injury prevention. If the institutional culture emphasizes teamwork and professionalism, nursing assistants will feel that they are valued and supported, and thus have more confidence and enthusiasm to perform their duties. Reasonable policies and rules and regulations can provide clear guidance and norms for nursing assistants, so that they know what should be done and what should not be done. This helps to cultivate their sense of responsibility and self-discipline, so that they will more consciously implement pressure injury prevention measures. At the same time, some incentive policies, such as reward mechanisms, can also stimulate the enthusiasm of nursing assistants and encourage them to work harder. In addition, the training and education policies of the institution also have a great impact on the ability improvement and enthusiasm of nursing assistants. If the institution provides rich training resources and learning opportunities, nursing assistants can continuously improve their professional knowledge and skills, which will make them more confident and willing to take proactive actions when doing pressure injury prevention. In short, institutional culture and policies largely shape the work attitude and enthusiasm of nursing assistants, and play a vital role in their prevention and management of pressure injury.

**Interviewer:**

What motives would further empower nursing assistants to perform PIPM effectively?

**Interviewee:**

When they see that the patient's pressure injury are prevented or improved through their own efforts, they will gain a great sense of professional achievement, which motivates them to work more actively. The second is the desire for professional growth, hoping to continuously improve their professional ability, accumulate experience, improve skills, and achieve self-growth through the effective implementation of PIPM. In addition, the recognition and encouragement of the team, the affirmation and praise from team members will make them feel that their work is recognized, and thus more motivated to do a good job. The gratitude of patients and their families, a thank you and a smile from patients and their families can become their motivation to continue working hard.

**Interviewer:**

Anything else?

**Interviewee:**

Provide training, learn new knowledge and skills, so that they can constantly update their cognition and better serve patients. A good working environment and resource support, sufficient nursing supplies, reasonable work arrangements, etc., can enable them to carry out their work more smoothly and have more motivation to do a good job.

**Interviewer:**

Okay, thank you very much for your answer. The next question is about pressure injury training. Could you please talk about the current situation of your institution's training on pressure injury prevention and management for nursing assistants?

**Interviewee:**

Some nursing homes regularly organize relevant training courses, covering the causes of pressure injury, prevention measures, assessment methods, and nursing skills. These trainings help nursing assistants improve their professional knowledge and skills and better cope with pressure injury prevention and management work. However, some institutions do not do enough in terms of training, the frequency of training may not be high enough, or the training content is not in-depth and comprehensive enough. Some nursing assistants may simply understand some basic concepts, but when they encounter specific problems in actual operations, they will still feel confused and overwhelmed. In addition, the training format is relatively simple, mostly focusing on theoretical explanations, lacking practical operations and case analysis, which also affects the understanding and mastery of knowledge by nursing assistants. I hope that in the future, nursing homes will pay more attention to the training of nursing assistants, and constantly improve the content and methods of training, so that nursing assistants can truly master the effective methods of pressure injury prevention and management, and provide better care services for the elderly.

**Interviewer:**

Okay, can you please talk about the training needs and suggestions for pressure injury?

**Interviewee:**

First, it is necessary to strengthen in-depth training on pressure injury prevention knowledge, including the mechanism of pressure injury formation, the manifestation characteristics of different stages, etc., so that we can more clearly understand the severity of pressure injury and the importance of prevention. Secondly, the training of practical operation skills is also critical, such as how to correctly turn the patient over, massage, and choose appropriate care products, etc. These skills need to be constantly practiced and strengthened. In addition, detailed explanations and training are also needed for the use of pressure injury risk assessment tools so that we can accurately assess the patient's pressure injury risk. In terms of suggestions, I hope that the training can pay more attention to practical operations, increase case analysis and discussion links, so that we can better combine theoretical knowledge with practical work. At the same time, the frequency of training can be appropriately increased to keep us familiar with the knowledge. We can also invite some experienced experts to share and guide us to broaden our horizons and ideas. I hope these suggestions can help with pressure injury training.

**Interviewer:**

Okay, you just mentioned the continuous training of knowledge and technology, training form suggestions, practical training and multidisciplinary cooperation. Is there anything else you need to add?

**Interviewee:**

No more at the moment

**Interviewer:**

Okay. Thank you very much for participating in this interview. You have discussed the current situation of nursing homes, the current situation of pressure injury training, the pressure injury capacity requirements of nursing assistants, the pressure injury training requirements and suggestions. If you have anything else to add later, please feel free to contact me. Thank you very much!
