# Supplementary material for: Exploring nursing assistants’ competencies in pressure injury prevention and management in nursing homes: a qualitative study using the iceberg model
Source: BMC Nurs. 2025 Mar 27;24:333. doi: 10.1186/s12912-025-02911-6 (PMC11948734; doi:10.1186/s12912-025-02911-6)
Supplement: Supplementary file 1 — Supplementary Material 1 [file 12912_2025_2911_MOESM1_ESM.zip › Focus group transcript.docx]

**Focus Group Transcript**

**Interviewer：**

Hello, nursing assistants. First of all, let me introduce myself. I am a nursing teacher from ***. My name is ***. I am currently doing a study. The purpose is to provide a reference for nursing homes to formulate feasible training programs and carry out pressure injury management by deeply understanding and exploring the nursing assistant's cognition of pressure injury prevention and management, existing obstacles, pressure injury prevention and management ability requirements, pressure injury training status, training needs and suggestions. During this interview, we need to record the entire interview process, but all information is confidential and personal information will not be disclosed. The content of the interview is only for research use and will not involve any assessment of you by the institution. Please do not feel pressured and express your views on the questions raised truthfully. Are you willing to participate in this interview? If you agree, please sign this informed consent form. Thank you very much for your cooperation!

**Interviewee：**

Agree， No problem

**Interviewer：**

Okay, thank you very much. First of all, I need to explain to you that after I ask a question, you can express your own opinions according to your own situation. There is no right or wrong in everyone's opinions. If you have something to add or emphasize after others have answered the relevant questions, you can always make additional explanations and explanations. If you have some questions that you don't understand, you can ask them at any time and I will explain them further. I hope you can express your opinions without reservation.

First of all, the first open question is, what is your daily work content in the nursing home?

**Interviewee1：**

We are involved in a lot of work, mainly some daily life care, such as daily life care, helping the elderly with daily life activities, such as dressing, washing, eating, going to the toilet, etc. There is also health monitoring, regularly checking the vital signs of the elderly, such as blood pressure, body temperature, pulse, etc., as well as observing the health status of the elderly, and promptly discovering and reporting abnormalities.

**Interviewee2：**

There is also medication management, distributing medications to the elderly according to doctor's orders and ensuring that they take medications on time. Rehabilitation assistance, assisting the elderly with rehabilitation training, such as physical therapy, exercise therapy, etc.

**Interviewee3：**

We also provide psychological comfort, such as emotional support, communication with the elderly, and help them relieve loneliness and anxiety. Of course, we have to do some environmental cleaning work to keep the elderly's living environment clean and hygienic, including rooms and toilets.

**Interviewer：**

Are there any other nursing assistants who would like to add?

**Interviewee4：**

They will also participate in some emergency situations. For example, when the elderly encounter sudden situations such as falls, choking, coughing, burns, and illness attacks, they can quickly take appropriate first aid measures.

**Interviewee5：**

As for some records and reports, we nursing assistants also need to write and record the daily activities, health conditions and any special events of the elderly, and report to the management or family members regularly. We also participate in the guidance of other staff members and collaborate with other nursing assistants, doctors, nutritionists and other professionals to provide comprehensive care services for the elderly.

**Interviewer：**

So what are the characteristics of the elderly people in the nursing homes that you care for?

**Interviewee6：**

As a nursing assistant in a nursing home, I have observed that the elderly people we care for usually have the following characteristics: As they age, their physical functions gradually decline, including vision, hearing, and mobility. Some elderly people completely lose their ability to take care of themselves and are bedridden for a long time. Many elderly people suffer from one or more chronic diseases, such as high blood pressure, diabetes, heart disease, etc., and need regular medication and monitoring.

**Interviewee7：**

Older adults may feel lonely and neglected, and they need emotional support and companionship. As they age, older adults may experience memory loss and sometimes even cognitive impairment. Some older adults may need help with activities of daily living, such as dressing, eating, and bathing.

**Interviewee8：**

Another thing is that due to physical and health limitations, older people may have reduced social activities, which may affect their mental health.

**Interviewer：**

Just now you talked about a lot of daily care work, and I also heard that the prevention and management of pressure injuries is also a very important nursing task in your work. Could you please describe your understanding of pressure injuries, including its definition and harm, and how important do you think pressure injury prevention is in your role as a nursing assistant?

**Interviewee9：**

As a nursing assistant in a nursing home, I know that the prevention and management of pressure injuries is a key point in our daily work. Pressure injuries refer to skin damage caused by long-term bed rest. They usually occur on the coccyx, ischium, ankles, heels and elbows. Pressure injuries are easily infected and may lead to local or systemic infection, and in severe cases may even be life-threatening.

**Interviewee1：**

Pressure injuries are caused by continuous external pressure, which can cause pain and affect the quality of life of the elderly. The cost of treating pressure injuries is high, which increases the financial burden on families. Pressure injuries can cause shame and depression in the elderly, affecting their mental health.

**Interviewee11：**

We take the prevention of pressure injuries very seriously because once they occur, they are difficult to treat and have a significant impact on the quality of life of the elderly. We regularly check the skin condition of the elderly, especially those areas at risk of pressure injuries, such as the coccyx, sciatic bones, ankles, heels and elbows.

**Interviewee2：**

We regularly help the elderly turn over and adjust their body positions to reduce pressure on the same part for a long time. We use pressure relief mattresses, cushions and other equipment to reduce pressure points.

**Interviewee12：**

We keep the elderly’s skin clean and dry, avoiding moisture and friction. We ensure that the elderly have good nutrition to promote skin health and the healing of pressure injuries.

**Interviewer：**

What difficulties, challenges or obstacles are encountered in daily pressure injury management?

**Interviewee5：**

In the daily management of pressure injuries, we as nursing assistants do encounter some difficulties and obstacles. For example, the resources of nursing homes may be limited, including professional nursing assistant, sufficient pressure relief equipment and appropriate care materials. Some elderly people may not understand the seriousness of pressure injuries and are unwilling to cooperate with turning over or using pressure relief equipment.

**Interviewee12：**

Yes, in addition to this, sometimes families may be skeptical about the prevention and management of pressure injuries or have misunderstandings about care. Elderly people may have multiple medical conditions, such as cognitive impairment, paralysis, or severe arthritis, which makes turning and position adjustment difficult. Insufficient care staff may result in an inability to frequently help the elderly turn over, which is a key measure to prevent pressure injuries.

Some nursing assistants may not have received adequate training in the prevention and management of pressure injuries, resulting in a lack of necessary knowledge in actual operations.

**Interviewer：**

Are there any other obstacles? Is there anything else you want to add?

**Interviewee8：**

Long working hours and frequent position adjustments may cause physical fatigue to nursing assistants and affect the quality of care. In multicultural nursing homes, cultural and language differences may affect the communication between nursing assistants and the elderly and their families.

**Interviewer：**

What everyone said is very good. The next question is about the need for nursing assistants to prevent and manage pressure injuries. what pressure injury knowledge and skills do nursing assistants need to care for the elderly?

**Interviewee6：**

As nursing assistants in nursing homes, in order to effectively prevent and manage pressure injuries, we need to have the following knowledge and skills: Understand the various stages of pressure injuries, including early signs and late symptoms, so as to identify and intervene in time. We can explain the severity and treatment process of pressure injury to patients and families if we understand the staging and clinical manfestation, helping them understand the importance of nursing measures. In addition, you need to be able to use pressure injury risk assessment tools, such as the Braden scale, to assess the risk of pressure injuries in the elderly. And you need to master the correct skin cleaning and care methods, including how to clean, moisturize and protect the skin.

**Interviewee2：**

Learn how to safely and effectively help older adults turn and reposition themselves to reduce stress. While helping patients turn over, we can check the skin condition and find redness, swelling or other abnormalities in time, which helps to identify the risk of PRESSURE INJURY early. Regular turning can reduce the discomfort caused by lying in the same position for a long time。Be familiar with various pressure relief devices, such as mattresses, cushions, wheelchair cushions, etc., and know how to use them correctly. Understand the importance of nutrition in the prevention and healing of pressure injuries, and how to encourage the elderly to consume adequate protein and other nutrients.

**Interviewee3：**

I think it is also necessary to master basic infection control measures, such as hand hygiene, wound care and the use of aseptic techniques. Some other related skills are also needed, such as being able to communicate effectively with the elderly and their families, explaining the importance of pressure injury prevention and care plans. Understand how to accurately record the skin conditions and care measures of the elderly, and how to cooperate with nurses to update the care plan.

**Interviewee6：**

We must master the correct transfer techniques to ensure that we transfer patients safely and efficiently, and avoid falls or other accidental injuries. If the transfer is not performed properly, it may cause pain or discomfort to the patient. We need to ensure that the transfer process is as smooth and comfortable as possible. We often need to use various assistive devices, such as wheelchairs, walkers, lifts, or pulley sheets, to help transfer patients.

**Interviewee1：**

We need to know who are the high-risk groups for pressure injuries, We must have the ability to identify risk groups. The high-risk groups for pressure injury we usually encounter at work are mainly patients with paralysis, hemiplegia, paraplegia, or cerebral palsy. Due to limited mobility, they are prone to pressure injury on bony protrusions. In addition, comatose, incontinent, and malnourished elderly people often develop pressure injury, so this group of people should be paid special attention to as nursing assistants。

**Interviewee5：**

Also be able to collaborate with other nursing assistants, physicians, and nurses to develop and implement care plans. pressure injury prevention and management is a team effort. We need to support each other, share responsibilities, and ensure that every nursing measure is properly implemented. Each nursing assistant has his or her own unique experience and skills. By working together, we can share each other's experiences and best practices and improve the level of care together. In daily work, timely information exchange is essential for patient care. We need to share the patient's condition changes and nursing needs with colleagues.

We need to implement pressure injury prevention and management according to the pressure injury prevention and management plan developed by nurses. Good communication ensures the consistency and coherence of the care plan. Other communication problems may also exist. Sometimes we encounter challenges in our work, such as it is difficult to communicate with some patients and their families, and we are not very good at using communication methods that they can accept to resolve conflicts encountered when implementing pressure injury prevention and management. For example, some patients are not very cooperative in turning over, changing clothes, wiping their bodies to keep their skin clean, etc. These will affect the occurrence of pressure injury.

**Interviewee2：**

I would also like to add that it is important to be able to provide psychological care, emotional support, and help the elderly cope with the psychological stress of pressure injuries. We need to realize that pressure injury are not only a physical condition, but also have an impact on the elderly's psychological state. Elderly people with pressure injury may feel depressed, anxious, or lose self-esteem. We need to provide emotional support and encouragement. By building a trusting relationship, we can help the elderly feel safer and more willing to accept care and treatment.

**Interviewee12：**

Learn how to assess and manage pain associated with pressure injuries. Master basic wound care skills, including cleaning, dressing and monitoring wound healing. Wound dressing is a basic nursing skill that we nursing assistants need to master in order to provide timely and appropriate wound care for our patients. Through proper cleaning and dressing changes, we can help keep wounds clean and create the best environment for healing.

**Interviewee5：**

We also need the ability to differentiate pressure injuries from other wounds. Sometimes it is difficult for us to identify whether it is a pressure injury skin problem during nursing care, and we are not sure to make a correct judgment. We don’t know whether it is a pressure injury, incontinence-related dermatitis or other skin problems. Therefore, we often cannot implement preventive measures in time。

**Interviewee11：**

We need to understand various wound dressings, including but not limited to film dressings, hydrogels, foam dressings, etc. Each dressing has its specific purpose. We need to choose the most suitable dressing according to the type, size, depth and amount of exudate of the wound. The choice of dressing should also take into account the comfort of the patient. Some dressings are not easy to damage the skin tissue when changing, which reduces the pain of the patient. On the premise of ensuring the effect, we also need to consider the cost-effectiveness of the dressing and choose cost-effective products for patients. However, since there are many types of wound dressings on the market, we sometimes cannot distinguish their functions, so the knowledge in this area is still lacking.

**Interviewer：**

what traits or characteristics must nursing assistants have to perform pressure injury prevention and management for the elderly?

**Interviewee11：**

I think you need to be passionate about the elderly care profession. We love our work and are committed to providing the best care for the elderly. We also have a sense of belonging. We see ourselves as part of the elderly care family and build close connections with colleagues and the elderly.

**Interviewee9：**

We must be responsible, dedicated, able to endure hardships and not be afraid of the difficulties of work. We often take care of patients without fear of hardship, taking care of more than 5 elderly people every day, working more than 8 hours a day. This is our job. Since we have chosen this job of caring for the elderly, we must be prepared to devote our time, energy and physical strength.

**Interviewee5：**

We must strictly abide by the laws and regulations related to elderly care to ensure the legality of nursing work. Respecting the dignity and privacy of older people helps build trusting relationships between patients and us, which is the foundation of effective care. When patients feel respected, we are more likely to actively cooperate with care interventions, including the prevention and management of pressure injury.

**Interviewee7：**

We need to recognize that each elderly person is a unique individual whose needs and preferences should be respected and considered. When elderly people feel they are treated equally, they are more likely to actively participate in our care plans, including pressure injury prevention measures.

**Interviewee3：**

Focus on providing high-quality care to ensure the health and comfort of the elderly. Do not shirk responsibility, take responsibility for your work, and do not shirk any responsibility. Check the elderly's skin regularly and carefully to detect and deal with problems in time. Encourage the elderly to live independently as much as possible.

**Interviewee4：**

We need to understand that older people may have various needs and challenges and not blame them for it. Actively pay attention to the needs of older people. Take the initiative to understand and meet the needs of older people. Be able to think from the perspective of older people. Put yourself in the shoes of older people and understand their feelings. Elderly patients may not be able to fully control their behavior due to physical or cognitive limitations. We should treat them with respect and understanding, rather than blaming them. When we avoid blaming and adopt an encouraging and supportive approach, patients are more willing to open up and share their feelings and needs。

**Interviewee6：**

Thinking from the perspective of the elderly allows me to better understand their feelings and needs, which is a manifestation of empathy. If I can understand the perspective of the elderly, communication will become smoother, and I can convey information more accurately and explain the importance of pressure injury prevention and management”

**Interviewer：**

Everyone has said a lot, is there anything to add?

**Interviewee8：**

I think we also need to be patient with the elderly. Understand that they may be slower to react, slower to think, slower to move, and be patient with that. Elderly people may become slow to respond for various reasons. We need to respect these individual differences and treat them with patience. Patience can help me build a trusting relationship with elderly patients and make them feel at ease and cared for

**Interviewer：**

OK, the next question is, what motivates you to prioritize pressure injury prevention in your daily work?

**Interviewee3：**

Pressure injury prevention requires professional knowledge and skills. Through continuous learning and practice, I am able to improve my nursing ability, which makes me feel proud and satisfied. Nursing can be challenging at times. However, when I thought these challenges could enhance my competency, I would have the motivation to conduct the nursing service. This belief has enabled me to maintain a positive attitude and not give up even when things get tough. For example, I attended a workshop on pressure injury prevention and learned about the latest research and care. Through this study, I learned about a new skin care product that can effectively reduce friction and shear forces, thereby reducing the risk of pressure injuries. Incorporating this product into my daily routine has not only improved my nursing skills, but also significantly improved the comfort of seniors.

**Interviewee8：**

In addition, in nursing homes, nursing assistants who can effectively prevent and manage pressure injuries are often more valued, which helps with career development and promotion. In my institution, a nursing assistant was promoted to nursing team leader because he successfully prevented many cases of pressure injuries. This example inspired me and made me realize that by focusing on pressure injury prevention, I can not only help the elderly, but also have a positive impact on my own career. I want to make progress in my career, which motivates me to continuously improve my work standards and ensure that every nursing care is of high standard. I hope to be a leader in the team, contribute to the team through my efforts and expertise, and jointly improve the level of nursing services

**Interviewee1：**

Organizations usually have assessment and evaluation mechanisms to measure the performance of nursing assistants. Making pressure injury prevention a priority can help you get good ratings in these evaluations. Our organization has a regular quality of care evaluation system, which includes the effectiveness of pressure injury prevention. In each evaluation, I record in detail the skin condition of the elderly I am responsible for and the preventive measures I take. This evaluation not only helps me improve my work methods, but also allows me to get good ratings in the evaluation.

**Interviewee4：**

Some nursing homes have a reward system to reward nursing assistant who have made significant contributions to pressure injury prevention. This is both recognition of individual work and an additional incentive. Our institution has an incentive program that provides bonuses or additional days off to nursing assistants who make significant contributions to pressure injury prevention. Last year, I received an award for successfully preventing a pressure injury in an at-risk senior citizen, which not only recognized my work but also motivated me to continue my efforts.

**Interviewee7：**

Participating in training and seminars related to pressure injury prevention can earn you continuing education credits, which is important for career advancement and maintaining professional qualifications. Last year, I took an online course on pressure injury management and earned continuing education credits. This course not only kept me up to date on the latest pressure injury prevention techniques, but also helped me earn the necessary credits to maintain my nursing license.

**Interviewee4：**

The cost of preventing and treating pressure injuries is very high. Through effective preventive measures, the consumption of medical resources can be reduced, which is also an important motivation for my work. Whenever I see that the skin condition of the elderly has improved through my efforts, or the occurrence of pressure injuries has been successfully avoided, I feel a great sense of accomplishment. In the team, we work together to prevent pressure injuries, and this team spirit is also one of the motivations for my work.

**Interviewee9：**

Through effective pressure injury prevention, I can help the elderly improve their quality of life and reduce their pain and discomfort, which is the most direct and meaningful motivation. I once cared for an elderly person who had been bedridden for a long time. By turning the patient regularly and using a pressure-relieving mattress, I successfully prevented the occurrence of pressure injuries. It was very satisfying and valuable to see that the elderly person maintained a better quality of life because he avoided the pain of pressure injuries.

**Interviewee11：**

As a nursing assistant, it is my duty to follow professional ethics and provide the best care for the elderly, which is also the driving force in my daily work. As a nursing assistant, I know the importance of following professional ethics. By focusing on pressure injury prevention, I can ensure that I provide ethical care for the elderly. This adherence to professional ethics makes me proud.

**Interviewer：**

what resources or support do you feel are necessary for effective pressure injury prevention and management?

**Interviewee10：**

If institutions can establish a reward mechanism to provide bonuses, extra leave or other forms of rewards to nursing assistants who perform well in pressure injury prevention, this will greatly improve the enthusiasm and passion of nursing assistants. The reward mechanism can significantly improve our enthusiasm and work enthusiasm and encourage us to provide higher quality nursing services. Through rewards, teamwork spirit can be promoted and we can be encouraged to collaborate with each other to jointly improve the effectiveness of pressure injury management

**Interviewee8：**

Publicly commending nursing assistants who have made outstanding contributions to pressure injury prevention at staff meetings can enhance their sense of professional honor. Regularly provide professional training on pressure injury prevention and management to help nursing assistants master the latest nursing knowledge and skills. Provide online learning resources to facilitate nursing assistants to improve themselves after work.

**Interviewer：**

OK, is there anything else you want to add? OK, if not, I will continue to ask the following questions. Could you please tell us about the current status of pressure injury training for nursing assistant?

**Interviewee2：**

Our institution organizes professional training on pressure injury prevention and management at least twice a year to ensure that nursing assistants' knowledge is continuously maintained. The training content usually includes the identification of pressure injuries, risk assessment, prevention measures, wound care and the latest clinical guidelines.

**Interviewer：**

What is the format of the training?

**Interviewee3：**

The training mainly includes face-to-face lectures and practical exercises. Usually, after the theoretical class in the classroom, students go to the training room for operational training and practice.

**Interviewer：**

Which teachers provided you with training?

**Interviewee4：**

The training is usually conducted by experienced nurses or external experts who bring a wealth of theoretical knowledge and practical experience. After the training, we will conduct an assessment to ensure that the nursing assistant has mastered the necessary knowledge and skills.

**Interviewer：**

Are there any problems with the current training?

**Interviewee4：**

At present, the content of training is relatively small, the time is relatively short, some trainers are not professional clinical experts, and the quality of training content is not uniform. Some training content is not very practical and cannot be used in clinical practice.

**Interviewee7：**

I feel that the training time is inappropriate. It is often conducted when we are resting or working, which disturbs our rest time and work.

**Interviewer：**

I feel that the training time is inappropriate. It is often conducted when we are resting or working, which disturbs our rest time and work.

**Interviewee6：**

I suggest conducting special training on pressure injuries instead of conducting unified training on other nursing knowledge. It will be more effective to conduct targeted training on pressure injuries.

**Interviewee8：**

I suggest that organizations provide more online training resources so that nursing assistants can learn flexibly after work.

**Interviewer：**

Thank you all nursing assistants for your kind participation. This focus group discussion is done. If you have any other ideas or opinions, very welcome to tell me. Thank you!
