# Supplementary material for: Exploring nursing assistants’ competencies in pressure injury prevention and management in nursing homes: a qualitative study using the iceberg model
Source: BMC Nurs. 2025 Mar 27;24:333. doi: 10.1186/s12912-025-02911-6 (PMC11948734; doi:10.1186/s12912-025-02911-6)
Supplement: Supplementary file 1 — Supplementary Material 1 [file 12912_2025_2911_MOESM1_ESM.zip › Geriatric nurse indepth interview transcript.docx]

**Geriatric nurse in-depth interview transcript**

**Interviewer:**

Hello, Mrs ***. I am from ***. My name is ***. We are currently doing a study to gain an in-depth understanding of the nursing assistant's pressure injury prevention and management capabilities, training status, training needs and training suggestions from the perspective of geriatric nurse, so as to provide a reference for nursing homes to formulate feasible training plans and carry out pressure injury management. During this interview, we need to record the entire interview process, but all information will be kept confidential, personal information will not be disclosed, and the interview content will only be used for research. Are you willing to participate in this interview?

**Interviewee:**

OK, no problem

**Interviewer:**

Thank you very much. Here is an informed consent form. Please sign it.

**Interviewee:**

OK

**Interviewer:**

First, please introduce your professional background and work experience, especially the experience related to the prevention and management of pressure injury.

**Interviewee:**

I have been involved in clinical work since 2000 and have been engaged in nursing. Around 2010, our hospital organized a skin pressure injury nursing team, and I was a member of it. In October 2013, I started working in the geriatric department. Most of the patients in the geriatric department are palliative care. We belong to primary care, a grassroots unit.

**Interviewer:**

Have you ever participated in or provided training on the prevention and management of pressure injury? If so, what types of training or courses have you provided?

**Interviewee:**

Yes, I have. One of our hospital is a national stoma nurse and another is a provincial stoma nurse.

**Interviewer:**

What role do you think nursing assistants play in the prevention and management of pressure injury?

**Interviewee:**

It is very important for nursing assistants to master the prevention and treatment skills of pressure injury. Because this is not only related to the patient's comfort and quality of life, but also can directly affect the patient's recovery process. Therefore, nursing assistantss have a comprehensive understanding of the concept of pressure injury, the cause of occurrence, the influencing factors and the harmfulness. This can well prevent the occurrence of pressure injuries.

**Interviewer:**

What do you think of the current status of nursing assistants in nursing homes in terms of their ability to prevent and manage pressure injury (knowledge, skills, attitude, communication, etc.)?

**Interviewee:**

Our hospital has a skin pressure injury team that regularly organizes activities in nursing homes, with online and on-site guidance. The guidance of nursing assistants can prevent and discover or take corresponding measures in a timely manner. For example, cleaning wounds, changing dressings, using drugs, etc. to prevent further deterioration of pressure injury. And they can also monitor the patient's condition changes and report to the doctor in time so that the doctor can adjust the treatment plan in time. In general, they now play an indispensable role in the prevention and management of pressure injury. Not only do they have solid professional knowledge and skills, but they also have a high sense of responsibility and compassion, so they can basically ensure that patients receive the best treatment and care.

**Interviewer:**

what specific competencies do you observe in nursing assistants that contribute most to effective PIPM?

**Interviewee:**

They need to understand the concept, classification, manifestations and risk factors of pressure injury. There are skin frictions of patients who have been bedridden for a long time, and incontinence causes the skin to be moist and sweaty. Prevention is to learn how to prevent pressure injury. For example, how to turn over correctly to keep the skin clean, and use appropriate mattresses and turning equipment. Nursing assistants need to fully understand the concept of pressure injury, the causes of occurrence, the influencing factors, and the harmfulness of pressure injury, so as to increase their awareness of pressure injury. Nursing assistants should master how to develop personalized pressure injury prevention plans based on the patient's physical condition and condition, including regular turning over, using auxiliary tools such as pressure injury pads, and keeping the skin clean and dry. For patients who have already developed pressure injury, nursing assistants need to learn the correct treatment methods, such as cleaning wounds, changing dressings, observing the condition, etc., to relieve the patient's pain and promote recovery.

In addition, with meticulous observation, they can very keenly perceive any subtle changes in the elderly's skin, whether it is a change in color, a difference in temperature, or a strange touch, and can capture it in time, which is a key step in preventing pressure injury. The ability of nursing assistants to observe skin conditions is crucial for early identification of PI. Early detection of skin changes can take preventive measures in advance. Good communication skills are also crucial. They can communicate effectively with the elderly and their families in a gentle and kind language, understand the needs and conditions of the elderly, listen to their voices patiently, and make the elderly and their families feel cared for and understood. At the same time, they can accurately convey relevant nursing information to ensure the smooth progress of the entire nursing process. Patience and love are their indispensable qualities. In the nursing process, they always maintain enough patience to treat the elderly and give them meticulous care and care. They are like warm sunshine, illuminating the hearts of the elderly and making them feel comfortable and at ease during the nursing process. Whether it is turning the elderly over, massaging, or assisting them in daily activities, they always maintain patience and love.

Skilled nursing skills are also necessary. They are proficient in the correct nursing operation skills such as turning over and skin care, and can provide reasonable care according to the specific conditions of the elderly. Teamwork ability is also needed. They can work closely with other medical staff to provide comprehensive nursing services for the elderly. In the team, they support and cooperate with each other, just like a close-knit big family, working together for the health of the elderly.

There is also a strong sense of responsibility. They are serious and responsible for the care of the elderly and do not neglect it. No matter how tedious the work is, they always stick to their posts and serve the elderly with all their heart. They put the health of the elderly first and interpret the true meaning of responsibility with their actions.

The prevention and management of PI requires close cooperation of a multidisciplinary team. Effective communication between nursing assistants and nurses is the basis of teamwork. Nursing assistants are often the first to discover problems in daily patient care. They need to communicate with nurses in a timely manner about any changes in the patient's skin condition, including risk factors for PI. The prevention and management of PI requires the close cooperation of a multidisciplinary team. Effective communication between nursing assistants and nurses is the basis of teamwork. Nursing assistants are often the first to discover problems in daily patient care. They need to communicate with nurses in a timely manner about any changes in the patient's skin condition, including risk factors for PI.

**Interviewer:**

Yes, yes. What is your perspective on the importance of nursing assistants' attitudes or values towards PI prevention?

**Interviewee:**

First, a positive attitude can make them take every nursing work more seriously, including carefully observing the skin condition of the elderly and changing the position in time, which is the basis for preventing pressure injury. If they do not have the right attitude, they may ignore some details and increase the risk of pressure injury. Secondly, the value of caring for the elderly can make them more empathetic and really take the pain and needs of the elderly to heart. They will take the initiative to care for the elderly and provide them with more considerate services, which is very important for improving the quality of life of the elderly. Furthermore, the value of responsibility makes them realize the importance of their work, not just completing tasks, but ensuring the health and safety of the elderly. This sense of responsibility will motivate them to continue learning and improving, and to enhance their preventive capabilities.

The quality of care is a key indicator of our service level. Nursing assistants must pay attention to the quality of care and ensure that every step meets the highest standards of care. Paying attention to the quality of care will help identify and implement effective PI prevention measures and reduce the occurrence of complications.

**Interviewer:**

What personality traits do you think drive nursing assistants to be proactive in PIPM?

**Interviewee:**

First, a strong sense of responsibility is the driving force behind their proactiveness. They regard the health of the elderly as their primary responsibility, and do every preventive work conscientiously and never slack off. Whether it is daily care or emergency handling, they can treat it with a high sense of responsibility to ensure the safety and comfort of the elderly. Carefulness is also an indispensable trait. They are able to observe every detail of the elderly's physical condition, skin condition, and even emotional changes in detail. When performing nursing operations, they will carefully check every link to ensure that there are no omissions and not miss any hidden dangers that may cause pressure injury. This careful and serious attitude allows them to detect problems in advance and take preventive measures in time. Patience is also one of their outstanding characteristics. When faced with tedious nursing work, they will not show impatience or impatience, but patiently provide continuous care for the elderly. Whether it is turning the elderly over, massaging, or assisting them in daily activities, they can complete it patiently, making the elderly feel cared for and warm. They understand that pressure injury prevention is a long-term process that requires patience and persistence. Caring for others is their emotional foundation for being proactive. By actively paying attention to the needs of the elderly, nursing assistants can detect potential PI risks as early as possible and take timely measures. When the needs of the elderly are paid attention to, they are more likely to actively participate in their own care process, including the prevention and management of PI. By actively paying attention to the needs of the elderly, nursing assistants can identify potential PI risks early and take timely measures. When the needs of the elderly are paid attention to, they are more likely to actively participate in their own care process, including the prevention and management of PI. They truly care about every elderly person and take their pain and needs to heart. Dedicated nursing assistants always put the needs of patients first, which helps ensure the effective implementation of PI prevention and management measures. This kind of care makes them willing to take the initiative to understand the situation of the elderly and provide more considerate services to the elderly. They will take the initiative to communicate with the elderly, care about their feelings, and make the elderly feel respected and cared for during the nursing process. Teamwork spirit is also one of their important qualities. They can work closely with other medical staff to work together for the health of the elderly. In the team, they actively participate in discussions and decision-making, share their experiences and insights, and contribute to the development of the team. They understand that pressure injury prevention is not a one-person thing, but requires the collaboration of the entire team.

**Interviewer:**

How do institutional culture and policies influence nursing assistants' motivation to perform PIPM?

**Interviewee:**

A positive institutional culture that focuses on patient safety and nursing quality will make nursing assistants feel the value and significance of their work, and thus be more motivated to do a good job in preventing pressure injury. In such a cultural atmosphere, nursing assistants will hold themselves to higher standards and take the initiative to learn and improve their abilities. At the same time, reasonable policies are also very important. For example, clear job responsibilities and work requirements will allow nursing assistants to clearly know what they need to do and to what extent; and a complete incentive mechanism, such as rewarding excellent nursing assistants, can stimulate their enthusiasm and creativity. In addition, providing sufficient training and resource support can also make nursing assistants more confident and capable of doing a good job in preventing pressure injury.

**Interviewee:**

Okay, then please tell us about your views on pressure injury training for nursing assistants in nursing homes.

**Interviewee:**

Pressure injury training for nursing assistants in nursing homes is very necessary and important. First of all, training can enable nursing assistants to better understand the causes, hazards and preventive measures of pressure injuries, which will help them pay more attention to the physical condition of the elderly in their daily work, take preventive measures in time, and reduce the risk of pressure injury. Secondly, through training, nursing assistants can master the correct nursing skills and methods, such as how to turn over and massage the elderly correctly, how to choose appropriate nursing supplies, etc., which can improve the quality of care and allow the elderly to receive more professional care. Furthermore, training can also enhance the nursing assistants' sense of responsibility and mission, make them aware of the importance of their work, and treat every elderly person more seriously and responsibly. In addition, training is also a platform for communication and learning, where nursing assistants can share experiences with each other and jointly improve their ability to cope with pressure injuries.

**Interviewer:**

What motives would further empower nursing assistants to perform PIPM effectively?

**Interviewee:**

First, recognition and appreciation can greatly motivate them. Whether it is affirmation from patients, family members, colleagues, or superiors, they will feel that their efforts are seen, and thus they will be more motivated to do better. Furthermore, the desire to continuously improve their professional ability is also an important motivation. When they realize that they can improve their professional level and better cope with various nursing challenges by doing a good job in pressure injury prevention, they will be more enthusiastic to get involved.

**Interviewer:**

Okay, could you please talk about the training needs and suggestions for pressure injury?

**Interviewee:**

In terms of training methods, I think a combination of online and offline training is more suitable. Online training resources are rich and diverse, including video tutorials on pressure injury prevention and management, online courses, professional websites, etc. These resources can be learned anytime and anywhere, making it convenient for medical staff to use fragmented time for self-improvement. Offline on-site teaching can be carried out in the form of lectures, seminars, etc., inviting experts in the field of pressure injury to give lectures and share experiences and techniques. In addition, visits to demonstration wards can be organized to conduct actual applications of pressure injury prevention and management. Offline practical operation links can set up simulation scenes to allow medical staff to perform pressure injury prevention and nursing operations under the guidance of mentors. Through practical operations, medical staff can more intuitively understand the prevention and management skills of pressure injury and improve their practical operation capabilities.

In terms of training time, I suggest that new medical staff should complete pressure injury training within three months after joining the company. For the rest, in principle, a pressure injury training course held every six months can basically meet the learning needs of nursing assistants.

Regarding the training effect detection method, I think that after the training, the training effect can be evaluated through questionnaires, practical assessments, etc., to understand the medical staff's mastery of the knowledge of pressure injury prevention and management and their actual application ability. According to the evaluation results, the weak links can be strengthened and improved. A follow-up guidance mechanism can also be established to regularly visit and guide medical staff to understand the problems and difficulties they encounter in actual work, and provide corresponding solutions and suggestions.

**Interviewer:**

Okay, thank you very much for your very detailed answer to the above questions. In addition to the above questions, do you have anything else to add?

**Interviewee:**

None at the moment

**Interviewer:**

Thank you for your valuable opinions and participation. Your opinions will help improve the management of pressure injury in nursing homes. If you have other information to add, please feel free to share.
