# Supplementary material for: Exploring nursing assistants’ competencies in pressure injury prevention and management in nursing homes: a qualitative study using the iceberg model
Source: BMC Nurs. 2025 Mar 27;24:333. doi: 10.1186/s12912-025-02911-6 (PMC11948734; doi:10.1186/s12912-025-02911-6)
Supplement: Supplementary file 1 — Supplementary Material 1 [file 12912_2025_2911_MOESM1_ESM.zip › Government policy-maker in-depth interview transcript.docx]

**Government policy-maker in-depth interview transcript**

**Interviewer:**

Hello, Mr ***. I am from ***. My name is ***. We are currently doing a study to gain an in-depth understanding of the nursing assistant's pressure injury prevention and management capabilities, training status, training needs and training suggestions from the perspective of policy-maker of Civil Affairs Bureau, so as to provide a reference for the nursing homes to formulate feasible training plans and carry out pressure injury management. During this interview, we need to record the entire interview process, but all information will be kept confidential, personal information will not be disclosed, and the interview content will only be used for research. Are you willing to participate in this interview?

**Interviewee:**

Okay

**Interviewer:**

First, I would like to ask about your professional background. Please introduce your work organization, including the scale, type, and service objects of this organization.

**Interviewee:**

I graduated from Nanjing with a degree in elderly care services and management. I am currently in charge of the formulation of elderly care-related policies at the Civil Affairs Bureau. I have been working for 13 years. Our work organization is one of the important functional departments of the government. Our organization is mainly responsible for the planning, guidance and supervision of elderly care services in the region. In terms of the scale of the organization, we have a professional team, including policy researchers, managers and service personnel. Regarding the type of organization, we belong to an administrative management organization. In terms of the elderly care institutions we serve, we cover all types of elderly care institutions in the region, including public elderly care institutions, private elderly care institutions, etc., and are committed to promoting the comprehensive development of elderly care services and improving the quality of life and happiness of the elderly.

**Interviewer:**

Okay, then what difficulties or challenges do you think there are in the prevention and management of pressure injury in elderly care institutions.

**Interviewee:**

There are indeed some difficulties and challenges in the prevention and management of pressure injury in elderly care institutions. First of all, some elderly care institutions lack awareness of the importance of pressure injury prevention, lack systematic and professional nursing knowledge and skills training, and nursing assistant have uneven awareness and ability in preventing pressure injury, which makes it difficult to effectively implement preventive measures. Secondly, there is a general shortage of nursing assistant in nursing homes. Nursing assistant have high work intensity and limited energy, and cannot provide meticulous care for every elderly person. Especially in the face of some special situations, they are more likely to neglect, which increases the risk of pressure injury. In addition, the facilities and equipment of some nursing homes have certain defects, such as mattresses that are not suitable for the physical condition of the elderly, and the shortage of beds that prevent the elderly from getting enough rest, which have hindered the prevention of pressure injury. We need to further strengthen our supervision of nursing homes to ensure that various preventive measures can be truly implemented to avoid regulatory loopholes. At the same time, the public's awareness and attention to pressure injury prevention also need to be improved. Through extensive publicity and education, a good atmosphere of common concern and support for pressure injury prevention should be formed throughout the society, so that nursing homes and relevant personnel can pay more attention to this issue. In short, to solve the difficulties and challenges of pressure injury prevention and management in nursing homes, we need to work together and take practical and effective measures to provide a safer and more comfortable nursing environment for the elderly.

**Interviewer:**

What specific abilities of nursing assistant have you observed to be most important for effective PIPM? what specific competencies do you observe in nursing assistants that contribute most to effective PIPM?

**Interviewee:**

When observing the effective performance of nursing assistants in the prevention and management of pressure injury, I found that the following specific competencies played a vital role. First of all, solid professional knowledge and superb nursing skills are the basic guarantee. They should have a deep understanding of the causes and development process of pressure injury, as well as various effective prevention methods and nursing skills. For example, they should know the role of different types of mattresses in preventing pressure injury, and master the correct turning skills and skin care methods. Only with these professional knowledge and skills can we accurately judge the physical condition of the elderly in actual work, take scientific and reasonable preventive measures in time, and effectively reduce the risk of pressure injury. In addition, a high sense of responsibility and patience are the core elements. Nursing assistants need to stay focused at all times and pay attention to the situation of each elderly person. Whether it is diet, sleep or daily activities, they must take meticulous care, patiently listen to the needs and demands of the elderly, and provide care and help in time. In terms of preventing pressure injury, they must be meticulous, carefully check every part where pressure injury may occur, and not ignore any small changes to ensure that the elderly are always in good condition.

**Interviewer:**

Okay, are there other skills needed?

**Interviewee:**

The professional ethics and code of conduct of nursing assistants in nursing homes are important guarantees for ensuring service quality and the welfare of the elderly. According to the <Regulations on the Management of Nursing Homes>, nursing homes and their staff should protect the personal rights, property rights, and other legal rights of the elderly in accordance with the law. In addition, good communication skills are an important link. They need to communicate actively and effectively with the elderly and their families, understand the elderly's living habits, health conditions and psychological states, so as to better provide personalized nursing services. At the same time, they should also promptly feedback the elderly's situation to the family members, let them know the elderly's living conditions in the nursing home, and enhance the family members' trust and support for the nursing home. In addition, nursing assistant also need good communication and cooperation, and jointly discuss and formulate plans for the prevention and management of pressure injury to ensure that all work can be carried out smoothly. Teamwork spirit is even more indispensable. The prevention and management of pressure injury in nursing homes is a systematic project that requires mutual cooperation and support among nursing assistant. They should share work tasks, exchange experiences and insights, form a good cooperative atmosphere in the team, and work together for the health of the elderly. In short, nursing assistant with these abilities can play an important role in the prevention and management of pressure injury, provide better, more comprehensive and more intimate nursing services for the elderly in nursing homes, and allow them to enjoy their old age in nursing homes.

**Interviewer:**

what is your perspective on the importance of nursing assistants' attitudes or values towards PI prevention?

**Interviewee:**

From the perspective of our Civil Affairs Bureau, the positive attitude and correct values of nursing assistants are key factors to ensure the effective implementation of pressure injury prevention. A nursing assistant with a high sense of responsibility and professionalism will regard the prevention of pressure injury as his or her primary task, take every nursing link seriously, and will not slack off because of the tediousness of the work. They will take the health and safety of the elderly as the starting point and provide high-quality nursing services to the elderly wholeheartedly. At the same time, the care and respect of nursing assistants for the elderly are also crucial. They should regard the elderly as their own relatives, feel the needs and pain of the elderly with their hearts, and give them enough care and attention. This kind of care can not only make the elderly feel warm and comforted, but also help to establish a good nursing assistant-patient relationship, improve the compliance and cooperation of the elderly, and thus better prevent the occurrence of pressure injury.

In addition, the teamwork awareness and dedication of nursing assistants can also reflect their emphasis on the prevention of pressure injury. In nursing institutions, the prevention of pressure injury is not a task that can be completed by one person, and it requires mutual cooperation and support among nursing assistants. A nursing assistant with a good sense of teamwork will actively cooperate with other colleagues to contribute to the prevention of pressure injury. The spirit of dedication makes them willing to spend more effort and time for the health of the elderly.

**Interviewer:**

Okay, what personality traits do you think drive nursing assistants to be proactive in PIPM?

**Interviewee:**

In my opinion, the following personality traits play an important role in promoting the proactive prevention and management of pressure injury by nursing assistant. The first is carefulness, which is a very critical point. Careful nursing assistant can keenly perceive the subtle changes in the elderly's body, discover the hidden dangers that may cause pressure injury in time, and take corresponding preventive measures. They will not ignore any small details, but take every link seriously to ensure the safety and comfort of the elderly. The second is patience. Pressure injury prevention is a long-term job that requires nursing assistant to have enough patience. They can take care of the elderly tirelessly, patiently answer the elderly's questions, and give them care and support. Patience is also reflected in their serious attitude towards work, and they will not give up easily because of difficulties.

**Interviewer:**

Anything else?

**Interviewee:**

There is also a strong sense of responsibility, which is the internal motivation for nursing assistant to take positive actions. They are fully aware of their responsibilities, put the health of the elderly first, actively learn and master the knowledge and skills of pressure injury prevention, and strive to provide the best care services for the elderly. In addition, it is also important to have good communication skills. They can communicate effectively with the elderly and their families, understand the needs and situations of the elderly in a timely manner, and convey the importance of pressure injury prevention to them, and win their understanding and support. At the same time, perseverance is also indispensable. In the face of various difficulties and challenges, they can work tirelessly, not be intimidated by difficulties, and always maintain their enthusiasm and enthusiasm for pressure injury prevention.

**Interviewer:**

How do institutional culture and policies influence nursing assistants' motivation to perform PIPM?

**Interviewee:**

Institutional culture and policies greatly affect the enthusiasm of nursing assistant in preventing and managing pressure injury. A positive and healthy institutional culture can create an atmosphere of caring for the elderly and focusing on the quality of care, which can subtly influence nursing assistant in such an environment, stimulate their inner sense of mission and responsibility, and thus be more motivated to do a good job in preventing and managing pressure injury. This culture can also promote cooperation and communication among nursing assistant, so that everyone can learn from each other, encourage each other, and work together to improve the prevention effect of pressure injury. Perfect policies play a role in clarifying guidance and regulating behavior. Reasonable policies will clearly define the responsibilities and requirements of nursing assistant in the prevention and management of pressure injury, so that they know clearly what they should do and how to do it. At the same time, the reward mechanism for excellent performance in the policy can also motivate nursing assistant to actively improve their abilities and perform their duties more conscientiously. Policies can also protect the rights and interests of nursing assistant, make them feel respected and valued, and thus devote themselves to their work more enthusiastically. In addition, the consistency of institutional culture and policies is also very important. If the two echo and support each other, they can form a strong synergy to promote the continuous progress of nursing assistant in the prevention and management of pressure injury. On the contrary, if there is a disconnect or contradiction between culture and policy, it may make nursing assistant feel confused and lost, affecting their enthusiasm and work effectiveness. The assessment mechanism is the key to ensure that nursing assistants provide high-quality nursing services. Through regular and systematic assessment, we can ensure that the prevention and management measures for PI are effectively implemented. The assessment results help us identify the training needs of nursing assistants in the prevention and management of PI, so as to provide targeted education and training. In short, the Civil Affairs Bureau needs to pay attention to the construction of institutional culture and the formulation and improvement of policies, and provide nursing assistant with a good working environment and incentive mechanism to enhance their enthusiasm and work effectiveness in the prevention and management of pressure injury.

**Interviewer:**

What motives would further empower nursing assistants to perform PIPM effectively?

**Interviewee:**

First, the recognition and reward mechanism is very important. When the efforts and contributions of nursing assistant are fully recognized and affirmed, they will have a greater sense of accomplishment and more motivation to continue to do this work well. Some special rewards can be set up, such as commending outstanding nursing assistant and giving material rewards, which can stimulate their enthusiasm and competitive awareness. Secondly, providing continuous training and learning opportunities is also key. Let nursing assistant continuously improve their professional knowledge and skills, and understand the latest methods and techniques for the prevention and management of pressure injury. They will have more confidence and ability to cope with various challenges in their work, so as to prevent and manage pressure injury more effectively. Furthermore, creating a good working environment and team atmosphere can also play a positive role in promoting the work. Let the nursing assistant work in a harmonious and supportive environment, they will feel the power of the team and be more willing to work with their colleagues to serve the health of the elderly. In addition, fully communicating and exchanging with nursing assistant, understanding their needs and ideas, and involving them in the formulation of relevant policies and measures can also enhance their sense of belonging and responsibility, and encourage them to be more actively involved in the prevention and management of pressure injury.

**Interviewer:**

Okay, thank you very much for your answer. The next question is about pressure injury training. Could you please talk about the current situation of your institution's training on the prevention and management of pressure injury for nursing assistant?

**Interviewee:**

In our institution, we attach great importance to the prevention and management training of pressure injury for nursing assistant. We have formulated a comprehensive training plan and regularly organize nursing assistant to participate in professional training courses. These courses cover theoretical knowledge, evaluation methods, nursing skills, and the latest research progress of pressure injury prevention. In the training process, we focus on the combination of theory and practice, not only allowing nursing assistant to master a solid theoretical foundation, but also through practical operations and case analysis, so that they can apply what they have learned to actual work.

At the same time, we will also invite experts in related fields to give lectures to ensure the quality and professionalism of the training. In addition, we will evaluate the training effect and keep abreast of the nursing assistant' mastery of the knowledge so as to adjust and optimize the training content and methods. In addition, we also encourage nursing assistant to communicate and share with each other so that they can learn from each other and jointly improve the level of pressure injury prevention and management. Although we have made a lot of efforts in training, we also realize that there is still room for further improvement. We will continue to improve the training system to better meet the needs of nursing assistant, improve their professional quality and work ability, and provide better care services for the elderly in nursing homes.

**Interviewer:**

Okay, in response to the current training situation you just mentioned, can you talk about the needs and suggestions for pressure injury training?

**Interviewee:**

As far as pressure injury training is concerned, we do find some needs and areas for improvement. First, the training content needs to be more in-depth and comprehensive. In addition to basic pressure injury prevention knowledge and nursing skills, it should also include the latest research results, advanced nursing concepts and methods, so that nursing assistant can constantly update their knowledge and better deal with various situations in actual work. Secondly, the training methods need to be more diversified. In addition to traditional classroom lectures, online training, practical operations, case analysis, group discussions and other links can also be added to enable nursing assistant to understand and master knowledge more deeply and improve their practical operation capabilities. In addition, the frequency of training also needs to be appropriately increased. Pressure injury prevention is a long-term and important task. Nursing assistant need to continuously strengthen and consolidate relevant knowledge and skills, so regular training is very necessary. At the same time, we recommend strengthening interaction and communication with nursing assistant during training, understanding their actual needs and difficulties, and conducting targeted training and guidance. In addition, a post-training tracking and evaluation mechanism can be established to timely understand the training effect of nursing assistant so as to continuously improve the training work.

**Interviewer:**

Okay. Is there anything else to add?

**Interviewee:**

No more

**Interviewer:**

Okay, thank you very much for participating in this interview. From the current situation of nursing homes, the current situation of pressure injury training, the pressure injury capacity requirements of nursing assistant, the pressure injury training needs and suggestions, if you have anything to add later, please feel free to contact me, thank you very much!
