# Supplementary material for: Exploring nursing assistants’ competencies in pressure injury prevention and management in nursing homes: a qualitative study using the iceberg model
Source: BMC Nurs. 2025 Mar 27;24:333. doi: 10.1186/s12912-025-02911-6 (PMC11948734; doi:10.1186/s12912-025-02911-6)
Supplement: Supplementary file 1 — Supplementary Material 1 [file 12912_2025_2911_MOESM1_ESM.zip › Nurisng home nurse indepth interview transcript.docx]

**Nursing home nurse in-depth interview transcript**

**Interviewer:**

Hello, Mrs ***. I am from ***. My name is ***. We are currently doing a study to gain an in-depth understanding of the nursing assistant's pressure injury prevention and management capabilities, training status, training needs and training suggestions from the perspective of nursing home nurse, so as to provide a reference for nursing homes to formulate feasible training plans and carry out pressure injury management. During this interview, we need to record the entire interview process, but all information will be kept confidential, personal information will not be disclosed, and the interview content will only be used for research. Are you willing to participate in this interview?

**Interviewee:**

OK, no problem

**Interviewer:**

Thank you very much. Here is an informed consent form. Please sign it.

**Interviewee:**

OK

**Interviewer:**

First, please introduce your professional background and work experience, especially the experience related to the prevention and management of pressure injury.

**Interviewee:**

I am a nurse working in a nursing home. I graduated from Nanjing Health College and have been working in the nursing industry for 12 years. At work, I have accumulated rich experience, especially in the prevention and management of pressure injury. I carefully observe the skin condition of the elderly every day, and pay special attention to those parts that are prone to pressure injury, such as the sacrum and heels. I will regularly turn the elderly over and massage them to keep their skin clean and dry. At the same time, I will also choose suitable mattresses and care products for them according to their physical conditions and needs to reduce the pressure on their skin. I still remember that once an elderly person showed signs of mild pressure injury, I took timely measures to strengthen care, and worked with other colleagues to develop a special care plan. After a period of careful care, the elderly's pressure injury gradually improved, which made me feel very fulfilled. These experiences have made me more aware of the importance of pressure injury prevention and management, and have also made me continue to work hard to improve my professional ability and provide better care services for the elderly in nursing homes.

**Interviewer:**

Have you ever participated in or provided training on pressure injury prevention and management? If yes, what types of training or courses have you provided?

**Interviewee:**

I have indeed participated in and provided training on the prevention and management of pressure injury. The types of training I have provided include theoretical knowledge explanations, such as the causes, stages, and preventive measures of pressure injury; practical operation demonstrations, such as how to properly turn the elderly over, change body positions, and use pressure relief devices; and sharing some cases so that nursing assistant can better understand and apply the methods of pressure injury prevention and management through actual cases. In addition, we will also emphasize the important role of nursing assistant' sense of responsibility and carefulness in the prevention of pressure injury, so that everyone can always be vigilant and provide more thoughtful care for the elderly.

**Interviewer:**

What role do you think nursing assistant in nursing homes play in the prevention and management of pressure injury?

**Interviewee:**

Nursing assistants in nursing homes play a vital role in the prevention and management of pressure injury. They are the people who have the most direct and frequent contact with the elderly and can observe changes in the elderly's skin condition in a timely manner. Their careful and meticulous care, such as regularly assisting the elderly to turn over and keeping their skin clean and dry, can effectively prevent the occurrence of pressure injury. Moreover, they can provide personalized care plans according to the specific conditions of the elderly, which is very important for the prevention and management of pressure injury. In addition, the patience and care of the nursing assistant can also make the elderly feel comfortable and at ease, which is of great benefit to their physical and mental health. In short, nursing assistant are an indispensable force in the prevention and management of pressure injury.

**Interviewer:**

What specific competencies do you observe in nursing assistants that contribute most to effective PIPM?

**Interview subjects:**

Nursing assistants need to know what PI is and why it is an important indicator of quality control in nursing home management. Therefore, they need to understand the current epidemiological characteristics of PI, the prevalence of PI in hospitals, communities, and nursing homes, and the prevalence of PI in the elderly population. They need to know this data to better implement PIPM. They must be very skilled in a series of skills such as turning over, massaging, and using pressure relief equipment. Not only do they need to know how to operate correctly, but they must also make flexible adjustments based on the specific conditions of the elderly to ensure that the elderly's body is always in a comfortable state and effectively reduce the pressure on the skin. In addition, they also need to understand the characteristics and treatment methods of different types of pressure injury, and be able to quickly make correct judgments and handle problems when they are found. Based on the patient's specific circumstances and risk factors, nursing assistants can develop personalized care plans. Nursing assistants can convey this basic knowledge to patients and families to help them understand the importance of preventing PI. At the same time, they must master various measures to prevent pressure injury, such as keeping the skin clean and dry, and adjusting the diet reasonably. They also need to have extremely keen and meticulous observation skills, and be able to accurately detect any subtle changes in the elderly's skin, such as subtle changes in skin color, whether there is redness and swelling, whether there are nodules, etc. This is an important prerequisite for timely discovering potential problems and taking corresponding measures. Good communication skills are also essential. They must be able to communicate deeply, smoothly, and effectively with the elderly and their families, and fully understand various information such as the elderly's physical condition, living habits, diet, and special needs. It is very important for nursing assistants to understand the causes and characteristics of PI in the elderly. This can help them realize that the elderly are a high-risk group for PI and improve their awareness of prevention. Timely identification and treatment of PI can reduce the occurrence of complications such as infection and reduce the health risks of patients. At the same time, a high sense of responsibility is a solid foundation for them to do a good job in the prevention and management of pressure injury. They should treat every nursing task with a serious and responsible attitude, not despise any details, not be careless or perfunctory, and ensure that the elderly receive meticulous care. They also need to have a spirit of continuous learning, actively update their knowledge in the prevention and management of pressure injury, keep abreast of the latest nursing concepts and methods, and continuously improve their professional quality. They should be able to combine theoretical knowledge with practical operations, and continuously accumulate experience in practice to better deal with various complex situations. Only with these abilities can nursing assistants play the greatest role in the prevention and management of pressure injury, provide better and more effective nursing services for the elderly, and allow the elderly to enjoy more intimate and professional care in nursing institutions.

**Interviewer:**

What is your perspective on the importance of nursing assistants' attitudes or values towards PI prevention?

**Interviewee:**

Their positive attitude can prompt them to treat every nursing work more seriously and carefully. If the nursing assistants attach great importance to and have a correct understanding of the prevention of pressure injury, they will take the initiative to learn relevant knowledge and skills, and constantly improve their abilities, so as to better prevent the occurrence of pressure injury. They will regard the prevention of pressure injury as their responsibility and mission, and do their best to take care of every elderly person. And the values are reflected in their care and respect for the elderly. A caring and patient nursing assistant will take the needs of the elderly as the starting point, pay attention to the physical and mental health of the elderly, and take care of every elderly person with heart. They will understand the pain and discomfort of the elderly, try their best to provide the elderly with a comfortable nursing environment and care, and let the elderly feel warmth and care. This positive attitude and correct values can greatly improve the effect of pressure injury prevention and escort the health and happiness of the elderly

**Interviewer:**

What personality traits do you think drive nursing assistants to be proactive in pressure injury prevention and management? What personality traits do you think drive nursing assistants to be proactive in PIPM?

**Interviewee:**

First of all, it is a sense of responsibility. They will take the health of every elderly person to heart, take every detail of care seriously, and will not perfunctorily do things because of the tediousness of things. Patience is also needed. Pressure injury prevention is a long-term process. They need to have enough patience to repeat various nursing operations and pay attention to the subtle changes of the elderly. Carefulness is also very important. They can keenly detect any abnormalities in the elderly's skin and take timely measures to avoid the deterioration of the problem. Love is also indispensable. They truly care for the elderly, are willing to pay for the elderly, and strive to make the elderly live comfortably and healthily. In addition, the trait of being studious can also enable them to continuously learn new knowledge and skills, improve their ability in pressure injury prevention and management, and better serve the elderly.

**Interviewer:**

How do institutional culture and policies influence nursing assistants' motivation to perform PIPM?

The culture and policies of the institution have a great influence on the motivation of nursing assistants in performing pressure injury prevention and management. A positive institutional culture that focuses on nursing quality will make nursing assistants feel the importance and value of their work, thereby stimulating their sense of responsibility and mission, and more actively doing a good job in pressure injury prevention and management. In such a cultural atmosphere, everyone will learn from each other, encourage each other, and form a good working atmosphere. A sound policy system can provide clear guidance and norms for nursing assistants, so that they know what to do and to what extent. A reasonable incentive mechanism and assessment system can also mobilize their enthusiasm and make them more motivated to work hard to prevent and manage pressure injury. If the organization can give nursing assistants enough support and recognition, they will be more willing to devote themselves to this work and contribute their own strength to the health of the elderly.

**Interviewer:**

What motives would further empower nursing assistants to perform PIPM effectively?

**Interviewee:**

First, let them understand the importance of this work to the health of the elderly, which can inspire their sense of professional honor and mission and make them more motivated to do a good job. Give them enough training and learning opportunities to improve their professional ability and make them confident to deal with various situations, which can also motivate them to do a better job in the prevention and management of pressure injury. There is also a reasonable reward mechanism to give recognition and rewards to outstanding nursing assistant, which can stimulate their sense of competition and make them work harder to do their best. In addition, creating a good team atmosphere where everyone supports and cooperates with each other can also make them feel more belonging and more willing to work for the team's goals. At the same time, paying attention to their personal needs and emotions, making them feel respected and cared for, can also improve their work enthusiasm.

**Interviewer:**

Okay, thank you very much for your answer. The next question is about pressure injury training. Could you please talk about the current situation of your institution's training on the prevention and management of pressure injury for nursing assistant?

**Interviewee:**

We regularly arrange special training courses, which cover a wide range of content. There are not only detailed theoretical explanations, such as the causes of pressure injury, manifestations at different stages, and how to effectively prevent them, but also practical demonstrations, including how to correctly turn over and massage the elderly, and how to choose and use appropriate pressure relief equipment. Through these trainings, nursing assistant can fully and systematically master the relevant knowledge and skills of pressure injury prevention and management. Moreover, we will also analyze specific cases to allow nursing assistant to more intuitively understand how to deal with different situations, how to promptly discover potential problems and take effective measures. During the training process, we will repeatedly emphasize the responsibility and carefulness of nursing assistant, so that they can truly understand the importance of pressure injury prevention for the health of the elderly, thereby enhancing their sense of responsibility and mission.

However, I personally believe that our training can be further targeted and effective. For example, we can formulate more targeted training plans based on the different work experience and skill levels of nursing assistant; we can also add some interactive links to give nursing assistant more opportunities to conduct practical operations and exchanges and discussions to better consolidate the knowledge they have learned. At the same time, we can also introduce some advanced technologies and methods to enable nursing assistant to keep pace with the times and continuously improve their professional quality.

**Interviewer:**

Okay, could you please talk about the training needs and suggestions for pressure injury?

**Interviewee:**

As far as pressure injury training is concerned, I think more in-depth and comprehensive theoretical knowledge training is needed first. Nursing assistant should have a clearer understanding of the formation mechanism of pressure injury, the characteristics of different stages, and various prevention and treatment methods. At the same time, practical training is also very important, including how to properly turn the elderly over, clean, and care for them. These require repeated practice to ensure that nursing assistant can master them proficiently. In addition, it is recommended to add some case analysis and discussion links so that nursing assistant can better understand and apply the knowledge they have learned in combination with actual conditions. In addition, the frequency of training can be appropriately increased to continuously strengthen the memory and skills of nursing assistant. And some advanced technologies and equipment can be introduced to let nursing assistant understand the latest methods of pressure injury prevention and management. Finally, I hope there will be more practical opportunities so that nursing assistant can continuously accumulate experience in actual work and improve their ability to deal with various situations.

**Interviewer:**

Okay, you just mentioned the continuous training of knowledge and technology, training form suggestions, practical training and multidisciplinary cooperation. Is there anything else you need to add?

**Interviewee:**

No

**Interviewer:**

Okay. Thank you very much for participating in this interview. You have given us some information about the current situation of nursing homes, the status of pressure injury training, the pressure injury capacity requirements of nursing assistant, and the pressure injury training needs and suggestions. If you have anything else to add later, please feel free to contact me. Thank you very much!
