# Supplementary material for: Exploring nursing assistants’ competencies in pressure injury prevention and management in nursing homes: a qualitative study using the iceberg model
Source: BMC Nurs. 2025 Mar 27;24:333. doi: 10.1186/s12912-025-02911-6 (PMC11948734; doi:10.1186/s12912-025-02911-6)
Supplement: Supplementary file 1 — Supplementary Material 1 [file 12912_2025_2911_MOESM1_ESM.zip › Nursing home administrator 1 indepth interview transcript.docx]

**Nursing home administrator 1 in-depth interview transcript**

**Interviewer:**

Hello, Mrs ***. I am from ***. My name is ***. We are currently doing a study to gain an in-depth understanding of the nursing assistant's pressure injury prevention and management capabilities, training status, training needs and training suggestions from the perspective of nursing home administrators, so as to provide a reference for nursing homes to formulate feasible training plans and carry out pressure injury management. During this interview, we need to record the entire interview process, but all information will be kept confidential, personal information will not be disclosed, and the interview content will only be used for research. Are you willing to participate in this interview?

**Interviewee:**

Okay

**Interviewer:**

First, I would like to ask about your professional background. Please introduce your work organization, including the scale, type, and service objects of this organization.

**Interviewee:**

We are a public institution under the Civil Affairs Bureau, that is, Huai'an Social Welfare Institute. We used to have 600 beds here, but because the building behind is under maintenance, we now have 260-240 beds, and have made some adjustments. The objects of our service are some elderly people in society, including some "three-no" people in the city. For the "three-no" people in the city, it is now called special supply, and the number of support personnel should be about 53, and the number of elderly people here is about 138. The proportion of disabled elderly people is about 15%, and the number of completely bedridden elderly people is about 15, and the number of nursing assistants is 34.

**Interviewer:**

Okay, then what difficulties or challenges do you think there are in the prevention and management of pressure injury in nursing homes?

**Interviewee:**

The budget of nursing homes is limited, and they need to balance between personnel, equipment and daily operations. For example, purchasing high-quality pressure-relieving mattresses and cushions requires a large investment, which may affect the budget for other aspects. In addition, the turnover of nursing assistant is high, and new employees need to be trained in the prevention and management of pressure injury. However, training resources are limited, and frequent training may affect daily operations. Nursing assistant shortage is a common problem, especially in institutions that require 24-hour care. Insufficient staff may result in an inability to frequently help the elderly turn over, which is a key measure to prevent pressure injury. For example, our institution once faced a shortage of nursing assistant, which resulted in an inability to provide enough turning times for each elderly person. To solve this problem, we introduced an automatic turning bed. Although the initial investment was large, in the long run, it not only reduced the burden on nursing assistant, but also significantly reduced the incidence of pressure injury. At the same time, some elderly people may be unwilling to cooperate with turning over or using pressure-relieving equipment due to pain, cognitive impairment or other health problems. Family members may not fully understand the importance of pressure injury prevention or have misunderstandings about nursing measures, which may affect the implementation of prevention measures. We once encountered a case where the family members of an elderly person did not understand why they needed to turn over frequently, and they thought it disturbed the elderly person's rest. We communicated with the family members, explained the risks of pressure injury and the importance of turning, and finally gained their understanding and support. Regulations and standards that need to be followed are constantly changing, and managers need to ensure that the policies and procedures of the institution meet the latest requirements. As new technologies and methods emerge, managers need to evaluate and adopt these new technologies to improve the effectiveness of pressure injury prevention. Accurately recording the skin condition and care measures of the elderly is necessary, but it may become cumbersome due to the large workload.

**Interviewer:**

What specific competencies do you observe in nursing assistants that contribute most to effective pressure injury prevention and management?

**Interviewee:**

I think nursing assistants need to have solid professional knowledge in implementing pressure injury prevention and management, including the causes, stages, and prevention measures of pressure injury. For example, we have a nursing assistant who can accurately identify the early signs of pressure injury and take timely measures, thus avoiding the occurrence of multiple pressure injury. Nursing assistants need to have a detailed observation of the skin condition of the elderly and be able to identify subtle changes in skin condition in time, which helps to detect the risk of pressure injury at an early stage. Good communication skills help nursing assistants build trust with the elderly and their families, convey the importance of pressure injury prevention, and explain the required care measures.

**Interviewer:**

Okay, are there any other competencies needed?

**Interviewee:**

Nursing assistants also need to be able to perform accurate physical assessments, including assessments of skin condition and mobility, to determine the risk of pressure injury. The ability to work effectively in a multidisciplinary team is essential to implement a comprehensive pressure injury prevention program. Patience and empathy for the elderly help nursing assistants provide personalized care and help the elderly adapt to nursing measures. As nursing assistants, first of all, they need to know what the professional norms of nursing assistants are, basic professional ethics knowledge, professional qualities, and professional skills. In particular, they need to understand the requirements of nursing homes for PI quality management and the relevant reporting process. Strict compliance with the institution's nursing procedures and industry standards is essential for preventing pressure injury. Only by understanding them can they strictly abide by the norms and requirements of the nursing assistants industry, not be lazy or perfunctory, and always maintain professional ethics. Accurately recording the elderly's care process and skin condition changes is essential for monitoring the risk of pressure injury and evaluating the effectiveness of preventive measures.

Basic knowledge of laws and regulations, especially those related to nursing homes, such as the <Regulations on the Administration of Nursing Homes>, Service Standards for Prevention of Pressure Injuries in Nursing Homes". and the <Law on the Protection of the Rights and Interests of the Elderly>. In terms of professional ethics, nursing home nursing assistants should abide by the following standards, including respecting and protecting the rights and interests of the elderly, not discriminating against or insulting the elderly, following the autonomous wishes of the elderly, and protecting the privacy of the elderly.

**Interviewer:**

what is your perspective on the importance of nursing assistants' attitudes or values towards pressure injury prevention?

**Interviewee:**

As a manager of a nursing home, I believe that nursing assistants' attitudes and values are essential for pressure injury prevention. Nursing assistants' professional commitment is the cornerstone to ensure that they adhere to nursing standards and protocols. They must recognize the role they play in the care of the elderly and be committed to providing the highest quality care. Professional commitment is also reflected in the emphasis on continuing education and training. For example, we encourage nursing assistants to participate in seminars and workshops on pressure injury prevention to keep their knowledge updated.

Empathy enables nursing assistants to better understand the needs and feelings of the elderly, thereby providing more humane care. Nursing assistants with empathy are more likely to take extra steps to ensure the comfort and dignity of the elderly, for example, being more careful when turning over to avoid unnecessary discomfort.

A strong sense of responsibility prompts nursing assistants to take the initiative to ensure that pressure injury prevention measures are implemented. This means that nursing assistants need to report potential problems in a timely manner when they find them, and actively participate in solving problems instead of waiting for others to guide them.

**Interviewer:**

Okay, you just mentioned the quality characteristics or professional qualities that nursing assistants need, such as professional commitment or professional identity, empathy, and responsibility. Are there any other qualities that need to be added?

**Interviewee:**

Well, another important quality is that pressure injury prevention requires patience, because some nursing measures may need to be performed repeatedly. Patience also means that nursing assistants can remain calm in the face of challenges, for example, when the elderly do not understand or resist the nursing procedures, nursing assistants need to patiently explain and comfort them.

**Interviewer:**

What personality traits do you think drive nursing assistants to be proactive in pressure injury prevention and management?

**Interviewee:**

The nursing assistants need to have a meticulous personality and conduct regular and thorough inspections of the elderly's skin. For example, the nursing assistants in our institution use a skin inspection form to record any abnormalities at every shift change, and this habit helps to detect signs of pressure injury early.

In addition, nursing assistants should be encouraging and motivate the elderly to take care of themselves as much as possible within their physical abilities. For example, we encourage nursing assistants to help the elderly with appropriate activities such as walking or doing light stretching, which can help improve blood circulation and reduce the risk of pressure injury.

**Interviewer:**

Well, these are the personality traits you think nursing assistants need, right?

**Interviewee:**

Yes, in addition, I think nursing assistants also need to show a tolerant personality and not blame the elderly for their mistakes or misunderstandings. For example, if the elderly fail to follow the turning schedule, the nursing assistants should patiently re-explain the importance of turning instead of blaming.

In addition, I think nursing assistants should also be proactive and actively identify and meet the needs of the elderly. For example, the nursing assistants in our institution will proactively ask the elderly if they need to adjust the mattress or cushion to ensure their comfort.

**Interviewer:**

How do institutional culture and policies influence nursing assistants' motivation to perform pressure injury prevention and management?

**Interviewee:**

A fair and transparent assessment and evaluation mechanism can motivate nursing assistants to participate more actively in pressure injury prevention and management. Our institution has implemented regular skin checks and pressure injury risk assessment processes, and these assessment results are used as part of the nursing assistants' job performance. This mechanism encourages nursing assistants to conscientiously implement the pressure injury prevention and management process because they know that this will directly affect their job evaluation.

Providing continuing education opportunities can not only improve nursing assistants' professional knowledge, but also increase their motivation to perform pressure injury prevention and management. Our institution encourages nursing assistants to participate in relevant continuing education courses and seminars to earn credits. These courses usually cover the latest knowledge of pressure injury prevention and management, helping nursing assistants improve their skills. At the same time, participating in these courses can also gain recognition in their careers.

Reward mechanism is an effective means to improve the motivation of nursing assistants. Our institution has a reward program to give bonuses, recognition or extra leave to nursing assistants who perform well in pressure injury prevention and management. This positive incentive significantly improves the enthusiasm of nursing assistants and makes them pay more attention to the prevention and management of pressure injury.

In addition, a patient-centered institutional culture can stimulate the intrinsic motivation of nursing assistants. Our institution emphasizes the needs and well-being of the elderly, and this culture makes nursing assistants naturally pay more attention to the prevention and management of pressure injury. When nursing assistants feel that their work is valued and that they can provide substantial help to the elderly, their enthusiasm will be significantly improved.

**Interviewer:**

What motives would further empower nursing assistants to perform pressure injury prevention and management effectively?

**Interviewee:**

Nursing assistants often want to improve their professional abilities, which not only helps them do their jobs better, but also increases their sense of professional accomplishment. Our organization provides regular training and learning opportunities to encourage nursing assistants to learn new nursing techniques and knowledge. For example, we recently introduced a new pressure injury risk assessment tool and trained all nursing assistants on how to use it. This ability improvement makes nursing assistants more confident and efficient in performing pressure injury prevention and management.

Nursing assistants often have a desire for career advancement, and the effective implementation of pressure injury prevention and management can be a highlight of their career development. Our organization considers the performance of nursing assistants in pressure injury prevention and management when evaluating promotion opportunities. Those nursing assistants who can demonstrate significant achievements in pressure injury prevention and management will be given priority for promotion. This incentive of promotion opportunities encourages nursing assistants to focus more on improving their work performance.

In addition to career advancement, material rewards and public recognition are also important means of motivating nursing assistants. Our organization has a reward mechanism to give bonuses, extra vacations or other forms of rewards to nursing assistants who perform well in pressure injury prevention and management. In addition, we will publicly commend these nursing assistants at staff meetings as a sign of encouragement.

Regular performance feedback can help nursing assistants understand their work performance, as well as their achievements and areas for improvement in pressure injury prevention and management. Our institution has implemented a performance feedback system through which nursing assistants can receive regular feedback on their work performance. This feedback includes not only the implementation of pressure injury prevention and management, but also their overall contribution to the care of the elderly.

Through these motivational incentives, nursing assistants will be more proactive in implementing pressure injury prevention and management, thereby improving the quality of care for the entire nursing home. As managers, we are committed to creating a work environment that can inspire the potential of nursing assistants

**Interviewer:**

Okay, thank you very much for your answer. The next question is about pressure injury training. Could you please talk about the current status of pressure injury prevention and management training for nursing assistants in your institution?

**Interviewee:**

Our institution provides regular pressure injury prevention and management training courses for all nursing assistants. However, pressure injury training is integrated into the content of all training programs, such as dietary care, burn care, medication care, fall prevention care, etc. These courses include both online and on-site forms, covering the identification of pressure injury, risk assessment, preventive measures, wound care, etc. We ensure that every caregiver can master relevant nursing knowledge and skills

In addition to theoretical learning, we also attach importance to practical exercises. By simulating real situations, nursing assistants can practice skills such as turning over, skin examination and wound care on simulated people or each other to improve their practical ability.

**Interviewer:**

Okay, how is the assessment and evaluation implemented?

**Interviewee:**

After the training, we will conduct theoretical and practical assessments on nursing assistants. The assessment not only tests the learning outcomes of nursing assistants, but also helps them consolidate their knowledge. At the same time, we will also collect feedback from nursing assistants on the training so as to continuously improve the content and methods of training. We encourage nursing assistants to participate in continuing education to keep up with the latest nursing knowledge and technology. We provide learning resources and time support, and encourage nursing assistants to participate in seminars and workshops. We regularly hold case seminars to analyze and discuss successful cases and failure lessons in the prevention and management of pressure injury. Through case studies, nursing assistants can learn how to apply theoretical knowledge in actual situations and learn from the experience of others.

**Interviewer:**

Okay, in response to the current training situation you just mentioned, can you talk about the needs and suggestions for pressure injury training?

**Interviewee:**

As a manager of a nursing home, I recognize the importance of pressure injury training in improving the quality of care. I believe that all nursing assistants should receive basic training in the prevention and management of pressure injury. Institutions should ensure that all new employees receive basic training before taking up their posts and provide regular refresher training for existing employees. Experienced nursing assistants may need more advanced training to master more complex pressure injury management skills. Provide advanced training courses such as wound care workshops or advanced risk assessment techniques for experienced nursing assistants. As medical knowledge and technology continue to advance, continuing education is essential to keep nursing assistants updated. Establish a continuing education program and encourage nursing assistants to participate in relevant seminars, webinars and online courses. Theoretical knowledge needs to be strengthened through practical skills training. Improve the practical skills of nursing assistants through simulation training and actual operation drills. The prevention and management of pressure injury requires the cooperation of a multidisciplinary team. Organize multidisciplinary team training to promote collaboration and communication between nursing assistants from different professional backgrounds.

**Interviewer:**

Okay, you just mentioned the continuous training of knowledge and technology, training format recommendations, practical training and multidisciplinary cooperation. Is there anything else you need to add?

**Interviewee:**

Well, I think nursing assistants also need to understand the latest policies and procedures for pressure injury prevention and management. Regularly update training materials to reflect the latest clinical guidelines and institutional policies. Nursing assistants need to understand how to participate in and promote quality improvement projects. Provide quality improvement training to teach nursing assistants how to use data and feedback to improve nursing practices. Effective training requires feedback and evaluation mechanisms to ensure quality. Establish a training feedback and evaluation system to regularly collect feedback from nursing assistants on training and make improvements based on feedback.

By meeting these training needs and implementing the above suggestions, we can improve nursing assistants' ability in pressure injury prevention and management, thereby improving the quality of life and care satisfaction of the elderly. These are the needs and suggestions for now.

**Interviewer:**

Okay. Thank you very much for participating in this interview. You have given me the current situation of nursing homes, the current situation of pressure injury training, the pressure injury capacity needs of nursing assistants, the pressure injury training needs and suggestions. If you have anything to add later, please feel free to contact me. Thank you very much!
