# Supplementary material for: Exploring nursing assistants’ competencies in pressure injury prevention and management in nursing homes: a qualitative study using the iceberg model
Source: BMC Nurs. 2025 Mar 27;24:333. doi: 10.1186/s12912-025-02911-6 (PMC11948734; doi:10.1186/s12912-025-02911-6)
Supplement: Supplementary file 1 — Supplementary Material 1 [file 12912_2025_2911_MOESM1_ESM.zip › Nursing home administrator 2 indepth interview transcript.docx]

**Nursing home administrator 2 in-depth interview transcript**

**Interviewer:**

Hello, Mr ***. I am from ***. My name is ***. We are currently doing a study to gain an in-depth understanding of the 's pressure injury prevention and management capabilities, training status, training needs and training suggestions from the perspective of nursing home administrators, so as to provide a reference for nursing homes to formulate feasible training plans and carry out pressure injury management. During this interview, we need to record the entire interview process, but all information will be kept confidential, personal information will not be disclosed, and the interview content will only be used for research. Are you willing to participate in this interview?

**Interviewee:**

Okay, no problem

**Interviewer:**

Okay, thank you. First of all, let's learn about your background information. The first thing is, please introduce, um, your work organization, including the scale of this organization, this type, and the basic information of the service objects.

**Interviewee:**

The total number of beds designed for our nursing home is 360, and there are about 280 elderly people living there. The types of elderly people we admit range from self-care to disabled elderly people, a total of six levels. That is to say, there are all types of elderly people with full coverage of this service. Well, for these elderly people, under normal circumstances, there are three types of elderly people in our institution now. One type is our social operation, that is, family elderly people, those guardians and children send them here.

The other type is the government's centralized support. Well, there are about 50 elderly people in this trusteeship. Among the 50, there are nearly 10 people who are entrusted to us by the Huai'an Disabled Persons' Federation, that is, some disabled people under the age of 60. We now have three types of service objects. The proportion of disabled elderly people here is also relatively high, about 80 people, and there are about 20 elderly people who are completely bedridden.

**Interviewer:**

Okay, thank you. Just now you also mentioned that there are many elderly people. How many nursing assistants are there to take care of them?

**Interviewee:**

There are about 50 front-line nursing assistants now. If our medical staff and our nursing assistants are included, there should be less than 60 people in total. It can basically meet these care needs. It fully meets the national standards.

**Interviewer:**

Okay, thank you for your introduction. Next is the question about pressure injury. First of all, what difficulties or challenges do you think there are in the prevention and management of pressure injury in nursing homes?

**Interviewee:**

The overall educational background and professional background of practitioners in the nursing care industry are relatively complex, and some lack systematic training in pressure injury care knowledge. They may not have a deep understanding of the occurrence mechanism of pressure injury, risk assessment methods, and the correct treatment methods for pressure injury at different stages, which can easily lead to the inability to timely and accurately identify pressure injury risk factors in daily nursing work and take effective preventive measures. For example, some may not be able to correctly judge the pressure condition of the elderly's skin and do not know how to adjust the body position to reduce local pressure, thereby increasing the possibility of pressure injury. Training is difficult and the effect is difficult to guarantee. Due to the irregular working hours of , centralized training is often difficult. Moreover, the training methods and content may not be vivid and practical enough, and find it difficult to apply theoretical knowledge to actual work after training. In addition, with the high turnover rate of personnel in the industry, the number of newly recruited continues to increase, and continuous training is required, which puts great pressure on management. Nursing homes usually face the problem of shortage, and each needs to take care of a large number of elderly people. This makes them bear huge work pressure and heavy nursing tasks in their daily work, including life care, rehabilitation care, psychological care and other aspects. Under this high-intensity working state, may neglect to pay attention to the details of pressure injury prevention due to fatigue. For example, failure to turn the elderly over and check the skin condition on time increases the risk of pressure injury. Long hours of work can also easily cause to feel burnout, reduce their enthusiasm and sense of responsibility for work, and further affect the quality of pressure injury prevention and management.

**Interviewer:**

In addition to the challenges from the perspective of , are there any problems with the elderly?

**Interviewee:**

Yes. For example, most of the elderly living in nursing homes suffer from a variety of chronic diseases, such as diabetes, cardiovascular disease, and nervous system diseases. These diseases can affect the physical function and blood circulation of the elderly, leading to insufficient nutrition supply to the skin, decreased skin resistance, and more prone to pressure injury. For example, diabetic patients may have peripheral neuropathy and vascular lesions, which make the skin insensitive and reduce the ability to perceive pressure and damage. At the same time, poor blood circulation can also affect the healing ability of wounds. Some elderly people also have mobility problems, long-term bed rest or wheelchair use, and their parts of the body are under pressure for a long time, which increases the risk of pressure injury. Moreover, for these elderly people, special skills and equipment may be needed to adjust their body positions to ensure safety and comfort, but nursing homes may have insufficient resources or operational difficulties in this regard.

**Interviewer:**

Are there any difficulties in other institutions?

**Interviewee:**

Pressure injury prevention and management requires certain equipment and material support, such as pressure relief mattresses, air mattresses, and turning pillows. However, due to limited funds, some nursing homes may not be equipped with sufficient quantity and high quality of these equipment. Ordinary mattresses may not be able to effectively disperse body pressure, increasing the risk of pressure injury in the elderly. Even if some pressure relief equipment is equipped, its performance may decline due to long-term use and improper maintenance, and it may not play its due role. In terms of pressure injury care products, such as wound dressings and disinfection products, there may also be insufficient supply or substandard quality. Appropriate wound dressings are essential for the treatment and healing of pressure injury, but if nursing homes cannot provide a variety of dressings suitable for different wound conditions, it may affect the treatment effect of pressure injury and prolong the healing time.

The operating costs of nursing homes are high, including site rental, staff wages, equipment procurement, material consumption and other aspects. The fees charged are often restricted by the market and policies, resulting in relatively tight funds. In this case, the investment in pressure injury prevention and management will be subject to certain restrictions. For example, it is impossible to regularly invite professional pressure injury care experts to the institution for training and guidance, it is impossible to carry out relevant scientific research and quality improvement projects, and it is impossible to update and improve the equipment and environment for pressure injury prevention and management in a timely manner.

Limited funds may also affect the construction and renovation of facilities in nursing homes. If the room layout of the nursing home is unreasonable and the bed facilities are not suitable for the physical condition of the elderly, the risk of pressure injury may increase. However, due to the lack of funds for renovation, these problems may exist for a long time, bringing difficulties to the prevention and management of pressure injury.

Some nursing homes have loopholes and deficiencies in the pressure injury prevention and management system. For example, there is a lack of clear pressure injury risk assessment processes and standards, and may be subjective and arbitrary when evaluating the elderly, resulting in the inability to accurately identify high-risk elderly people, and thus fail to take targeted preventive measures in a timely manner.

The pressure injury reporting and handling process may also be insufficiently standardized. If the fails to report the pressure injury in time after it occurs, or the management fails to organize relevant personnel to analyze and deal with it in time after reporting, it will affect the treatment effect of the pressure injury, and it will be impossible to learn lessons and improve the prevention measures.

The quality assessment system for the prevention and management of pressure injury may also be imperfect, and it is impossible to effectively supervise and motivate the work of , resulting in some not paying enough attention to the prevention of pressure injury and failing to implement it in place.

Family members have different understandings and levels of attention to pressure injury. Some family members may have misunderstandings or high expectations about the prevention of pressure injury in nursing homes. They may think that pressure injury should not occur after the elderly move into nursing homes, and once they occur, they will be dissatisfied and question the institution, which puts a lot of pressure on the management and nursing work of the institution. When communicating with family members about the prevention measures for pressure injury and the nursing situation of the elderly, conflicts and disagreements may arise between the two parties due to improper communication methods or information asymmetry. For example, family members may not agree to use certain decompression equipment or perform special nursing operations for the elderly, believing that it will cause discomfort to the elderly, but these measures are actually intended to prevent the occurrence of pressure injury. In addition, if family members fail to correctly implement pressure ulcer prevention measures during the home care of the elderly after they are discharged from the hospital, it may also cause recurrence or aggravation of pressure ulcers.

**Interviewer:**

Anything else to add?

**Interviewee:**

No, the above are some of the main difficulties and challenges faced in the prevention and management of pressure injury in nursing homes. To solve these problems, nursing homes need to start from personnel training, equipment and material allocation, management system improvement, communication and collaboration, and take comprehensive measures to continuously improve the level of pressure injury prevention and management, and provide better quality and safer nursing services for the elderly.

**Interviewer:**

What specific competencies do you observe in nursing assistants that contribute most to effective pressure injury prevention and management?

**Interviewee:**

Excellent nursing assistants need to have keen insight and be able to accurately assess the risk of pressure injuries in each elderly person. They will carefully observe the physical condition of the elderly, including skin color, temperature, humidity, etc., and pay attention to factors such as the elderly's mobility, bed rest time, and nutritional status. They will also skillfully use various professional assessment tools and indicators, such as the Braden rating scale, to conduct a comprehensive and systematic assessment of the elderly, so as to accurately determine the potential risk level and formulate personalized prevention plans based on the assessment results. This meticulous risk assessment ability is the key first step in preventing pressure injuries.

Nursing assistants need to master skin care knowledge and skills. They are well aware of the importance of keeping the elderly's skin clean and dry. In daily care, nursing assistants will choose mild and non-irritating cleaning products to gently clean the elderly's skin and avoid excessive friction and irritation. At the same time, they will also choose appropriate skin care products such as lotion and vaseline according to the elderly's skin condition to keep the skin moist. In operations such as turning over and massaging, nursing assistants will master the correct techniques and strength to avoid damage to the skin. They also know which body positions are most effective in preventing pressure injuries, and will patiently assist the elderly to change their body positions in time to reduce local pressure on the body. In addition, nursing assistants will pay special attention to areas prone to pressure injuries, such as the sacrum and heels, and strengthen care and protection.

They must know how to change the body position for the elderly. Nursing assistants can flexibly adjust their body positions according to the specific conditions of the elderly. They understand that maintaining the same posture for a long time is one of the important causes of pressure injuries, so they will regularly turn the elderly over and change their positions. They are familiar with various methods and techniques of adjusting body positions, and can disperse the pressure of the body as much as possible without affecting the comfort of the elderly. For some elderly people with limited mobility, nursing assistants will also use auxiliary devices, such as air mattresses and turning pads, to improve body positions and reduce the risk of pressure injuries. This ability to adjust body positions in a timely manner according to actual conditions plays a vital role in preventing pressure injuries.

In terms of professional ethics, nursing home nursing assistants should abide by the following standards, including respecting and protecting the rights and interests of the elderly, not discriminating against or insulting the elderly, following the autonomous wishes of the elderly, and protecting the privacy of the elderly.

**Interviewer:**

Are there any other ability requirements?

**Interviewee:**

Another requirement is that they need to know how to provide nutritional support to the elderly. Nursing assistants understand the importance of nutrition to the health of the elderly, and also understand the close relationship between nutritional status and pressure injuries. They will pay attention to the diet of the elderly and ensure that the elderly take in enough nutrients such as protein, vitamins, minerals, etc. to enhance the resistance of the skin and promote wound healing. For some elderly people with dysphagia or eating disorders, nursing assistants will work with nutritionists to develop personalized nutrition plans and provide adequate nutritional support for the elderly through nasogastric feeding, intravenous nutrition, etc. This ability to prevent pressure injuries from the nutritional aspect reflects the nursing assistants' attention and attention to the overall health of the elderly.

Nursing assistants need to pay close attention to changes in the skin condition of the elderly, and through daily observation and inspection, promptly identify and record problems. They can accurately record the implementation of nursing measures, including the time and number of turning over, skin care methods, etc., so as to evaluate and adjust the nursing effect. This meticulous monitoring and recording ability provides a reliable basis for the prevention and management of pressure injuries, and also helps to promptly identify problems and take effective countermeasures.

**Interviewer:**

what is your perspective on the importance of nursing assistants' attitudes or values towards pressure injury prevention and management prevention?

**Interviewee:**

must have a strong sense of responsibility and professionalism, which is the key to ensuring the effective implementation of prevention work. They will regard the prevention of pressure injuries as their important responsibility, take every nursing link seriously, and do not ignore any details that may cause pressure injuries. This attitude reflects their high sense of responsibility for the health of the elderly.

Having a caring and empathetic attitude is the basis for to establish a good relationship with the elderly. They can put themselves in the shoes of the elderly, understand their pain and discomfort, and take preventive measures more proactively. This kind of care and empathy also makes the elderly feel warm and cared for, improving their cooperation and quality of life.

Prevention of pressure injuries is a field that is constantly developing and changing, and need to maintain a spirit of learning and enterprising. They are willing to constantly learn new knowledge and skills and update their concepts to better cope with various challenges in prevention work. This proactive attitude helps to improve the nursing level of the entire nursing home.

**Interviewer:**

Okay, you just mentioned responsibility, empathy, love, etc., is there anything else you need to add?

**Interviewee:**

Prevention of pressure injuries is not the job of a single nursing assistants, but requires the joint efforts of the entire team. Nursing assistants have a good sense of teamwork and can collaborate and support each other with other colleagues to jointly protect the health of the elderly. This atmosphere of teamwork is essential for the smooth implementation of prevention work. Prevention of pressure injuries is a field that is constantly developing and changing, and nursing assistants need to maintain a spirit of learning and progress. They are willing to constantly learn new knowledge and skills and update their concepts to better cope with various challenges in prevention work. This proactive attitude helps to improve the nursing level of the entire nursing home.

**Interviewer:**

What personality traits do you think drive nursing assistants to be proactive in pressure injury prevention and management?

**Interviewee:**

Nursing assistants need to have a strong sense of responsibility, which is one of the most critical personality traits. Nursing assistants with a strong sense of responsibility will regard the health of the elderly as their primary responsibility and devote themselves to prevention work. They will take every nursing operation seriously, from daily skin cleaning, turning over to body position adjustment, and strictly follow the specifications without any carelessness. Even when faced with some difficulties or challenges, they will remain firm in their beliefs, not retreat or give up, and always stick to the front line of preventing pressure injuries. Their sense of responsibility is not only responsible for their work, but also respect and protect the life and health of the elderly.

In addition, they must be careful and serious. Nursing assistants are like keen observers who can see every subtle change in the elderly's body. They will carefully observe the skin condition and limb activity of the elderly, and promptly discover potential risks. When performing nursing operations, they are even more meticulous, ensuring that every movement is gentle and accurate to avoid unnecessary harm to the elderly. They will also carefully record each nursing situation so that they can keep abreast of the health dynamics of the elderly at any time and provide a strong basis for preventing pressure injuries. This careful and serious attitude enables them to take preventive measures in their prevention work and minimize the risk of pressure injuries.

In addition, patience is also very important. Preventing pressure injuries is a long-term job that requires nursing assistants to have enough patience. Patient and calm nursing assistants can maintain a calm mind in their day-to-day work and are not troubled by tedious work. They will patiently turn over and massage the elderly, repeating these seemingly simple but very important actions over and over again. Even if they encounter some uncooperative elderly people, they can remain patient and use gentle words and patient explanations to win the trust and cooperation of the elderly. Their patience and calmness make the elderly feel at ease and comfortable, and also create a good atmosphere for preventing pressure injuries.

At the same time, nursing assistants who have the characteristics of caring for others are full of love and compassion for the elderly. They will truly care and take care of the elderly as their own relatives, pay attention to their joys and sorrows, and care about their life needs. Their care is not only reflected in words, but also in actions. They will use warm smiles and kind greetings to make the elderly feel the warmth of home. Their care makes the elderly no longer feel lonely and helpless when facing the risk of pressure injuries, but full of confidence and courage. This trait of caring for others is one of the most valuable qualities in the work of preventing pressure injuries.

**Interviewer:**

Okay, you just mentioned a lot of personality traits that nursing assistants have. Is there anything else you need to add?

**Interviewee:**

Teamwork is very important in nursing homes. Nursing assistants with a team spirit can cooperate and support each other with other colleagues to prevent and manage pressure injuries. They will understand the goals of the team and work together to achieve them. At work, they will take the initiative to communicate with colleagues, share their experiences and insights, learn from each other, and promote each other. They will also play their own advantages in the team and contribute their own strengths to the development of the team. Their teamwork spirit has made the entire nursing home a closely united collective, providing a solid guarantee for the prevention of pressure injuries. That's all.

**Interviewer:**

How do institutional culture and policies influence nursing assistants' motivation to perform pressure injury prevention and management?

**Interviewee:**

Institutional culture and policies have an important impact on the enthusiasm of nursing assistants in the prevention and management of pressure injuries. First of all, a positive and healthy institutional culture can create an atmosphere of caring for the elderly and focusing on the quality of care. In such a cultural atmosphere, nursing assistants will naturally be infected, regard the prevention of pressure injuries as their important responsibility, and be more motivated to improve their skills and performance. They will hold themselves to higher standards and strive to provide better care services for the elderly. Secondly, clear and reasonable policy regulations can provide clear guidance and norms for nursing assistants. These policies include specific requirements for the prevention of pressure injuries, training systems, and incentives. Nursing assistants know clearly what they should do and how to do it, and understand that they will be recognized and rewarded for doing this work well, which will undoubtedly enhance their enthusiasm and initiative. In addition, institutional culture and policies can also affect the collaboration and team spirit among nursing assistants. If the institution advocates a culture of cooperation and mutual assistance and has corresponding policies to support teamwork, nursing assistants will be more willing to work together with their colleagues to better complete the tasks of pressure injury prevention and management. Finally, institutional culture and policies are also related to the career development and sense of accomplishment of nursing assistants. An institution that supports the growth and development of nursing assistants will allow them to see the space and opportunities for continuous improvement in this field, which will also stimulate their enthusiasm and creativity in the prevention and management of pressure injuries. In conclusion, institutional culture and policies are important factors in motivating and play a vital role in improving the effectiveness of pressure injury prevention and management.

**Interviewer:**

What motives would further empower nursing assistants to perform pressure injury prevention and management effectively?

**Interviewee:**

Promptly giving recognition and rewards, such as commendations, bonuses, and promotion opportunities, will make them feel that their efforts are recognized, and thus more motivated to do a good job. Providing training, learning resources, and development space so that can continuously improve their professional knowledge and skills will make them enthusiastic about their work and more confident in doing a good job in prevention and management. The mechanism of continuing education credits can be used to motivate nursing assistants to actively participate in training and learning, thereby increasing their work enthusiasm. Encouraging nursing assistants to obtain continuing education credits can help improve the professional level of the entire nursing team and promote the overall development of the team. Let nursing assistants establish a deep emotional connection with the elderly and feel the elderly's dependence and trust in them, which will encourage them to prevent and manage pressure injuries more attentively. Create a good team atmosphere, encourage nursing assistants to cooperate and learn from each other, and set up some healthy competition mechanisms to stimulate their enterprising spirit and enthusiasm.

**Interviewer:**

Anything else?

**Interviewee:**

There is also the power of role models. Setting an example for excellent nursing assistants can motivate other nursing assistants to work harder on prevention and management. In addition, providing comfortable working conditions and reasonable work arrangements can reduce the work pressure of nursing assistants and allow them to focus more on prevention and management.

**Interviewer:**

Okay, thank you very much for your answer. The next question is about pressure injury training. Could you please talk about the current situation of your institution's training on pressure injury prevention and management for nursing assistants?

**Interviewee:**

In our nursing home, we attach great importance to the training of nursing assistants on pressure injury prevention and management. First of all, we have a complete training system, including regular theoretical training and practical operation training. In theoretical training, nursing assistants will deeply learn about the causes of pressure injury, prevention methods, and treatment measures at different stages to ensure that they have a comprehensive understanding of pressure injury prevention. In practical operation training, nursing assistants will simulate operations through actual cases to improve their practical operation capabilities. Secondly, we will invite professional medical staff to provide training and guidance. They bring more professional knowledge and experience, so that nursing assistants can better master the skills of pressure injury prevention and management. In addition, we will continue to update the training content to adapt to the ever-changing medical environment and needs. At the same time, we will also use assessments and other methods to ensure that the nursing assistants have truly mastered the content of the training. In general, our organization has invested a lot of energy and resources in the training of nursing assistants in the prevention and management of pressure injury to ensure the health and safety of the elderly.

**Interviewer:**

Okay, in response to the current training situation you just mentioned, could you please talk about the training needs and suggestions for pressure injury?

**Interviewee:**

In terms of pressure injury prevention and management, we do have some training needs and related suggestions. For example, it is necessary to further strengthen the nursing assistants' in-depth understanding and mastery of pressure injury prevention knowledge, including the latest research results and practical experience. At the same time, more attention should be paid to the cultivation of practical operation skills in the training, so that nursing assistants can skillfully use various preventive measures. We also found that when facing some complex situations, nursing assistants may lack experience and skills to deal with them, so targeted case analysis and coping strategy training is needed. In addition, it is recommended to organize training regularly to maintain the sensitivity and professionalism of nursing assistants in the prevention of pressure injury. Interactive training activities can also be carried out to enable nursing assistants to exchange experiences and improve together. Finally, I hope to provide more practice opportunities and on-site guidance so that nursing assistants can continue to accumulate experience in actual work and improve their ability to prevent and manage pressure injury. These are the current needs and suggestions.

**Interviewer:**

Okay. Thank you very much for participating in this interview. You have summarized the current situation of nursing homes, the current situation of pressure injury training, the pressure injury capacity requirements of nursing assistants, the pressure injury training requirements and suggestions. If you have anything to add later, please feel free to contact me. Thank you very much!
