# Supplementary material for: Exploring nursing assistants’ competencies in pressure injury prevention and management in nursing homes: a qualitative study using the iceberg model
Source: BMC Nurs. 2025 Mar 27;24:333. doi: 10.1186/s12912-025-02911-6 (PMC11948734; doi:10.1186/s12912-025-02911-6)
Supplement: Supplementary file 1 — Supplementary Material 1 [file 12912_2025_2911_MOESM1_ESM.zip › Nursing teacher 1 indepth interview transcript.docx]

**Nursing teacher 1 in-depth interview transcript**

**Interviewer:**

Hello, Mrs ***. I am from ***. My name is ***. We are currently doing a study to gain an in-depth understanding of the nursing assistant's pressure injury prevention and management capabilities, training status, training needs and training suggestions from the perspective of nursing teacher, so as to provide a reference for nursing homes to formulate feasible training plans and carry out pressure injury management. During this interview, we need to record the entire interview process, but all information will be kept confidential, personal information will not be disclosed, and the interview content will only be used for research. Are you willing to participate in this interview?

**Interviewee:**

OK, no problem

**Interviewer:**

Thank you very much. Here is an informed consent form. Please sign it.

**Interviewee:**

OK

**Interviewer:**

First, please introduce your professional background and work experience, especially the experience related to the prevention and management of pressure injury.

**Interviewee:**

During my postgraduate study, I worked in a wound ostomy care center for three years. My research topic was also a topic on pressure injury prevention, mainly the effect of pressure relief mattresses on pressure injury prevention. So I had such relevant experience. After I started working in colleges and universities, the courses I taught also involved this part of the content. Later, I also maintained my own research direction and conducted a series of research projects on pressure injury prevention for clinical nurses and nursing home nursing assistants.

**Interviewer:**

Okay, then have you participated in or provided training on pressure injury prevention or management?

**Interviewee:**

Yes. First of all, I have explained the knowledge of pressure injury prevention and skills training to students in class. At the same time, as I just mentioned, I have also done relevant pressure injury prevention and management in the topic, and I have also conducted relevant training for clinical nurses based on this topic.

**Interviewer:**

Well, okay. So what role do you think nursing home nursing assistants play in the prevention and management of pressure injury?

**Interviewee:**

Nursing assistants in nursing homes are the ones who directly contact the elderly, so they are the first to come into contact with the elderly, and they should play the most important role. They are responsible for discovering and identifying pressure injury, providing preventive measures, and handling and managing pressure injury in the future, so they should play a very important role.

**Interviewer:**

Yes, yes. So what do you think of the current nursing home nursing assistants' ability to prevent and manage pressure injury?

**Interviewee:**

Well, because I have worked with them on projects and have had contact with them, so as for their current situation, according to my observation, their knowledge is a little outdated and has not been updated. They have the skills, but there may be some updated content or details that are not done well. Uh, attitude communication is not so good either. Uh, in terms of attitude, they may think it is more important, but sometimes they are busy with more work content and may be negligent. Communication skills are also very important, but they may not be as ideal as we think. To achieve these communication skills, ways and methods, there is actually room for improvement.

**Interviewer:**

What specific competencies do you observe in nursing assistants that contribute most to effective PIPM?

**Interviewee:**

Well, first of all, they must have the most basic knowledge and skills in preventing pressure injury, such as the definition, pathogenesis, and risk factors of pressure injury, including how to judge its stage, risk factors, manifestations, etc., how to conduct risk assessment, and how to provide appropriate nursing preventive measures. In addition, they also need to identify other common skin problems, and then communicate with patients with pressure injury, psychological care, etc. These are all abilities they need to have. They also need to understand that the treatment of PI can help nursing assistants provide professional care. The treatment of PI requires consideration of the patient's overall health status. Understanding the treatment of PI can help nursing assistants provide professional care. Treating PI requires considering the patient's overall health. Nursing assistants should understand the principles of treatment such as cleaning the wound, removing necrotic tissue, using appropriate dressings, and possible medications. Choosing the right dressing is essential to promote wound healing. Different wound types and stages require different dressings to maintain a suitable healing environment. When using dressings, some precautions must be understood, such as following the principles of aseptic operation and preventing wound contamination. These methods need to be mastered by nursing assistants. Choosing the right dressing is crucial to promoting wound healing. Different wound types and stages require different dressings to maintain a suitable healing environment. When using dressings, some precautions must be understood, such as following the principle of aseptic operation to prevent wound contamination. These methods need to be mastered by nursing assistants. It is also necessary to enhance the nursing assistants' understanding of the importance of pressure injury prevention and management, and to be able to practice it; in terms of skills, it is to master the most basic skills of pressure injury prevention and management, and in terms of knowledge, it is the basic knowledge of pressure injury prevention and management. Such as turning and repositioning, the selection and use of some dressings, as well as pressure relief equipment, skin care, nutritional care, etc., in terms of knowledge goals, it is to master the basic knowledge of pressure injury prevention and management, including its definition, staging, pathogenesis, etc. In addition, as we mentioned earlier, the knowledge and skills of pressure injury and prevention and management, I think if, to expand on it, then we must be able to identify high-risk groups, be able to identify whether it is a pressure injury, and at the same time, be able to stage it, and then, it is the knowledge and skills of preventive measures.

Nursing assistants need to know that there are many types of mattresses and cushions that are designed to distribute pressure and reduce the risk of PI for patients who spend a lot of time in bed or in a wheelchair. Local pressure relief devices such as wedges, pillows, and washers can be used to relieve pressure in specific areas, such as the heels, elbows, or ankles. For patients who need to sit for a long time, pressure relief chairs can provide additional support and pressure distribution. Wheelchair cushions are designed to fit the shape of the wheelchair and provide pressure relief for the sitting bones and other pressure areas. Knowledge of these pressure relief devices is important to know how to choose and use them to reduce the risk of PI. Wheelchair cushions are designed to fit the shape of the wheelchair and provide pressure relief for the sit bones and other pressure areas. Understanding these pressure relief devices is important to understanding how to select and use them to reduce the risk of PI. The prevention and management of PI requires close collaboration between nurses and nursing assistants. Our professional capabilities and experience can complement each other and work together to provide comprehensive care for patients. The care plan developed by the nurses needs to be accurately implemented by the nursing assistants. This includes turning plans, skin care, nutritional support, and other PI prevention measures.

**Interviewer:**

Yes, yes. What is your perspective on the importance of nursing assistants' attitudes or values towards PI prevention?

**Interviewee:**

Their attitudes can be said to be the cornerstone of pressure injury prevention. If the nursing assistants have a serious, rigorous and positive attitude towards pressure injury prevention, they will maintain a high degree of alertness and sensitivity in their daily work. They will pay close attention to the patient's physical condition, carefully observe every detail that may cause pressure injury, and will not miss any clues. This serious and responsible attitude enables them to detect problems in time and quickly take effective preventive measures to minimize the risk of pressure injury. They will not slack off because of the tediousness or difficulty of their work, but always maintain vigorous energy and enthusiasm to serve patients wholeheartedly. Nursing assistants who put the health of patients first, respect life and care for patients will regard pressure injury prevention as their sacred duty. They will care about the pain and needs of patients from the bottom of their hearts and keep the safety of patients in mind. They are well aware of the harm and pain caused by pressure injury to patients, so they will do their best to avoid this from happening. They will take the initiative to learn the relevant knowledge and skills of pressure injury prevention, constantly improve their professional level, and provide patients with better nursing services. Their values are also reflected in the emphasis on teamwork. They understand that pressure injury prevention is not a one-person thing, but requires the joint efforts of the entire team. They will actively communicate and collaborate with colleagues, share experiences and insights, and jointly make suggestions for the prevention of pressure injury. In addition, positive attitudes and correct values ​​can create a good working atmosphere among nursing assistants. Everyone encourages and supports each other to form a united and progressive collective. In such an atmosphere, everyone can feel their own value and importance, and work harder. At the same time, this positive atmosphere will also be passed on to patients and their families, making them more trusting and dependent on the nursing team. Self-reliance support can enhance the elderly's sense of self-efficacy, making them believe that they can control their own lives and health management. Encouraging self-reliance support for the elderly can help improve the efficiency of nursing work because the elderly can take care of themselves within their ability.

**Interviewer:**

What personality traits do you think drive nursing assistants to be proactive in PIPM?

**Interviewee:**

First, responsibility is very important. Nursing assistantss with a strong sense of responsibility will regard the health of patients as their top priority, take the initiative to understand and master the knowledge and skills of preventing pressure injury, and conscientiously implement every preventive measure. Secondly, patience is also indispensable. Pressure injury prevention is a long-term job that requires nursing assistantss to have enough patience to pay attention to the subtle changes of patients, and tirelessly adjust the patient's position and provide good care. Furthermore, carefulness is also a key trait. They can keenly perceive the potential risks that may cause pressure injury, take measures in advance to prevent them, and prevent them before they happen. In addition, a proactive spirit can also encourage them to continue to learn and improve their ability in pressure injury prevention to better serve patients. At the same time, empathetic nursing assistantss can put themselves in the shoes of patients, understand their pain and needs, and thus be more actively involved in pressure injury prevention.

**Interviewer:**

How do institutional culture and policies influence nursing assistants' motivation to perform PIPM?

**Interviewer:**

In my opinion, institutional culture and policies have a crucial impact on the motivation of nursing assistants to carry out PIPM. A positive and energetic institutional culture will create an environment that cares for patients and values ​​the quality of care. In such an environment, nursing assistants can deeply understand the significance and value of their work, and thus be more motivated to do a good job in pressure injury prevention. They will set stricter standards for themselves and strive to improve their professional abilities. Scientific and reasonable policies play an important role in guiding and protecting. Clear job responsibilities and work requirements can enable nursing assistants to know exactly what they need to do and how to do it, which helps to improve their work efficiency and quality. A sound incentive mechanism, such as rewarding outstanding nursing assistants, can greatly stimulate their enthusiasm and creativity, making them more enthusiastic in pressure injury prevention. In addition, providing adequate training and resource support can give nursing assistants enough confidence and ability to do this work well.

**Interviewer:**

What motives would further empower nursing assistants to perform PIPM effectively?

**Interviewee:**

A reasonable reward and recognition mechanism can play a positive role in motivation. Giving recognition and rewards to nursing assistants who perform well will make them feel that their efforts are recognized, and thus more motivated to do better. The sense of achievement brought by the profession cannot be ignored. When they see that through their efforts, patients have successfully avoided pressure injury or their pressure injury have improved, the sense of satisfaction will make them more confident and passionate to continue to work. A good teamwork atmosphere is also one of the important motivating factors. In the process of cooperating and supporting each other with colleagues, working together for the health of patients can make them have a stronger sense of belonging and mission.

**Interviewer:**

Okay, then please tell us about your views on pressure injury training for nursing assistants in nursing homes.

**Interviewee:**

First of all, it is very important to train nursing assistants in nursing homes, because based on our previous observations, their knowledge and skills still need to be updated and improved, so it is very important and necessary. And we have observed that they don't have much training on pressure injury, and it may be mixed in with other training. There is no special training, or a more systematic and overall training. So, I think it is still necessary.

**Interviewer:**

Okay, can you talk about the needs and suggestions for pressure injury training?

**Interviewee:**

Now, we actually recommend a combination of online and offline methods, because we also know that clinical nursing assistants are busy and hard-working, and they may not have much time to carry out a complete, offline training, so online is also a very effective way, then our nursing assistantss can use fragmented time anytime and anywhere, watch videos flexibly, and learn online. Of course, offline learning is also very important. We can strengthen some theoretical points through offline learning, which is necessary. So I think offline and online are more suitable. This traditional theoretical teaching is still necessary, and then add our traditional skills demonstration. In order to better understand this case, we can actually set up some pathological analysis, combined with some teaching videos, animations, and set up a scenario. We can simulate the scenario, learn with peers, and then the group can discuss. Of course, if conditions permit, opening a wound ostomy workshop is also a good method. As for the training time, the online and offline methods we just talked about are more suitable. If it is offline, the time should not be too long. It can be carried out once a week in about a month, with two classes each time; then online, because it is not restricted, consider how to let him learn more effectively. For example, the arrangement we give to students is generally that the length of the video should be appropriate, about five to ten minutes, not too long, so that they can learn more conveniently and more flexibly, and they can also learn in fragmented time. If the time is too long, there is not so much time, which will delay work. Maybe five to ten minutes will be more appropriate.

**Interviewer:**

Then I would like to ask you, after the training, what methods can be used to evaluate the learning outcomes of the nursing assistantss and the effectiveness of the training program?

**Interviewee:**

To evaluate his learning effect, the most common method is to use a theoretical knowledge questionnaire, which may be more objective to reflect his learning effect. It is some questionnaires or scales. In addition, I think it is also possible to ask the trainees to do some satisfaction surveys to evaluate or give feedback on the effectiveness of our training program, so that we can improve it later.

**Interviewer:**

Okay. In addition to the above questions, do you have anything else to add?

**Interviewee:**

No

**Interviewer:**

Okay. Thank you very much for your valuable opinions and participation. Your opinions will help us improve the management of pressure injury in nursing homes. If you have other information to add, please feel free to share it with me. Thank you again.
