# Supplementary material for: Exploring nursing assistants’ competencies in pressure injury prevention and management in nursing homes: a qualitative study using the iceberg model
Source: BMC Nurs. 2025 Mar 27;24:333. doi: 10.1186/s12912-025-02911-6 (PMC11948734; doi:10.1186/s12912-025-02911-6)
Supplement: Supplementary file 1 — Supplementary Material 1 [file 12912_2025_2911_MOESM1_ESM.zip › Nursing teacher 2 indepth interview transcript.docx]

**Nursing teacher 2 in-depth interview transcript**

**Interviewer:**

Hello, Mrs ***. I am from ***. My name is ***. We are currently doing a study to gain an in-depth understanding of the nursing assistant's pressure injury prevention and management capabilities, training status, training needs and training suggestions from the perspective of nursing teacher, so as to provide a reference for nursing homes to formulate feasible training plans and carry out pressure injury management. During this interview, we need to record the entire interview process, but all information will be kept confidential, personal information will not be disclosed, and the interview content will only be used for research. Are you willing to participate in this interview?

**Interviewee:**

OK

**Interviewer:**

Thank you very much. Here is an informed consent form. Please sign it.

**Interviewee:**

OK

**Interviewer:**

First, please introduce your professional background and work experience, especially the experience related to the prevention and management of pressure injury.

**Interviewee:**

As for my professional background, I teach basic nursing. In the cleaning and nursing section of basic nursing, which is a chapter on skin care, pressure injury are explained as a key content. So I have a relatively good understanding of the knowledge related to pressure injury and what content to teach. Then, my work experience may be relatively less. Because our professional teachers may only go to the clinic during the winter and summer vacations, and they may not meet such people all at once, so they have less work experience. And another thing is that in addition to the professional teaching, we also have the training of nursing assistants in this school, and the training of nursing assistants also includes this content. So I have some understanding of these two major areas.

**Interviewer:**

Okay, I want to ask, you mentioned the training of nursing assistants just now, so the training involves the knowledge related to the prevention and management of pressure injury, right? Yes, what are they involved? What are the related contents? .

**Interviewee:**

The content of our training includes the first definition of it, and the population prone to pressure injury. Because our medical caregivers do not have a high level of education, we will not talk about it in depth. Including its several stages, and what should we do in several stages? That is, for the operations that medical caregivers need to do, such as turning over frequently, we will talk to them about the relevant content, so that it will not be particularly deep and difficult to understand.

**Interviewer:**

Okay, then what role do you think nursing assistants in nursing homes play in the prevention and management of pressure injury?

**Interviewee:**

I think the role of nursing assistants is equivalent to being with them day and night, and they actually have the most contact. If medical caregivers or nursing caregivers can do this well in their daily care, it can actually protect the patients' all-round health, reduce their later medical expenses, and their subsequent pain from diseases, which can be reduced a lot. So I think it is quite important.

**Interviewer:**

Okay, then what do you think of the current nursing assistants in nursing homes in terms of their ability to prevent and manage pressure injury?

**Interviewee:**

In this regard, it just so happens that we have recently come into contact with some nursing caregivers. From my previous point of view, I think they may not be very good in terms of knowledge and skills, and they will have this kind of past cognition. But recently, I think through getting to know them, I think they are quite knowledgeable in this area. When asked about some knowledge, they can still answer. Although not very professional, it seems that they should have some relevant training in clinical practice, right? Because if he didn't have this training, he might not be able to say anything when we communicated with him today. But he can say something today, and he said it with a kind of pride and pride. I feel that he may have learned or memorized the knowledge. Then in terms of skills, because it involves this aspect of skills in our teaching, for the skills of pressure injury prevention, we only have a back massage, and there don't seem to be many other operations. Frequent turning means that in the process of carrying patients, we must pay attention to not rub, that is, push, pull or pull hard. I think they have mastered these skills. Then in terms of attitude, the current attitude is at a medium level. Sometimes they don't pay much attention to pressure injury prevention because they are busy with work. In terms of communication, I didn't understand how they communicate in actual work. But our current wave of people are all around 50 years old, and there are more people in their 40s and 50s. They should have good communication skills, not just in the area of pressure injury, and they should have good clinical communication skills.

**Interviewer:**

What specific competencies do you observe in nursing assistants that contribute most to effective PIPM?

**Interviewee:**

As a nursing teacher, I have observed that nursing assistants need to have a series of key competencies in the prevention and management of pressure injury. Nursing assistants need to be able to regularly and carefully observe and evaluate the skin conditions of the elderly, especially those who are bedridden or have limited mobility. Early identification of skin changes, redness, swelling or damage at pressure points is the first step in preventing pressure injury. This is not just technical observation, but also the care and vigilance of the nursing assistants, who can detect abnormalities at an early stage and report them in a timely manner. Nursing assistants must master the correct skin care techniques, especially in terms of keeping the skin dry, avoiding friction and eczema. For example, how to clean, how to use appropriate skin care products, and how to keep the skin of bedridden elderly people breathable. These technical operations require not only standardized skill training, but also flexible application in actual operations. The prevention of pressure injury is inseparable from the caregiver's ability to adjust the body position. Caregivers need to have the ability to help the elderly turn over regularly and know how to reduce local pressure. At the same time, they must master safe movement techniques to avoid friction or pressure on the elderly's skin during movement. This requires not only sufficient physical strength and skills, but also accurate judgment of the frequency of turning over and moving. Caregivers must be able to communicate effectively with the care team and family members. In the prevention and management of pressure injury, caregivers are often the most direct observers of the skin condition of the elderly, so their feedback is crucial. Timely communication with nurses, doctors or family members about changes in the elderly's skin and ensuring smooth information flow are critical for rapid intervention. Caregivers also need to master the correct use of walkers to ensure that patients can move and transfer safely. Mastering the use of walkers can help reduce the risk of patients falling, especially when patients try to stand or walk for the first time. Nursing assistants need to master the correct use of walkers to ensure that patients can move and transfer safely. Mastering the use of walkers can help reduce the risk of patients falling, especially when they first try to stand or walk.

We also need to have the ability to educate patients, right? You need to have relevant knowledge reserves, and then when the patient becomes your patient, right? You need to take care of him and educate him, telling him that long-term bed rest and lack of nutrition may lead to the risk of pressure injury. We need to teach him this knowledge. The second thing is prevention. In work and life, we have the ability to predict this in advance and give him as much care as possible. Then we may have something that patients cannot do, that is, we can help patients turn over more often, and we should be gentle when we operate on them in their daily lives. In terms of diet and nutrition, we need to prepare a variety of meals for them, such as nutritional care and nutritional intake.

**Interviewer:**

Okay, then please tell us your opinion on the additional training of nursing assistants in nursing homes. Do you think it is necessary? .

**Interviewee:**

It is definitely necessary. Because we know that many patients, in fact, most patients admitted to nursing institutions will also have a bedridden situation. For this training on bed pressing, it can effectively reduce the risk of bed pressing, reduce the pressure of later care, and medical expenses.

**Interviewer:**

Yes, yes. What is your perspective on the importance of nursing assistants' attitudes or values towards PI prevention?

**Interviewee:**

The attitudes and values of nursing assistants often determine their sense of responsibility, enthusiasm and persistence in their work. Whether nursing assistants have a high sense of responsibility for pressure injury prevention is a key factor in determining whether prevention work is effective. Even with the best skills, if nursing assistants lack a sense of responsibility for their work, they are likely to ignore some early signs of pressure injury risk or not be meticulous in the care process. Professional ethics require nursing assistants not only to complete their tasks, but also to do their best and pay attention to the comprehensive care needs of the elderly. When facing challenges in preventing and managing PI, nursing assistants need to take the initiative to solve problems instead of evading responsibilities. This attitude helps to solve problems quickly and effectively. When faced with challenges in PI prevention and management, nursing assistants need to proactively solve problems rather than avoid responsibility. This attitude helps resolve problems quickly and effectively. Nursing assistants who do not shirk responsibility are more likely to learn from their experiences, continue to grow, and improve their professional abilities. The stronger the sense of responsibility of the nursing assistants, the more they can recognize the importance of pressure injury prevention from the bottom of their hearts and take the initiative to implement preventive measures. Whether the nursing assistants have empathy for the physical and psychological pain of the elderly directly affects their performance in pressure injury prevention. If the nursing assistants have a high degree of empathy, they are more likely to understand the discomfort and pain of the elderly and are more willing to reduce this pain through daily preventive measures. For example, the formation process of pressure injury is usually slow and hidden. If the nursing assistants lack care for the elderly, they may ignore some important signals. Empathy enables nursing assistants to be more sensitive to the needs of the elderly, especially when facing those elderly who are bedridden for a long time and cannot express themselves. This kind of care is particularly important. Pressure injury prevention is a long-term and repetitive task, and caregivers need to have great patience and persistence. Due to the complex physical conditions of the elderly, challenges such as fragile skin and difficulty in moving are often encountered during the care process. If caregivers lack patience, they may feel frustrated because of the tedious work or the low cooperation of the elderly, thus reducing their commitment to the work. On the contrary, caregivers with patience and persistence can maintain a stable service quality in the long-term care process and can consistently perform even in the face of repetitive work. Pressure injury prevention is not only a technical task, but also requires caregivers to have respect and humanistic care for the elderly. If caregivers can respect the elderly from the bottom of their hearts and regard them as objects that need care and protection, they will pay more attention to the prevention of pressure injury and take care of every need of the elderly in detail. Respect is not only reflected in the behavior of caregivers, but also in their language and attitude, which can provide a more dignified care experience for the elderly. The attitude of caregivers not only affects their personal work results, but also affects their cooperation with other medical staff. Pressure injury prevention requires the joint efforts of the team, and good communication and collaboration between caregivers, nurses and doctors is very important. If the caregivers have a strong sense of teamwork, they will be more willing to share information and exchange ideas with other care team members to ensure that the elderly receive the best care. Those caregivers with negative attitudes or lack of cooperation may ignore the importance of teamwork and affect the overall care effect.

**Interviewer:**

What personality traits do you think drive nursing assistants to be proactive in PIPM?

**Interviewee:**

I think whether caregivers can be proactive in PIPM is often deeply influenced by their personality traits. Empathy drives caregivers to take the initiative to care about the physical condition of the elderly. Caregivers with empathy can put themselves in the shoes of the elderly to understand the discomfort and pain of the elderly, especially for those who are bedridden for a long time, pressure injury can cause great pain. Caregivers with this trait will be more sensitive to the needs of the elderly and take proactive measures to prevent pressure injury instead of waiting until the problem occurs. They tend to pay more attention to details to ensure that the elderly receive the best care. Self-motivation is the key driving force for caregivers to show initiative in their work. Caregivers with this trait will not be satisfied with just completing basic tasks, but will constantly seek opportunities to improve the quality of care. For example, they may take the initiative to learn new methods and techniques for pressure injury prevention, or spontaneously perform additional skin checks or turning care without clear instructions. Self-driven caregivers tend to be better able to adapt to changing work environments and show a proactive attitude when facing challenges. The key to preventing pressure injury is to detect problems early. Caregivers with carefulness and observation can proactively detect subtle changes in the elderly's skin and take timely measures to prevent problems from worsening. They are usually very sensitive to details, regularly check the elderly's skin condition, and notice subtle changes in the elderly's position or movement, which may affect the occurrence of pressure injury. Careful caregivers will be more focused at work and ensure that every step of care operations meets standards. The prevention and management of pressure injury is a process that requires continuous attention, and caregivers need to have enough patience to maintain stable and high-quality care in long-term care work. Patient caregivers will carefully implement each preventive measure, and will not ignore any details due to boredom or fatigue even when faced with tedious work or repetitive tasks. They are able to persevere in performing necessary nursing operations, such as turning the patient over regularly and keeping the skin dry, even though these operations may seem trivial but are crucial.

**Interviewer:**

How do institutional culture and policies influence nursing assistants' motivation to perform PIPM?

**Interviewee:**

Institutional culture shapes the nursing work environment, while policies provide a clear work framework and guidelines for caregivers. Whether the institution provides continuous professional development opportunities, especially training specifically for pressure injury prevention, directly affects the motivation of caregivers. Caregivers who are able to receive professional training regularly are usually more confident in their knowledge and skills of PIPM and are aware of the practical benefits of this knowledge to patients. Through professional training, caregivers can not only update their skills in pressure injury prevention, but also feel that their work is more meaningful because they have more professional growth opportunities, which further motivates them to take the initiative to take pressure injury prevention measures at work. Reasonable arrangement of workload is also an important factor affecting the motivation of caregivers. If the institution has sufficient human resources and can reasonably allocate nursing tasks, caregivers will have more time and energy to focus on pressure injury prevention. On the contrary, excessive workload may make caregivers only deal with the most urgent nursing tasks, while ignoring seemingly "non-urgent" but actually very important tasks such as PIPM. In a long-term overloaded work environment, caregivers may feel tired and frustrated, and then lose their enthusiasm for PIPM. Incentive mechanism is an important factor in enhancing the enthusiasm of caregivers in pressure injury prevention. If the organization can promote nursing quality through reward and punishment measures, such as commending nursing assistants who perform well in pressure injury prevention, or providing rewards in the form of bonuses, honors, etc., nursing assistants will feel that their efforts are recognized and valued, and thus they will be more willing to actively participate in pressure injury prevention. Even simple verbal praise or thanks can enhance nursing assistants' sense of pride and belonging and stimulate their enthusiasm for work.

**Interviewer:**

What motives would further empower nursing assistants to perform PIPM effectively?

**Interviewee:**

Professional pride and sense of accomplishment are the core motivations for nursing assistants to continue to invest in pressure injury prevention. When nursing assistants are able to realize their contributions in the nursing process and see the positive impact of their efforts on patient health, they will have a strong sense of accomplishment. Especially when successfully preventing or managing pressure injury, if nursing assistants can be affirmed and recognized and feel that their work is crucial to improving the quality of life of patients, this sense of accomplishment will greatly enhance their enthusiasm for work and motivate them to continue to maintain high standards of nursing quality in their future work. Although intrinsic motivation is the key to continuously promote the efficient work of caregivers, external rewards and incentives cannot be ignored. Through appropriate external incentives, such as bonuses, commendations, annual outstanding employee selection, and even small rewards at work, the enthusiasm of caregivers can be significantly improved. The reward mechanism can not only encourage them to strictly implement PIPM in their daily work, but also make them feel the important role they play in the team, so that they are more willing to show more initiative and responsibility in their work. A supportive working environment can help caregivers better devote themselves to the prevention and management of pressure injury. If the nursing team and the entire organization can provide caregivers with sufficient resources, clear policy guidance and effective teamwork mechanisms, caregivers will feel that their work in preventing pressure injury is not isolated, but supported by the entire team. This supportive environment can inspire caregivers to be more proactive in their work, and at the same time reduce their anxiety or negative emotions caused by insufficient resources or lack of information. The sense of identity of teamwork and common goals will further motivate them to do a good job in PIPM. Career development is an important external motivation for caregivers to remain efficient and positive at work. If caregivers can see that they have the opportunity to get promotion and more development opportunities in their careers through efficient pressure injury prevention and management, they will be more willing to invest more energy and effort in their daily work. The motivation for career advancement is not only to obtain promotion or financial rewards, but also to gain a greater sense of professional achievement by taking on more responsibilities and demonstrating their abilities. Therefore, institutions can establish career development paths and clarify the promotion opportunities that caregivers may get at different stages of their work, so as to stimulate their continued investment in their work.

**Interviewer:**

Okay, then please tell us about your views on pressure injury training for caregivers in nursing homes.

**Interviewee:**

Pressure injury is a common problem in nursing homes. The elderly are particularly prone to pressure injury due to long-term bed rest, decreased mobility, and fragile skin. Therefore, caregivers must have solid knowledge and skills in the prevention and management of pressure injury. Training can not only help caregivers master the formation mechanism, early identification signals, prevention measures and treatment methods of pressure injuries, but also improve their nursing awareness, especially to take more active preventive measures in daily nursing work, such as regular turning over and skin examinations. Through systematic training, caregivers can identify factors that increase the risk of pressure injury (such as moisture, malnutrition, friction, etc.), so as to provide personalized care in a more targeted manner in daily work. This helps to improve the quality of care, reduce the incidence of pressure injury, and improve the quality of life of the elderly.

**Interviewer:**

Okay, please talk about the needs and suggestions for pressure injury training?

**Interviewee:**

The content of the training - for example, how do we teach patients how to turn over, and if the patient has a pressure injury, he needs a dressing, how to use the auxiliary materials, and how to choose. In addition, we will also use various devices to reduce pressure in clinical practice, and how to use the equipment must also be learned. In terms of theory, as I have always believed, we must tell patients and nursing assistants how to evaluate and identify risk factors for pressure injury. That is, the prone site, and there is also a staging of pressure injury. I think staging may be the focus of our medical teaching. But for nursing assistants, we may not explain it in such detail. In this area, I personally don't think it is necessarily the key point. It may be just passed by, just knowing it is enough. Another thing I want to know is about the pathogenesis of pressure injury. In fact, it should be counted as the points just mentioned, and it can also be mentioned separately. Because you want the caregivers to understand this and be able to do these knowledge, then the pathogenesis may also need this kind of method, that is, to make him understand it through easy-to-understand methods. In this way, he may do a better job in operation and later communication with patients, and psychological care. Regarding the pathogenesis, we have also strengthened humanistic care, right? Including the knowledge of patient psychological care. If the patient has this situation, most patients will be anxious and anxious when they see that the wound has not healed. Then how we do a good job of care is also a relatively important thing, to do a good job of psychological care.

I think that in clinical practice, pressure injury will be confused with some other skin problems. Maybe we need to tell the patients what pressure injury look like in our nursing, and other skin problems, such as diabetic foot, right? They need to be distinguished, right? There are also cases such as incontinence-related dermatitis, which have similar but different features, so we need to make a distinction.

**Interviewer:**

Do you have any suggestions for training methods?

**Interviewee:**

For teaching methods, we will use some methods such as case teaching, which is to carry out related learning through a case, and then have group discussions, and then assign tasks, complete related tasks, and acquire knowledge in the process of completing tasks. Sometimes we also have some role-playing methods, so that the classroom becomes active. Then when learning knowledge, we will remember it more.

It is recommended to adopt this kind of online and offline mixed teaching. For example, I can make part of the theoretical knowledge into micro-classes, and then put it online, that is, on the platform, so that our students can use fragmented time to read and learn. Then offline, we can use the knowledge we have learned to consolidate it offline and practice it.

Regarding the time and frequency of training, I think it must not be a long line, and long-term training may make people tired. In this way, knowledge cannot be connected in series. I personally suggest that the entire training time should not exceed one month. Because it does not have much knowledge itself? You can just condense it to within a month. Then, for example, we can start online learning in the first week, which is already two weeks, and then we can carry out clinical practice in the next two weeks. In this way, I think they will be very efficient in learning.

**Interviewer:**

Okay, then how long do you think this offline training is, for example, the workshop you just mentioned, or the small scenario teaching, or the group discussion teaching, that is, how long is one training? I think.

**Interviewee:**

One and a half hours to two hours is more appropriate, which is what I have accumulated in my life. For example, my child is in the training class, and I found that after one and a half hours to two hours of learning, he can really master the knowledge points, so I think our students can achieve the goal by strengthening and consolidating their learning in one and a half hours or two hours.

**Interviewer:**

Okay, that is, I want to know, for example, we just talked about training. If the training is over, what methods do you think can be used to evaluate the learning results of the Internet, or the effectiveness of the training program we developed.

**Interviewee:**

First of all, the first is the exam, there must be a theoretical exam. Then the second is the practical test (practical assessment). In addition, some of our attitudes and humanities can actually be seen in practice.

In addition, through some evaluation scales, it can reflect the extent of their learning.

**Interviewer:**

Okay, do you think the follow-up and support after training are important to ensure the success of practical application?

**Interviewee:**

First of all, from the employer's perspective, I think we can hope to provide relevant support policies, including time and cost. For example, our training can give our caregivers paid study during their rest time. I think it is the same as the caregiver's further study, because if our nursing assistants go out for training, they are also paid. Our caregivers can go out for training with pay. I think this is quite an incentive policy, and I hope that employers can consider it. In addition, we can consider the time after learning, and give him the corresponding time to rest. And we hope that from the employer's perspective, we can give him time, and give him free time, right? Because sometimes when we go out for training, we will indeed encounter that the caregivers may spend their own money. But if the employer can reimburse him, I think his learning performance may be a little higher. On the personal side, individuals should have an attitude of continuous learning, that is, they can be strict with themselves, because learning requires one to be proactive, and it is better to have a sense of initiative. It would be best if there is a lens of continuous learning.

**Interviewer:**

Okay. In addition to the above questions, do you have any other suggestions or opinions to help us better design the training program for the prevention and management of bed pressure injuries in nursing homes?

**Interviewee:**

Let me think about it. We hope that in the training of nursing home caregivers or related caregivers on pressure injury, we hope that our caregivers can have more support from relevant policies. I think it will be better. Because if you are supported by the policy, I think his motivation will be more sufficient.

**Interviewer:**

Okay, thank you for your valuable opinions and participation. Your opinions will help improve the parent management work of nursing homes. If you have other information to add, please feel free to share it and contact me at any time. Okay, thank you again.

**Interviewee:**

I suddenly remembered that the Ministry of Civil Affairs issued the service-related specifications for the prevention of pressure injuries in nursing homes in 2019. So our Ministry of Civil Affairs specifically took out the knowledge of pressure injury to explain. So it is actually very important at the national level. And it seems to be an important assessment indicator of the service quality of our nursing homes, right? So if we highlight the importance of this, I think that when we carry out training later, we will have a foundation in this regard, and I think the resistance from all sides will be less. Everyone can give a lot of support from the unit, from the individual, and from the social level. I think this is actually a relatively good policy support, right? Well, I don’t know if we mentioned the evaluation tool just now. In fact, I have been thinking whether we should tell our caregivers about this evaluation tool, because I saw a few scoring tables, you use something like broaden, right, braden, button, etc. Yes, because I sometimes can’t remember the English words, those screens, in fact, we feel that it is difficult to hear the name introduction. But sometimes I see that the content is quite simple, so I have never been sure whether to tell our caregivers about this thing, so I also hope to standardize this part in the later training. Whether to tell the caregivers or not, and to what extent if I do, I hope to do a good job in this aspect. Also, when we are teaching the caregivers, we want to improve their understanding. We can tell them about the incidence of pressure injury at home and abroad, especially the pressure beds in nursing homes, and how high the incidence of patients is, so that they can be aware of it at once, because they may not be exposed to it very much. But we tell them from a macro level that the incidence rate in nursing homes is so high, how much pain it brings to us and how much burden it brings to the country, right? Then they may think that it is so important, and I can relieve the pain of patients and bring support to the country by studying hard, right? I think that from a macro level, these should be mentioned a little bit.

**Interviewee:**

Okay, I think there are other things that we should do besides turning over. I also think of skills. We have the channel of bed translation to bed car, bed translation to flat car, bed to wheelchair, and wheelchair care. I think skills should also be strengthened. Because now whether it is the skill assessment of students, the skill assessment of nursing care, or the skill assessment in clinical practice. Last time we went to the First People's Hospital for a medical caregiver training, they took this as a key point in the assessment. So I think the training in transfer is also something that should be mentioned in the pressure injury. Because this also involves the patient, frequent friction will occur, right?

There is also the rehabilitation care of the later patients, that is, the patients who have severe pressure injury, if they have undergone surgery and do the relevant care, the later rehabilitation, if there is organic damage to him, in fact, this piece of knowledge should still be involved a little bit, right? Because ours is a special training after all, the knowledge should be explained in depth and thoroughly, and all aspects should be considered. There is nothing else, I will talk about it later when I think of it.

**Interviewer:**

Okay. In addition to the above questions, do you have anything else to add?

**Interviewee:**

No

**Interviewer:**

Okay. Thank you very much for your valuable opinions and participation. Your opinions will help us improve the management of pressure injury in nursing homes. If you have other information to add, please feel free to share it with me. Thank you again.
