# Supplementary material for: Exploring nursing assistants’ competencies in pressure injury prevention and management in nursing homes: a qualitative study using the iceberg model
Source: BMC Nurs. 2025 Mar 27;24:333. doi: 10.1186/s12912-025-02911-6 (PMC11948734; doi:10.1186/s12912-025-02911-6)
Supplement: Supplementary file 1 — Supplementary Material 1 [file 12912_2025_2911_MOESM1_ESM.zip › Wound care nurse 1 indepth interview transcript.docx]

**Wound care nurse 1 in-depth interview transcript**

**Interviewer:**

Hello, Mrs ***. I am from ***. My name is ***. We are currently doing a study to gain an in-depth understanding of the nursing assistant's pressure injury prevention and management capabilities, training status, training needs and training suggestions from the perspective of wound care nurse, so as to provide a reference for nursing homes to formulate feasible training plans and carry out pressure injury management. During this interview, we need to record the entire interview process, but all information will be kept confidential, personal information will not be disclosed, and the interview content will only be used for research. Are you willing to participate in this interview?

**Interviewee:**

OK, no problem

**Interviewer:**

Thank you very much. Here is an informed consent form. Please sign it.

**Interviewee:**

OK

**Interviewer:**

First, please introduce your professional background and work experience, especially the experience related to the prevention and management of pressure injury.

**Interviewee:**

I was previously in the Wound Care Center of Nanjing General Hospital. In 2017, my postgraduate studies and my supervisor were mainly engaged in the work of wound ostomy incontinence. Every day, we would deal with a large number of patients with pressure injuries. After graduating from graduate school, I entered Yancheng City Hospital and have been working in the Department of Neurology until now. In addition to daily nursing, I also participated in the prevention and management of pressure injuries for patients in a ward, because we also know that for patients in the Department of Neurology, stroke patients have many high-risk factors for pressure injuries, which means that the incidence of pressure injuries in this type of patients is also very high. In our department, I mainly assume such a role and position in the prevention and management of pressure injury.

In addition, I am also a member of our wound ostomy incontinence professional group in the hospital, so we also need to regularly conduct a quality control of pressure injuries throughout the hospital, and our group members and our hospital nurses are also trained in the knowledge of pressure injuries. From September to December 2022, I went to Nanjing International Stoma Therapist School to study and successfully passed the assessment, thus obtaining a certificate of Jiangsu Province Wound Stoma Incontinence Specialist Nurse. After returning, of course, I continued to work on pressure injuries, not only managing the skin of our neurology patients, but also conducting some quality control on the skin of patients throughout the hospital. I also gave some related lectures in some continuing education classes on wound stomas in our Yancheng City.

**Interviewer:**

Well, okay, thank you very much for your introduction. From your introduction, it can be seen that you have rich experience in pressure injury management, so we would like to ask you, that is, you just mentioned that you have participated in training on pressure injury prevention and management. Can you tell me specifically what types of training these are and which groups of people are trained, as well as the specific introduction of related training courses?

**Interviewee:**

It is like this, Mr. Guo. The main thing about my training is the training of the members of our professional team. In terms of the scope, the most extensive one is that I may provide some training related to pressure injuries for some nurses or nursing assistants in the city in the continuing education class related to wound ostomy held in Yancheng City. The training course may mainly focus on the identification and treatment of pressure injuries and other skin injuries. On a smaller level, I may provide training related to pressure injuries for nurses in our hospital, ordinary nurses, or members of our professional team. In this way, these trainings may be more focused on preventing pressure injuries in key departments such as ICU and operating room. Last year, because the province issued a quality control tool kit, the pressure injury tool kit was interpreted in batches for nurses in the hospital and members of this professional team.

In addition, we may provide regular training for the nursing assistants in our hospital. Because our hospital cooperates with a South China company, our hospital provides some professional nursing assistants. For these nursing assistants, we also provide some training on pressure injuries. Then this training may be more limited to the staging, clinical manifestations, and common skills of pressure injuries, such as turning over and position transfer skills, a series of training.

**Interviewer:**

Well, OK, you mentioned that your main population is the nurses in your city or the nurses in the hospital, and the nursing assistant in your hospital, right? So have you participated in the training related to nursing assistants in nursing homes before?

**Interviewee:**

Well, I have also been exposed to the training of nursing assistant in nursing homes. Although it is rare, it is held every year, because our hospital has a health education and regularly holds a health education activity in the community. So sometimes we may go to some ordinary communities and sometimes to some nursing homes, and then we will give some lectures on the prevention of pressure injuries to the staff of these communities or nursing homes, as well as some patients and their families.

**Interviewer:**

Yes, yes, yes, then the training you mentioned just now is probably a kind of specialized and systematic training. Is the training about pressure injury a theme, or is it integrated into all the regular trainings and is it just a part of other trainings?

**Interviewee:**

Teacher Guo, we don’t say that we have a special training or special training. Generally, it is all specialties, or it may be all specialties. Generally, all specialties come together to train this series of people, so the prevention of pressure injuries may be just a part of it.

**Interviewer:**

Well, OK, can you tell me how much time or class time the pressure injury part of your training course takes up in the entire training course?

**Interviewee:**

Each training session may take about 45 minutes.

**Interviewer:**

So it is 45 minutes. Do you think it can enable the trainees to master some skills related to pressure injury?

**Interviewee:**

That is definitely unlikely to be achieved, because the course of pressure injury is a systematic course. If you want to learn it well, that is, to learn it or finish it, 45 minutes is definitely not enough. If you want to learn it thoroughly, 45 minutes is even less. So we will only teach some basic knowledge in these 45 minutes. As for how well he has mastered it, it may not be reflected in these 45 minutes.

**Interviewer:**

Well, OK, then I will ask the next question, which is what role do you think nursing assistant in nursing homes play in the prevention and management of pressure injuries?

**Interviewee:**

Well, although our nurses have a longer contact time with patients, in fact, whether it is the nursing assistants in our hospital or the nursing assistants in the nursing home, their contact time with patients is definitely much longer than their family members or nurses. Therefore, the nursing assistant in the nursing home has the longest contact time with patients every day, longer than family members or nurses. He has to provide these patients with some daily care, including feeding, scrubbing and cleaning the skin, and turning over. Moreover, when they wipe the body of the elderly and turn over every day, they can observe the physical condition of the elderly and the integrity of the skin. It can observe the changes in the skin most directly. They are a type of personnel who can identify the risk of pressure injury early, so they play a very critical role in the prevention of pressure injury.

Because our pressure injury may be a lot for bedridden elderly people in nursing homes. It is a common complication, especially for those who are comatose, paralyzed, malnourished, and extremely thin. Well, we all know that pressure injuries will not only increase the medical expenses and pain of patients, but also may lead to death and endanger their lives if they are seriously infected. And everyone knows that the prevention of pressure injuries is definitely better than treatment. Therefore, as the important responsible persons of the elderly and the people who have the longest contact with them, if the nursing assistant of nursing homes can do early prevention and master some knowledge of the prevention and management of pressure injuries, it is crucial to eliminate or reduce the occurrence of pressure injuries.

**Interviewer:**

Okay, then in terms of the current ability of nursing assistant in nursing homes to prevent and manage pressure injuries, what do you think is their ability, such as knowledge and skills, and his professional quality and attitude towards the prevention of pressure injuries, and his other qualities and abilities, such as communication skills, etc. What is the current situation?

**Interviewee:**

Well, I think their current situation, in terms of knowledge, is that the nursing assistant may not understand a series of theoretical knowledge about pressure injuries, and sometimes they cannot identify the risk factors and stages of pressure injuries. For example, when they often turn the elderly over, you can often see that their movements are very rough, and there are often a series of unreasonable and inappropriate movements, such as dragging and pulling, which will increase the risk of pressure injuries. They do not understand that this is a risk factor for pressure injuries. In addition, it cannot actually identify what pressure injuries are. For example, some patients, such as the heel, may have intact skin but a little purple after being under pressure for a long time. In this series, we know that it may be deep tissue injury in the stage of pressure injury. But they cannot understand that they may just think that it is a simple redness of the skin and the skin will recover on its own. We know that deep tissue injuries generally do not heal on their own, and they will not recover, so they will not deal with it in time, which will lead to further deterioration and even infection. Well, in addition, their operating skills, as just mentioned, are equivalent to some body position shifts and dragging when turning over. Including some skin care, they may use soap to wash our elderly skin. We know that our skin is weakly acidic. If we use soap, it is actually alkaline. It is definitely not suitable for bathing the elderly, especially for the elderly with thin skin and risk of pressure injury. However, they may not know this. In addition, they may also use irritants such as alcohol on the patients. If there is a wound, they will deal with it, which is also unreasonable.

Then more likely is their turning over skills and body position transfer skills, which we can see intuitively, are definitely skills, which are far from meeting our needs for pressure injury prevention. In addition, in terms of attitude, they may become two extremes. One is that they may think that it may be a pressure injury, and once it occurs, it will definitely not be cured. They will actively and negatively treat such things, so they don’t even talk about serious care. Then, in another extreme, they may think that the pressure injury is not a big deal at all. They may think that it will be fine soon, and it will be fine after the scab is formed. In fact, we all know that even if the scab is formed, there may be pus underneath, and it may not be healed. So in this regard, whether they have a particularly positive attitude or a particularly negative attitude, it is actually not conducive to the treatment of pressure injuries. Well, so his attitude may not be optimistic.

In addition, in terms of communication, some nursing assistants may know that pressure injuries are very important. During the communication process with the nurse, he may rely on his long time as a nursing assistant. If a pressure injury occurs, if his attitude is positive, he may communicate with the patient's family. He may think that it will be healed immediately and there is no need to worry about it.

Then he may not have a complete grasp of some of this knowledge, which may lead to incorrect methods for daily care of turning the patient over or changing the dressing for some pressure injuries. When he communicates with the patient's family, he can bring in some of his own subjective opinions. He does not communicate with the patient and his family based on the risk of pressure injury or the stage of pressure injury, but communicates with the patient and his family based on his subjective feelings and experiences. This may lead to some difficulties for our nurses to deal with pressure injuries for patients, because the patients will say that the nursing assistant who takes care of us said to deal with it this way. In this case, communication may need to be further strengthened.

**Interviewer:**

Yes, yes. The current status of the abilities you mentioned just now is not optimistic. What specific competencies do you observe in nursing assistants that contribute most to effective PIPM?

**Interviewee:**

If we talk about his ability, it can be divided into two parts. One is the most basic ability to prevent the risk of pressure injury. Then the ability here may mainly include pressure injury. What are its high-risk factors? What is pressure injury? There are also the stages of pressure injury and some common measures for the prevention and care of pressure injury, including turning over skills, body position transfer skills, skin care, and nutritional care. For example, skin care ability. Nursing assistants are the first line personnel who have direct contact with the elderly. Mastering the correct skin care techniques can help them deal with patients' skin problems in a timely manner, ensure their comfort and health, and reduce the risk of PI. There are also some commonly used decompression equipment, such as how to assist patients to turn over and how to assist them to transfer positions. They also need to know this series of turning over equipment. Another thing is, for example, if another major area is pressure injury, if it occurs, they should know the most basic series of treatment methods, including basic wound dressing skills or operation procedures. In addition, they should also understand the role of common dressings and how to choose dressings. These are mainly the abilities of these two areas. In addition, it is crucial for nursing assistants to understand the pathogenesis and risk factors of PI, because this helps them perform PIPM more effectively. Understanding the pathogenesis can help nursing assistants take preventive measures to reduce the occurrence of PI. Identifying risk factors enables nursing assistants to conduct risk assessments on patients and determine which patients are more likely to develop pressure injuries

When doing skin examinations, nursing assistants should know which parts to focus on, such as the occipital tuberosity, scapula, elbows, sacrum, and heels in the supine position, the ears, shoulders, elbows, ribs, hips in the lateral position, the inner ankles, etc.; when doing skin examinations, nursing assistants should know which parts to focus on, such as the occipital tuberosity, scapula, elbows, sacrum, and heels in the supine position, the ears, shoulders, elbows, ribs, hips in the lateral position, the inner and outer ankles of the knees, and the ears, female breasts, male genitals, knees, toes, etc. in the prone position.

Nursing assistants need to be able to identify whether the elderly have signs of psychological problems such as depression and anxiety and report them to medical staff in a timely manner. During the care process, they need to always respect the dignity and autonomy of the elderly and make them feel respected and valued.

**Interviewer:**

Yes, yes. What is your perspective on the importance of nursing assistants' attitudes or values towards PI prevention?

**Interviewee:**

Quality is like the communication ability just mentioned. So in terms of his professional quality, I think good communication skills can enable nursing assistants to establish a good relationship with the elderly and their families, making them more willing to cooperate with the work of nursing assistants. Nursing assistants can understand the needs and feelings of the elderly through communication, adjust the care plan in time, and improve the pertinence and effectiveness of care. Secondly, in the team, communication between nursing assistants and other medical staff is also crucial. They need to promptly feedback the situation of the elderly to doctors and nurses so that everyone can jointly formulate more complete prevention and management measures. Moreover, effective communication can also help nursing assistants better understand the relevant knowledge and requirements of pressure injury prevention and management and improve their work quality. If the nursing assistants do not communicate well, it may lead to inaccurate information transmission, affecting the smooth progress of the entire prevention and management work.

In addition, some laws and regulations, professional ethics, professional norms, and laws and regulations related to pressure injury management also need to be mastered, because their legal awareness may be weaker than that of our nurses, so they may not understand some of the laws and regulations involved in our work process. So I think this series of professional qualities about laws and regulations and such communication are definitely necessary for them, and we often ignore them.

**Interviewer:**

What personality traits do you think drive nursing assistants to be proactive in PIPM?

**Interviewee:**

First, responsibility. They will take the health of every elderly person to heart, take every detail of care seriously, and will not be perfunctory because of the complexity of the matter. Second, patience. Pressure injury prevention is a long-term process. They need to have enough patience to repeat various nursing operations and pay attention to the subtle changes in the elderly's skin. Third, carefulness. They can keenly detect any abnormalities in the elderly's skin and take timely measures to avoid the deterioration of the problem. Fourth, love. They really care about the elderly, are willing to pay for the elderly, and strive to make the elderly live comfortably and healthily.

**Interviewee:**

Okay, then please tell us about your views on pressure injury training for nursing home nursing assistants.

**Interviewee:**

Well, we know that pressure injury is actually a global skin problem. With the development of the aging population, it means that more and more elderly people may live in nursing homes. Therefore, it is definitely crucial and necessary to train nursing home nursing assistants on pressure injuries, because they are the main force in caring for elderly patients. Only by improving their knowledge and practical skills of pressure injuries can we improve the quality of life of the elderly in our nursing home and reduce the occurrence of complications. In addition, because I have also come into contact with nursing home nursing assistants, they actually receive regular training organized by others. In fact, basically, including our training is basically very general, and there is rarely a special topic or special training on pressure injuries. However, we also know that according to the national standards for professional skills training for nursing home nursing assistants, pressure injuries are actually a very important part of the training content. However, due to time constraints, most institutions only provide simple training on some content, and it may also be that the person in charge of the nursing home does not pay enough attention to it, resulting in a relatively short training time.

As far as I know, some nursing homes may be able to provide two hours of training on pressure injury. For two hours, including our hospital, if you go to a nursing home, it is actually about two hours, but within these two hours, it may also involve measuring the patient's blood pressure, and then observing the patient's skin. A series of operations. The time left for patients and nursing assistants to train on pressure injuries may be a little less.

In addition, if the attitude of the person in charge is positive, that is, they will be more supportive of our work, then the effect of our training will be relatively better. However, if their person in charge is not supportive enough, then the effect of the training will not be good.

**Interviewer:**

How do institutional culture and policies influence nursing assistants' motivation to perform PIPM?

**Interviewee:**

A positive institutional culture will create an atmosphere of caring for the elderly and focusing on the quality of care, which will make nursing assistants feel the value and significance of their work, and thus be more motivated to do a good job in pressure injury prevention and management. They will hold themselves to higher standards and strive to provide better care for the elderly. A sound policy system can provide clear guidance and norms for nursing assistants, so that they know what to do and to what extent. A reasonable incentive mechanism and assessment system can also mobilize their enthusiasm, such as rewarding nursing assistants with outstanding performance, which will encourage them to work harder to do this job. On the contrary, if the institutional culture is not good and the policy system is not perfect, it may make nursing assistants feel confused and lack motivation.

**Interviewer:**

What motives would further empower nursing assistants to perform PIPM effectively?

**Interviewee:**

To further enhance the motivation of nursing assistants to effectively perform pressure injury prevention and management, I think the following points are important. First, recognition and affirmation of their work. When their efforts are seen and praised, they will be more motivated to do better. Second, provide continuous training and learning opportunities so that they can continuously improve their professional ability and have more confidence and ability to do this job well. Third, establish a good teamwork atmosphere so that they feel that they are an important member of the team and everyone works together for a goal. Fourth, give appropriate reward mechanisms, such as performance bonuses, honorary titles, etc., which will motivate them to be more proactive in the prevention and management of pressure injury. Fifth, let them understand the importance of this work to the health of the elderly and stimulate their sense of professional mission and responsibility.

**Interviewer:**

Okay, thank you very much for your answer. The next question is about pressure injury training. Could you please talk about the current situation of your institution's training on the prevention and management of pressure injury for nursing assistants?

**Interviewee:**

In our institution, we attach great importance to the prevention and management of pressure injury for nursing assistants. We regularly organize professional training courses, including theoretical knowledge explanations, such as the causes, stages, and preventive measures of pressure injury, as well as demonstrations of practical operations, such as correct turning techniques and skin care methods. At the same time, we will also analyze cases to let the nursing assistants understand the coping methods in different situations more intuitively.

During the training process, we also focus on interaction and communication, encourage nursing assistants to raise questions and discuss solutions together. In addition, we will evaluate the training effect of nursing assistants to ensure that they have truly mastered the relevant knowledge and skills. However, we also realize that there are still some areas that can be improved, such as increasing the pertinence of training, conducting tiered training according to the different experiences and abilities of nursing assistants, and providing more practical opportunities. What other opinions or suggestions do you have about the training of our institution?

**Interviewer:**

Okay, please talk about the training needs and suggestions for pressure injury?

**Interviewee:**

As for the training method, I suggest a combination of online and offline methods. Because if it is all offline training, the teaching quality cannot be perfectly guaranteed, because people's attention is limited, and for nursing assistants, their knowledge and academic qualifications are not very high, which means that their ability to accept knowledge is not strong. Therefore, some online learning is also required, and because nursing assistants are usually responsible for caring for patients and the elderly, their working hours are also busy. If it is all offline training, it may disturb their normal working state. So offline learning may need to be equipped with some online learning. What about offline learning? It has its advantages, because we can adopt various teaching modes, such as PBL, group discussion, role-playing, workshop, or self-practice, and can provide real-time feedback, which means that such a two-way communication between teachers and students can strengthen our theoretical knowledge and practical skills. Online learning allows us, the nursing assistants, to use fragmented time and choose their own learning time independently. In this way, their learning efficiency is relatively high and they can avoid disturbing their normal daily work as much as possible.

**Interviewer:**

Okay, you mentioned offline training just now. Can you elaborate on what form is more appropriate?

**Interviewee:**

Well, in the workshop, for example, case analysis, everyone will discuss in groups first, and then each group can provide the same or different cases, and then everyone will discuss and analyze, and then raise their hands to speak. In the form of groups, each group will have a representative, and then stand up to speak. There is also the kind that, for example, let a nursing assistant play the role of an elderly person or a patient, and then let other nursing assistants take care of him, and then we can experience it, that is, reverse thinking, and experience it.

**Interviewee:**

Okay, then what is the importance of follow-up and support after training to ensure the success of practical application?

**Interviewer:**

Well, I think this is very important, because this training is a systematic process, and the systematic process cannot be solved by a certain training. So the follow-up and support after your training are very important to ensure that they have mastered the knowledge. High-quality training can help nursing assistants grow, and the ultimate manifestation is the change in behavior and the change in results. If the incidence of pressure damage in elderly patients is reduced, it means that our training is useful and our training is good. Then you must follow up this process. If you don’t follow up, you don’t know what the final result will be. Because you may perform well at the beginning, but you may slack off over time. So we must ensure the effect of this training. Because this knowledge and skills are easy to forget, and we know that knowledge is changing with each passing day, and the pressure injury management guidelines and consensus may be updated frequently. So for this series of training, in addition to consolidating the previous training content, we may also need to add some new knowledge to keep up with the times. Right?

**Interviewer:**

Okay, thank you very much for your very detailed answer to the above questions. In addition to the above questions, do you have anything else to add?

**Interviewee:**

No at the moment

**Interviewer:**

Okay, thank you very much for your valuable comments and participation in this interview. Your comments will help improve the pressure injury management work of our nursing home. If you have any additional information to add, please feel free to contact me, thank you very much.

Interviewee:

Okay, thank you, Mrs. Guo.
