# Supplementary material for: Exploring nursing assistants’ competencies in pressure injury prevention and management in nursing homes: a qualitative study using the iceberg model
Source: BMC Nurs. 2025 Mar 27;24:333. doi: 10.1186/s12912-025-02911-6 (PMC11948734; doi:10.1186/s12912-025-02911-6)
Supplement: Supplementary file 1 — Supplementary Material 1 [file 12912_2025_2911_MOESM1_ESM.zip › Wound care nurse 2 indepth interview transcript.docx]

**Wound care nurse 2 in-depth interview transcript**

**Interviewer:**

Hello, Mrs ***. I am from ***. My name is ***. We are currently doing a study to gain an in-depth understanding of the nursing assistant's pressure injury prevention and management capabilities, training status, training needs and training suggestions from the perspective of wound care nurse, so as to provide a reference for nursing homes to formulate feasible training plans and carry out pressure injury management. During this interview, we need to record the entire interview process, but all information will be kept confidential, personal information will not be disclosed, and the interview content will only be used for research. Are you willing to participate in this interview?

**Interviewee:**

OK

**Interviewer:**

Thank you very much. Here is an informed consent form. Please sign it.

**Interviewee:**

OK

**Interviewer:**

First, please introduce your professional background and work experience, especially the experience related to the prevention and management of pressure injury.

**Interviewee:**

Okay, I participated in the three-month wound specialist nurse training at the Jiangsu Wound and Stoma School in 2015. After returning to the hospital, we set up a skin specialist team, which mainly carried out work in the areas of wounds and stoma incontinence, and gradually formed a series of standardized measures and systems. At present, the pressure injury area of our hospital is managed in a homogeneous manner. We have more than 30 hospital-level specialist nurses, and I am the team leader. Then in the area of ​​pressure injury, including full-staff consultations, consultations outside the hospital, and outpatient clinics, we have been working steadily and orderly.

**Interviewer:**

Yes, okay. Have you provided training on pressure injury prevention and management before?

**Interviewee:**

Yes, I have. Our regular training is to train the latest expert consensus guidelines for pressure injuries, and we will hold a specialist lecture for the whole hospital.

**Interviewer:**

So the main training targets are nurses in this hospital, right?

**Interviewee:**

Yes.

**Interviewer:**

Have you participated in pressure injury training for nursing homes or hospital nursing assistant before?

**Interviewer:**

Yes. Outside the hospital, we have a community unit, we have several cooperative township health centers, and also nursing homes. We often go to them to train . Well, then we have a full-time training plan for the nursing assistants in our hospital, and I also train them on the prevention of pressure injuries.

**Interviewer:**

Okay, then what do you think of the current status of pressure injury training for nursing assistant in nursing homes? For example, training time, methods, content, etc.

**Interviewee:**

For this topic, although there are only one or two trainings, we will pay attention to this area during our usual nursing quality inspections, that is, the application of the knowledge of pressure injury training by the nurses in the process of caring patients, and they will conduct irregular inspections. Each training session is not long. We usually have only one or two training activities a year, and each training session is about two class hours. About 90 minutes. Practical training. The content mainly includes body positioning, use of preventive dressings, etc.

**Interviewer:**

Okay, then what role do you think nursing assistant in nursing homes play in the prevention and management of pressure injury?

**Interviewee:**

I think it plays a role in prevention and guidance. At least if they are trained, they will definitely know more about the professional aspects than their family members who have not learned anything. They can at least know the importance of changing body position and at least observe the early color change of the skin, which can prevent the development of pressure injury and should be able to discover it.

**Interviewer:**

Well, okay, then what do you think of the current ability of nursing assistant in nursing homes in terms of pressure injury prevention and management?

**Interviewee:**

The current situation is definitely uneven. One is age. The quality of our grassroots nursing assistant may not be very uniform. Then everyone may have different cultural and educational levels, so for them, this training is actually a top priority.

**Interviewer:**

What pressure injury prevention and management abilities do you think nursing assistant in nursing homes should have? What specific competencies do you observe in nursing assistants that contribute most to effective PIPM?

**Interviewee:**

The first is communication ability. Nursing assistants need the ability to communicate, including with family members and our medical staff. Well, the second is his hands-on ability and skills. For example, if the position is not suitable, how should the nursing assistants turn him over? How often should he turn him over? This is his technical ability. Then the third is theoretical knowledge. What needs to be improved is the knowledge level, because pressure injury is a theoretical knowledge system. There are many contents. Sometimes they only have one or two class hours to explain, and they have only learned a little bit. For example, when changing the patient's position, he doesn't know why the position should be changed, and how often the position should be changed. Maybe they don't learn it deeply enough.

**Interviewer:**

Yes. What is your perspective on the importance of nursing assistants' attitudes or values towards PI prevention?

**Interviewee:**

In my opinion, the attitudes and values of nursing assistants are crucial to the prevention of pressure injury. Their positive attitude allows them to take each preventive measure more seriously and not ignore it because they think it is cumbersome or insignificant. For example, their enthusiasm for the elderly care profession allows them to devote themselves to their work and take the initiative to prevent pressure injury. The sense of belonging makes them regard the institution as their own home and the elderly as their own relatives, and take care of them more attentively. The commitment to work makes them perform their duties conscientiously and not perfunctorily. Comply with laws and regulations to ensure that all operations are legal and compliant. Respect the self-esteem and privacy of the elderly and make them feel respected and cared for. Treat every elderly equally and do not favor any one. Attach great importance to the prevention of pressure injury and regard it as an important task. Emphasize the quality of care and strive to do the best. Do not shirk responsibility and actively solve problems when encountering them. Carefully check the skin condition of the elderly and do not miss any details. Encourage the elderly to take care of themselves within their ability and enhance their confidence and independence. Do not blame the elderly and understand their difficulties. Actively pay attention to the needs of the elderly and think about what the elderly think. Be able to look at problems from the perspective of the elderly and better understand their feelings. Be patient and not impatient with the slow response of the elderly.

**Interviewer:**

what personality traits do you think drive nursing assistants to be proactive in PIPM?

**Interviewee:**

In the prevention and management of pressure injury, the proactive personality traits of nursing assistants are crucial. First, it is crucial to have the trait of carefully checking the skin condition of the elderly. They will carefully and meticulously observe the skin of the elderly, and can detect any subtle changes in color or abnormal touch in time, so as to take preventive measures as soon as possible to avoid the occurrence of pressure injury. Secondly, it is also important to have the trait of encouraging the elderly to take care of themselves within their ability. They will patiently guide the elderly to carry out some simple self-care activities, such as turning over and moving limbs, which can not only enhance the elderly's self-care ability, but also help prevent pressure injury. The trait of not blaming the elderly reflects their tolerance and understanding. They understand that the elderly may have some problems, but they will not blame or complain about them, but help the elderly solve the problems with a peaceful mind. In addition, the trait of actively paying attention to the needs of the elderly enables them to take the initiative to understand the various needs of the elderly, whether in life or psychology, and provide care and support in time. Finally, it is even more valuable to have the trait of being able to consider problems from the perspective of the elderly. They will understand the elderly's slow response and get along with them with great patience and tolerance, making them feel warm and cared for.

**Interviewer:**

How do institutional culture and policies influence nursing assistants' motivation to perform PIPM?

**Interviewee:**

A positive institutional culture can create an atmosphere of caring for the elderly and focusing on the quality of care, so that nursing assistants feel that their work is valuable and thus more motivated to do a good job in PIPM. For example, the institution advocates teamwork, and everyone supports and learns from each other, which will make nursing assistants feel that they are not isolated and helpless, and have more confidence to face various challenges. Reasonable policies can provide clear guidance and support for nursing assistants. A sound training system can enable them to continuously improve their professional capabilities and better carry out PIPM work; a reasonable incentive mechanism can stimulate their enthusiasm, such as rewarding nursing assistants with excellent performance, so that they feel that their efforts are recognized; a clear responsibility system can make them clear about their responsibilities and avoid buck-passing

**Interviewer:**

What motives would further empower nursing assistants to perform PIPM effectively?

**Interviewee:**

Let them deeply realize the importance of their work and understand the significance of preventing pressure injury to the health of the elderly, so that they can do it more seriously and responsibly. Provide more learning opportunities and career advancement channels to motivate them to continuously improve their professional level to better cope with PIPM work. Give them timely affirmation and praise, and give appropriate rewards for outstanding performance, which can greatly stimulate their enthusiasm. Create a good team atmosphere, let them feel the power of the team, and cooperate and help each other at work. Inspire their deep love for the elderly and let this emotion become the driving force for their hard work. Clearly inform them of the goals and requirements of PIPM so that they know the direction of their efforts. Continuously update their knowledge and skills so that they can better implement PIPM.

**Interviewer:**

Okay, can you please talk about the needs and suggestions for pressure injury training?

**Interviewee:**

As for the training method, I think first of all, on-site teaching is necessary, that is, offline teaching is definitely necessary. Well, and sometimes, for example, each unit may be busy, so it is better to combine some online classes. The training method that combines online and offline is more recommended. The offline workshop method is also better. I have done it once in our hospital. It requires a simulated person, such as the simulated mold of chronic wounds of the lower limbs, and then let them observe what the first stage wound looks like, what the second stage wound looks like, and what the third stage looks like. Well, first let them identify it, and then after identification, of course, nursing assistants are not qualified to do professional dressing changes. Well, this is called the intervention of our nursing assistant. They have to do auxiliary work and cooperate with nurses to observe and change dressings. We need to observe the dressing, to what extent it is wet and to remind the nurse to change it, and what problems should be paid attention to for the wound. For example, if the wound has a foul odor, we should remind our nurses in time to ask them to change the dressing. The nursing assistants also need to understand the process and method of changing the dressing to assist the nurses in changing the dressing.

The frequency of training needs to be determined according to the replacement frequency of the nursing assistants. The replacement frequency of the nursing assistants in our hospital is relatively fast because they are more frequent. I think the training time is currently twice a year in our hospital, and I think it is best to train once a quarter. Then as for the frequency of training, I think it is better to do one or two lessons, two or three lessons of theoretical training. But our hospital currently arranges 90 minutes, which is relatively short. If I do a workshop and do clinical case analysis and discussion, it may be better to extend the training time. Well, then you not only train, but also follow up and check the quality of his training once a quarter. How is the effect in clinical use? Maybe there will be a quality follow-up later. As mentioned earlier, we need to combine online training. Sometimes, some nursing assistants cannot come to the training because they cannot leave the patient's place. In this case, I think it is also good for them to use this online resource. The nursing assistants who cannot come can learn online independently.

**Interviewer:**

Yes, OK. You just mentioned an evaluation after the training. What kind of method do you think can be used to evaluate the learning outcomes of the nursing assistant and the effectiveness of our training program?

**Interviewee:**

I think this should be supervised from the aspect of nursing quality, that is, to formulate a quality standard for pressure injuries. This quality standard can be charged in the ward, just to see whether the body position is correct, whether the skin cleaning is done properly, etc. Regarding the evaluation of training effectiveness, the knowledge and skills questionnaire can be used to measure.

**Interviewer:**

Okay, thank you very much for your very detailed answer to the above questions. In addition to the above questions, do you have anything else to add?

**Interviewee:**

No

**Interviewer:**

Okay, thank you very much for your valuable opinions and participation in this interview. Your opinions will help improve the management of pressure injuries in our nursing homes. If you have any additional information, please feel free to contact me. Thank you very much.
