# Supplementary material for: Exploring nursing assistants’ competencies in pressure injury prevention and management in nursing homes: a qualitative study using the iceberg model
Source: BMC Nurs. 2025 Mar 27;24:333. doi: 10.1186/s12912-025-02911-6 (PMC11948734; doi:10.1186/s12912-025-02911-6)
Supplement: Supplementary file 1 — Supplementary Material 1 [file 12912_2025_2911_MOESM1_ESM.zip › Wound care nurse 3 indepth interview transcript.docx]

**Wound care nurse 3 in-depth interview transcript**

**Interviewer:**

Hello, Mrs ***. I am from ***. We are currently doing a study. The purpose is to provide a reference for nursing homes to formulate feasible training programs and carry out pressure injury management by deeply understanding and exploring the needs of wound specialist nurses for the prevention and management of pressure injury for nursing assistants in nursing homes, the current training status, training needs and training suggestions. With the deepening of population aging, nursing homes have become the habitat of more and more elderly people in their later years. In this context, the importance of nursing home nursing assistants has become increasingly prominent. Among them, the prevention and management of pressure injury, as the top priority of nursing work, is directly related to the quality of life of the elderly and the professional quality of nursing assistants. Therefore, it is very important to strengthen the training of nursing assistants for pressure injury. During this interview, we need to record the entire interview process, but all information will be kept confidential, personal information will not be disclosed, and the content of the interview will only be used for research. Are you willing to participate in this interview?

**Interviewee:**

OK

**Interviewer:**

Thank you very much. Here is an informed consent form. Please sign it.

**Interviewee:**

OK

**Interviewer:**

First, please introduce your professional background and work experience, especially the experience related to the prevention and management of pressure injury.

**Interviewee:**

Okay, let me introduce myself first. I entered Nanjing Medical University in 2005, and then graduated in 2009 to work in the hospital. After work, I also came into contact with some elderly people like these, but I really started to have more contact with them after 2014, when I started to study the knowledge related to wound ostomy. Then in 2017, I went to Nanjing International Stoma School to study related knowledge and obtained the certificate of international stoma therapist. Then I officially engaged in the prevention and management of pressure injury.

**Interviewer:**

Okay, that is, if you provide or have training on the prevention and management of pressure injury? If so, what types of training and courses have you provided?

**Interviewee:**

Okay, well, after I am engaged in wound ostomy-related work, the prevention and management of pressure injury, as well as some treatment, is a very important part of our usual work related to this profession. Normally, for patients in the hospital, we will treat the wounds of patients with pressure injury, which are now also called pressure injuries. We will also manage the wounds of patients who have already developed pressure injury. For patients who have not developed pressure injury, we will prevent and manage them. If pressure injury occur in the hospital, there will be a series of treatments. We will evaluate the cause of the injury, and then our nurses will see if there is any pressure injury caused by inadequate management. We will also conduct an appraisal. Usually, there is a lot of training for hospital nurses on the prevention and management of pressure injury, which will be held every year. There will be some knowledge and skills training programs every quarter, and pressure injury training will be held about twice a year, and each training will be about 2 hours.

**Interviewer:**

Okay, then what role do you think nursing assistants in nursing homes play in the prevention and management of pressure injury?

**Interviewee:**

I think the role of nursing assistants in the prevention and management of pressure injury is very, very important. We usually encounter patients with pressure injury in the ward. You will find that if a patient's family or his nursing assistants does not do a good job, then even if we, the professionals who are responsible for wound treatment, put in a lot of effort, that is, we change the dressing more frequently, or use more advanced and better medicine. But if his care is not in place, his wound will also develop in a worse direction. On the contrary, if the family or nursing assistants of a patient is very dedicated to the patient and has a good understanding of relevant knowledge, then for this patient, he does a good job and we do a good job. Then the patient's condition will develop in a good direction.

**Interviewer:**

Yes, then what do you think of the current management ability of nursing assistants in nursing homes for pressure injury and prevention? What do you think of their current ability?

**Interviewee:**

I think the overall quality of the nursing assistants in nursing homes is better than before. In the past, we had some patients from some local nursing homes come here and find that their skin is falling off all over their bodies, that is, there is a lot of dust. Then it means that they do not clean it properly. Then, pressure injury are not like our hospitalized patients. Some patients with serious illnesses have pressure injury mainly concentrated in the sacrum and coccyx. Those who come from nursing homes will have pressure injury all over their body, that is, the shoulders, feet, heels, sacrum and coccyx, occipital bone and other places where pressure is applied. You will find that the number of their pressure injury is particularly large, which means that their work is not done well. Now, there are fewer such patients than before, which means that many nursing homes have begun to pay attention to the management of pressure injury. However, it does not mean that what is done now is better than before, but it is not perfect, and there will still be pressure injury.

**Interviewer:**

What specific competencies do you observe in nursing assistants that contribute most to effective PIPM?

**Interviewee:**

First of all, it is very important to identify what kind of patients are likely to have pressure injury. For example, some patients have completely lost the ability to take care of themselves, or their physical condition has been poor recently. For example, in terms of diet, if the patient is not able to eat, or has shortness of breath, it means that his whole body function has changed. In this case, some patients need to go to the hospital for conditioning. There are also some patients who cannot eat and need to go to the hospital for conditioning. That is, their whole body condition has changed. How should they identify it? They need to know and assess what kind of patients are at higher risk of developing pressure injury. Because some patients are very fat, they are very heavy, and they sweat easily, or they have fecal incontinence. What is the frequency of their turning over, right? He also needs to understand that some patients are very thin, and they eat very little. From time to time, they need intubation, nasogastric feeding, etc. He may also need to make a preliminary assessment, or go to the hospital to see what the doctor thinks. For nursing assistants, they need to know the concept of pressure injury. Then they need to know what the incidence of pressure injury is, what kind of patients are more likely to develop pressure injuries, and then conduct an assessment. They also need to understand that they need to conduct a risk assessment for these patients. What kind of patients are more likely to develop pressure injuries. The risk, first of all, is the patient's position, but the forced position is still immobile, or he has incontinence, which are all related risk factors for pressure injury, and there are other endogenous factors, including whether the patient is malnourished, sensory disorders. Some people feel bad, so they sleep for a long time and don't feel pain, and there are some patients with acute diseases. For example, some of our patients in shock have circulatory disorders, and they may have exogenous factors, including pressure or first of all, if you don't turn over, if you don't turn over, the pressure will reduce your friction, which is the main cause of pressure injuries, and there is also moisture. This kind of moisture caused by incontinence, and the use of some medical devices. Then including our masks, um, and those in the prone position, when doing surgery, the forehead and chin, and the nasal mucosa where we plug the oxygen tube, and the mask on the ear, right? Ah, there are actually many things related to many medical devices. Then what to do and when to conduct an assessment.

Nursing assistants need to learn how to assess the risk of PI. Many clinical guidelines recommend the use of risk assessment tools. Nursing assistants’ understanding of these tools will help them follow these guidelines. Risk assessment provides a basis for recording and analyzing changes in patient conditions and helps monitor the effectiveness of care. Accurate risk assessment and corresponding nursing measures can reduce medical disputes caused by PI. Generally speaking, our hospital has an assessment upon admission, which is required to be conducted within two hours, and a report must be made after the assessment. If the risk is relatively high, then during the hospitalization, if the patient’s condition has changed, some assessments are also required. Then we evaluate the frequency, and the low risk uses the brand scoring scale. In our hospital, low-risk patients are assessed once a week, medium-risk patients are assessed once every three days, and high-risk and extremely high-risk patients are assessed every day. If they are in nursing homes, they can also refer to this, but they generally have fewer high-risk patients. High-risk patients need to come to the hospital. There are many assessments, but the most commonly used one for the elderly is the Brand score. Some other surgical patients and infants are not suitable. Then let's take a look at this assessment tool. Then, the score has been scored. It is assessed through several aspects, such as sensing moisture activity, moving nutrition friction and weight loss. I won't talk about this. They just need to follow this. Then the preventive measures first include hanging signs for some patients with risks, and then equipping the bed with what kind of air cushion, mattress or static pressure relief mattress. Turn over once every 2 to 4 hours. This is based on the risk classification to adjust the frequency of turning over. You can go to the hospital for a check. The regulations of each hospital may be different. Then when handing over such patients, check the skin condition. Use some preventive devices, such as silicone pads, soft pillows, etc. If the risk level increases due to changes in the condition, you need to adjust the preventive measures in time. And you need to communicate well with the patient and his family. You see low risk, medium risk. If it is high risk, the main preventive measures are different. The frequency of turning over is different. Low risk is 2~4 hours, high risk is 1~2 hours, but it also depends on the specific situation of the patient. Some people may not even need an hour, and they may need it more. Then the heel should be suspended in the air. What do you use to suspend it in the air? You can also talk to the nursing assistants about this. Then what are the measures for limited mobility? Make a turning plan, and be gentle when turning over. For some traction, these things should be placed in advance, and then a direction should be made. If the vital signs are not stable when turning over, the turning over can be done in a gradual and slow manner with a smaller angle, but it cannot be done without moving at all. If he refuses to turn over because of pain, we can use some pain relief methods in advance. To reflect on the patient with sensory impairment, he has no feeling. When turning over, he should check whether the bed sheet is clean. Because even if something hits him, he will not feel it. So for us nurses, we should first observe whether there are any things on the bed that are harmful to the patient. Then there is the treatment of urine and feces, which means cleaning urine and feces in time, and then changing clean clothes, and then getting better every time. Well, when cleaning the skin, do not use alcohol or iodine. These things are generally washed with warm water, or we use weak acid, special cleaning liquid to wash, to prevent the skin from being too dry. Well, some people will have very dry skin if they use iodine. Then after washing, you can apply some moisturizers and lotions to increase the resistance of the skin. Then we can generally use catheters, diapers, urine pads, etc. Anyway, these should keep the skin clean and dry. Then you can also use some liquid dressings, which form a protective film on the skin surface. If it is more serious, you can use some stove pockets, large and small feces wipes management devices and other things. Then about the compression of some medical devices, first of all, when you use the device, you must check whether the tightness is adapted to whether there is something to prevent pressure damage underneath, and take protective measures, and then regularly untie and check, and then hand over each shift. There are also some malnourished patients. Observe whether the patient has the risk of malnutrition. If there is some poisoning and malnutrition, he will have edema, and after edema, it is particularly easy to have some injuries. Then the thin patient has less fat protection, uh, and it is also necessary to check regularly.

**Interviewer:**

Anything else?

**Interviewee:**

Many laws and regulations set standards and requirements for nursing services. Nursing assistants need to comply with these standards when implementing PI prevention and management to ensure quality and safety of care. Laws and regulations protect patients' rights, including the right to informed consent, the right to privacy, and the right to receive appropriate care. Nursing assistants need to understand these rights and respect them in their daily work

**Interviewer:**

Yes, yes. What is your perspective on the importance of nursing assistants' attitudes or values towards PI prevention?

**Interviewee:**

As a specialist nurse focusing on wound care, I am well aware of the importance of nursing assistants' attitudes and values ​​in pressure injury prevention in providing efficient and humane nursing services. Passion for the elderly care profession is the driving force for nursing assistants to provide high-quality nursing services. Passion for this job means that nursing assistants will care for elderly patients more attentively and carefully. Passion can motivate them to continue learning and improving, which is essential for their professional development in preventing and managing PI. Passion for the elderly care profession is the driving force for nursing assistants to provide quality care services. Passion for this job means that nursing assistants will be more dedicated and attentive to the care of elderly patients. Passion can motivate them to continue learning and improving, which is crucial for their professional development in PI prevention and management. It is reflected in the fact that nursing assistants take the initiative to learn and master new nursing skills, such as the latest technology for treating pressure injury. For example, a nursing assistant in our team spontaneously learned how to use a new type of dressing, which has a special effect on certain types of pressure injury. A sense of belonging makes nursing assistants feel that they are part of the team, which motivates them to be more committed to their work. For example, we enhance the sense of belonging of nursing assistants through team-building activities and common nursing projects. Work commitment is reflected in the focus and dedication of nursing assistants to their work. For example, even on holidays, our nursing assistants will ensure that the nursing plan is carried out in an orderly manner. Nurses comply with industry standards and regulations to ensure the safety and legality of nursing work. For example, we organize regulatory training regularly to ensure that every nursing assistant is aware of the latest regulations on pressure injury care. When performing nursing operations, the nursing assistants pay attention to protecting the privacy of the elderly. For example, we use privacy curtains when changing dressings. The nursing assistants treat every elderly equally, regardless of their socioeconomic status. For example, we ensure that every elderly receives the same standard of pressure injury screening. When performing daily tasks, the nursing assistants pay special attention to the prevention of pressure injury. For example, one of our nursing assistants found that an elderly person's mattress was not suitable and promptly recommended a replacement, which effectively prevented a pressure injury. The nursing assistants are committed to providing high-quality nursing services. For example, we have implemented a quality improvement program to encourage nursing assistants to make suggestions to improve the quality of care.

**Interviewee:**

Anything else to add?

**Interviewee:**

The nursing assistants are responsible for their work and do not shirk their responsibilities. PI are a serious complication that can increase patient suffering and healthcare costs. Nursing assistants need to fully recognize the seriousness of PI and regard them as a priority in their nursing work. Nursing assistants who pay attention to PI are more likely to follow clinical guidelines and best practices and ensure that all nursing activities are based on scientific evidence. For example, one of our nursing assistants found early signs of pressure injury during a careful skin examination and treated them in a timely manner. The nursing assistants encourage the elderly to engage in appropriate activities to improve their quality of life. For example, we encourage elderly people who can walk to walk a moderate amount every day. Nursing assistants do not blame the elderly for their mistakes or misunderstandings. For example, if the elderly forget to turn over, we will patiently remind them and provide necessary assistance. Nursing assistants will proactively ask the elderly about their needs and provide personalized care. For example, we will adjust the care plan based on the elderly's feedback. Nursing assistants are able to understand the elderly's feelings and provide corresponding support. For example, we will discuss their concerns with the elderly and provide comfort and solutions. Nursing assistants are patient with the elderly's slow response. For example, we will give the elderly enough time to adapt to new care measures instead of rushing them.

**Interviewer:**

What personality traits do you think drive nursing assistants to be proactive in PIPM?

**Interviewee:**

As a specialist nurse focusing on wound care, I think the personality traits of nursing assistants are crucial to their ability to take proactive actions in pressure injury prevention and management. Nursing assistants need to have meticulous observation skills to be able to identify subtle changes in skin conditions. For example, one of our nurses noticed a tiny spot on the skin when changing the bed sheets for the elderly, which was eventually confirmed as an early sign of a pressure injury. A strong sense of responsibility means that the nursing assistants will strictly follow the care plan and ensure that all necessary precautions are implemented. For example, even when there is a shortage of staff on the night shift, our nursing assistants will ensure that every elderly person turns over on time. Empathy enables nursing assistants to better understand the feelings of the elderly and provide more humane care. For example, we have a nursing assistants who is very careful and cautious when changing the dressing for an elderly person in pain to relieve the discomfort of the elderly. Patience is necessary for nursing assistants when facing the elderly's non-cooperation or lack of understanding. For example, we have a nursing assistants who patiently demonstrates over and over again when teaching the elderly how to take care of themselves until the elderly fully understand. Nursing assistants need to collaborate with other nursing assistants, doctors, and nurses to provide comprehensive care for the elderly. For example, we have a team of nursing assistants who meet weekly to discuss the patient's progress and coordinate care plans. Ability to communicate effectively with the elderly and their families to ensure that they understand the importance of pressure injury prevention. For example, one of our nursing assistants helps families understand how to care at home by making a simple pressure injury prevention manual. Careful inspection of the skin is the key to early detection of PI and their risks. Only through careful observation can nursing assistants detect subtle changes in the skin and take timely measures. Each patient's skin condition is unique, and careful inspection can help nursing assistants understand the individual differences of patients and provide personalized care plans. By observing the patient's behavior, expression, and speech, nursing assistants can detect possible psychological problems of the patient at an early stage, such as anxiety and depression, which may affect the patient's physical health and wound healing. Patients with a good psychological state are more likely to actively cooperate with nursing measures. Through observation and communication, nursing assistants can help patients maintain a positive attitude and improve nursing effectiveness.

**Interviewer:**

How do institutional culture and policies influence nursing assistants' motivation to perform PIPM?

**Interviewee:**

I think institutional culture and policies have a significant impact on nursing assistants' motivation to perform PIPM. A supportive institutional culture can encourage nursing assistants to actively participate in PIPM. When the institutional culture emphasizes teamwork, respect and recognition of individual contributions, nursing assistants are more likely to feel that their work is valued and thus more actively participate in pressure injury prevention. Institutions should set clear PIPM goals and expectations. When nursing assistants understand their work goals and these goals are aligned with the overall goals of the institution, they are more motivated to achieve them. Institutions should provide rewards and recognition for nursing assistants who perform well in PIPM. This can be bonuses, recognition, promotion opportunities or extra vacation time. This positive incentive can significantly improve nursing assistants' motivation. Adequate resources, such as high-quality nursing equipment and supplies, are key to implementing effective PIPM. The institution's investment in these resources demonstrates a commitment to the quality of care, which motivates nursing assistants to work harder. Educational training opportunities provided by the organization can enhance the professional knowledge and skills of nursing assistants, making them more capable of performing PIPM. Continuous education and professional development are seen as evidence of the organization's investment in employee growth and success. Our organization has implemented a PIPM award program to reward nursing assistants who have come up with innovative ideas or achieved significant results in pressure injury prevention. This not only increases their enthusiasm, but also promotes the importance of PIPM for the entire team.

**Interviewer:**

What motives would further empower nursing assistants to perform PIPM effectively?

Interviewee: I think the following motivations can further enhance the effectiveness of nursing assistants in implementing pressure injury prevention and management: Personal accomplishment, when nursing assistants see that their efforts have prevented the occurrence of pressure injury or improved the treatment outcomes of patients, they will feel a great sense of accomplishment. This sense of accomplishment can be a strong motivation for them to continue their efforts. Nursing assistants are often eager to improve their professional skills and knowledge. Providing professional development opportunities, such as participating in advanced training courses or obtaining professional certifications, can motivate them to focus more on PIPM. Being recognized and supported in the team can enhance nursing assistants' sense of belonging. When their contributions are seen and appreciated by the team, they are more likely to continue to actively participate in PIPM. Positive feedback from management or colleagues can greatly enhance nursing assistants' motivation. Rewards can be formal (such as bonuses, promotions) or informal (such as praise, certificates), which can motivate them to work harder. Providing regular training on the latest PIPM knowledge and skills can help nursing assistants stay at the forefront of their professional knowledge, thereby improving their ability and confidence to implement PIPM. Improving nursing assistants' job satisfaction can enhance their motivation. This can be achieved by improving the work environment, providing adequate rest time, and ensuring work-life balance.

**Interviewer:**

Well, OK, then please tell us what you think about the training of nursing assistants in nursing homes for pressure injury?

**Interviewee:**

Well, training is definitely necessary. Because there are a lot of training contents in the prevention and management of pressure injury. And it is very necessary, because if you do not do well in any direction, it may lead to this. In terms of content, I think the first is the ability to identify risks to identify what kind of patients have this risk, and then the second is how to prevent the occurrence of pressure injury. The third is how to deal with it. For example, if the problem is more serious, you may need to go to the hospital for treatment. If it is relatively mild, you may be able to promote the healing of the wound by yourself. This is also a prevention, a treatment, and identification, that is, what kind of tools to use, to prevent recurrence, and also immediate prevention.

**Interviewer:**

Okay, please talk about the needs and suggestions for pressure injury training?

**Interviewee:**

In terms of training content needs, I think prevention is the most important for nursing assistants, that is, it must be discovered in time. For some patients with weak self-ability who are bedridden for a long time. When he first entered the nursing home, he should have paid more attention to them and given them some extra attention, because they have the highest risk probability. Among these aspects, prevention is a key point of the nursing assistants's work. Because he does not have certain professional knowledge, if it is serious, he must increase the frequency. What kind of mattress should he choose? How should he position himself? What tools should I choose to prevent this? For example, my bed is relatively hard, and the frequency of lying on the bed is 1-2 hours, but his skin is still damaged. Well, can the mattress be adjusted? Or some people have scoliosis, and some people use the triangular cushion, but it is not easy to use. If the patient is a little restless, he will slide off immediately, and it will still not work after sliding off. Is there anything else to choose? It can make him less likely to slide. Well, some people have incorrect positioning equipment, and some people have edema, so they choose the foot ring to raise the bamboo pole. Well, but in clinical practice, we see many people who use their feet to crush the Achilles tendon first, which is an incorrect choice of equipment.

In terms of training methods, I think offline training is definitely necessary, so is online training necessary? It depends on the culture of the nursing home nursing assistants. If he doesn't know how to use a computer and no one helps him to use a computer, it may be difficult to do it online. If the nursing assistants has a better cultural level and can choose online training, it is also possible. If he has some theoretical knowledge in some aspects, he can choose online training. Well, offline training is definitely necessary. For example, how can he turn over without effort and can he turn over in place, right? These must be handled offline. And the patient has some questions like how to take nutrition, these things can be chosen online, right? It is better to be more theoretical and practical. Tell him how many meals a day he eats, what he mainly eats, how to operate, and you can talk to him. They will know after talking. For example, the theoretical teaching must include some necessary theories, and then the skill operation, including turning over and how to place the body position. This needs to be carried out in practice. You can basically explain the case analysis through PPT, and video teaching is also OK. Well, we will also have some videos to teach the nursing assistants how to turn over. Actual observation is also OK. Then we have some nursing homes, and we can invite some professional personnel to the nursing homes to see if the equipment you use is appropriate.

In terms of training resources, we have pressure injury identification here, what does the 1st, 2nd, 3rd and 4th stage look like, there are pictures, you can see, and there are also simulation equipment. There are quite a lot of peer learning, and generally family members are more interested. Well, it can be the same for the patient, right? They like to see what other patients are like, and group discussions are also OK. They will communicate with each other, all kinds of cases. Then this can also enrich their practice. The workshop is to teach these people how to put on and use some of the dressings for pressure injury prevention, right? So we can teach them through this workshop. Because there are so many types of dressings for pressure injury prevention, some people have never seen them and don't know how to use them. So I will use a workshop to gather all the materials brought by various hospitals, and explain to these nursing assistants the various dressings, their characteristics, and what kind of wounds they are suitable for. Well, it may be better this way, and it is better to use this in the workshop.

Our hospital has a guidebook for pressure injury prevention. We have made a poster for the nursing home, which can be placed in their nursing home during specific publicity, such as pressure injury prevention day. If they want to promote their excellent nursing quality, they can make such a poster. In fact, it is the same as our school, that is, let these nursing assistants draw a poster by themselves, and then make some edited videos, and then make a promotional material. Their nursing assistants are equivalent to nursing homes, so I can get my own brand, right? Hey, I can accept patients with pressure injury here. The pressure injury care here is better than other nursing homes. If I have professional knowledge, I will have this brand, and I will attract these patients, especially the elderly, and those with better financial ability will choose this nursing home, right? People will definitely be willing to choose your publicity. Well, this is better, I will go there to have a guarantee. Then this can also improve the overall level of this industry, and it may be more competitive in the future. The level of the nursing assistants will gradually improve. Well, the level of the nursing assistants will definitely improve slowly in the future.

**Interviewer:**

Do you have any suggestions on the frequency and time of training?

**Interviewee:**

I think the level of this training, first of all, if you are a new nursing assistants, this new nursing assistants must have a centralized exam before joining the job. I think it can be divided into different levels first, for example, like a cram school, first according to your different levels, then select you first, and then conduct a deep teaching. Hey, don’t they have that kind of confinement nanny? There should be gold medals. I have gold medal nursing assistants, etc. Then what kind of serious illness can I take care of? Then I or the next level will take care of the lighter and easier ones. The examination can be formulated according to the patient, that is, the situation of the nursing assistants. Then, it may be that the different levels have different levels of mastery. Then, in this way, he can still have the same experience as after the clinic. This is related to his experience, his habit of active learning, and his sense of responsibility. I think it can be used as a reference. You can take care of so many patients with pressure injury and do a good job. Well, then I can give you a higher level, right?

**Interviewer:**

What is the training effect evaluation method?

**Interviewee**

For the examination, it is recommended to have a one-person operation assessment and a theoretical assessment, or it is better to have a knowledge and skills questionnaire.

**Interviewer:**

Okay, thank you very much for your very detailed answer to the above questions. In addition to the above questions, do you have anything else to add?

**Interviewee:**

No

**Interviewer:**

Okay, thank you very much for your valuable opinions and participation in this interview. Your opinions will help improve the management of pressure injuries in our nursing homes. If you have any additional information, please feel free to contact me. Thank you very much.
