# Supplementary material for: Exploring nursing assistants’ competencies in pressure injury prevention and management in nursing homes: a qualitative study using the iceberg model
Source: BMC Nurs. 2025 Mar 27;24:333. doi: 10.1186/s12912-025-02911-6 (PMC11948734; doi:10.1186/s12912-025-02911-6)
Supplement: Supplementary file 2 — Supplementary Material 2 [file 12912_2025_2911_MOESM2_ESM.docx]

**Supplementary material:** Main categories, subcategories, codes, and meaning units forming nursing assistants’ PIPM competencies

| **Main Category** | **Subcategory** | **Code** | **Meaning Units** |
| --- | --- | --- | --- |
| Theoretical knowledge | Basic theoretical knowledge | Professional ethics and code of conduct for nursing assistants | “As nursing assistants, first of all, they need to know what the professional norms of nursing assistants are, basic professional ethics knowledge, professional qualities, and professional skills. In particular, they need to understand the requirements of nursing homes for PI quality management and the relevant reporting process”. (In-depth interview with nursing home administrator).  “The professional ethics and code of conduct of nursing assistants in nursing homes are important guarantees for ensuring service quality and the welfare of the elderly. According to the <Regulations on the Management of Nursing Homes>, nursing homes and their staff should protect the personal rights, property rights, and other legal rights of the elderly in accordance with the law”. (In-depth interview with local government’s policy-maker).  “They should continue to learn and improve their professional knowledge and skills, maintain sensitivity to the field of nursing care, and understand new concepts, new methods, and industry development trends”. (In-depth interview with nursing home administrator). |
|  |  | Legal and ethical issues related to elderly care and PI | “Basic knowledge of laws and regulations, especially those related to nursing homes, such as the <Regulations on the Administration of Nursing Homes>, Service Standards for Prevention of Pressure Injuries in Nursing Homes". and the <Law on the Protection of the Rights and Interests of the Elderly>”. (In-depth interview with nursing home administrator).  “In terms of professional ethics, nursing home nursing assistants should abide by the following standards, including respecting and protecting the rights and interests of the elderly, not discriminating against or insulting the elderly, following the autonomous wishes of the elderly, and protecting the privacy of the elderly”. (In-depth interview from nursing home administrator). |
|  |  | Definition and epidemiological characteristics of PI | “Nursing assistants need to know what PI is and why it is an important indicator of quality control in nursing home management. Therefore, they need to understand the current epidemiological characteristics of PI, the prevalence of PI in hospitals, communities, and nursing homes, and the prevalence of PI in the elderly population. They need to know this data to better implement PIPM”. (In-depth interview with nurses). |
|  |  | The hazards and dangers of PI | “Nursing assistants should know that pressure injuries not only have a serious impact on the patient's physical health, but also increase the difficulty and complexity of nursing work. PI will cause pain and discomfort to patients, increase the risk of infection, increase economic burden, and cause mental health problems”. (In-depth interview with nurses). |
|  |  | Mechanisms and risk factors of PI | “It is crucial for nursing assistants to understand the pathogenesis and risk factors of PI, as this helps them perform PIPM more effectively. Understanding the pathogenesis can help nursing assistants take preventive measures to reduce the occurrence of PI. Identifying risk factors enables nursing assistants to conduct risk assessments on patients and determine which patients are more likely to develop pressure injuries”. (In-depth interviews with nurses)  “Based on the patient's specific circumstances and risk factors, nursing assistants can develop personalized care plans. Nursing assistants can convey this basic knowledge to patients and families to help them understand the importance of preventing PI”. (In-depth interviews with nurses).  “Understanding the pathogenesis can help nursing assistants identify the signs of PI early and intervene in time. Timely prevention and treatment can improve the patient's overall health and prognosis”. (In-depth interviews with nurses). |
|  |  | Staging and clinical manifestations of PI | “Understanding the different stages of PI can help accurately assess the patient's wound condition. The staging of PI is crucial for determining treatment options (such as dressing selection, turning frequency, whether surgery is needed, etc.). Accurate staging helps record the progression of the wound and monitor the effectiveness of treatment”. (In-depth interviews with nurses).  “We can explain the severity and treatment process of PI to patients and families, helping them understand the importance of nursing measures”. (Focus group from nursing assistants). |
|  |  | High-risk populations and body sites for PI | “The high-risk groups for PI we usually encounter at work are mainly patients with paralysis, hemiplegia, paraplegia, or cerebral palsy. Due to limited mobility, they are prone to PI on bony protrusions. In addition, comatose, incontinent, and malnourished elderly people often develop PI, so this group of people should be paid special attention to as nursing assistants”. (Focus group from nursing assistants).  “When doing skin examinations, nursing assistants should know which parts to focus on, such as the occipital tuberosity, scapula, elbows, sacrum, and heels in the supine position, the ears, shoulders, elbows, ribs, hips in the lateral position, the inner and outer ankles of the knees, and the ears, female breasts, male genitals, knees, toes, etc. in the prone position”. (In-depth interview from nurses). |
|  | Professional theoretical knowledge | Causes and characteristics of PI among the elderly | “It is very important for nursing assistants to understand the causes and characteristics of PI in the elderly. This can help them realize that the elderly are a high-risk group for PI and improve their awareness of prevention. Timely identification and treatment of PI can reduce the occurrence of complications such as infection and reduce the health risks of patients”. (In-depth interview from nurses). |
|  |  | Methods for risk assessment of PI | “Nursing assistants need to learn how to assess the risk of PI. Many clinical guidelines recommend the use of risk assessment tools. Nursing assistants’ understanding of these tools will help them follow these guidelines. Risk assessment provides a basis for recording and analyzing changes in patient conditions and helps monitor the effectiveness of care. Accurate risk assessment and corresponding nursing measures can reduce medical disputes caused by PI”. (In-depth interview from nurses). |
|  |  | Methods for differentiating PI from other common skin problems | “Sometimes it is difficult for us to identify whether it is a PI skin problem during nursing care, and we are not sure to make a correct judgment. We don’t know whether it is a PI, incontinence-related dermatitis or other skin problems. We can’t differentiate them correctly. Therefore, we often cannot implement preventive measures in time”. (Focus group from nursing assistants). |
|  |  | Nutrition care | “Nutrition is very important for the elderly, especially for wound healing. Proper nutrition can enhance the resistance of skin and tissues and reduce the risk of PI. Knowledge of nutrition enables nurses to provide personalized dietary advice based on the patient's specific situation. Nurses can teach patients and families about the importance of nutritional care and help them understand how to improve their health through diet”. (In-depth interview from nurses). |
|  |  | Skin care | “Nursing assistants are the first line personnel who have direct contact with the elderly. Mastering the correct skin care techniques can help them deal with patients' skin problems in a timely manner, ensure their comfort and health, and reduce the risk of PI”. (In-depth interview with nurses). |
|  |  | Treatment and management of PI | “Understanding the treatment of PI can help nursing assistants provide professional care. Treating PI requires considering the patient's overall health. Nursing assistants should understand the principles of treatment such as cleaning the wound, removing necrotic tissue, using appropriate dressings, and possible medications”. (In-depth interview with nurses). |
|  |  | Types and functions of pressure relief equipment | “Nursing assistants need to know that there are many types of mattresses and cushions that are designed to distribute pressure and reduce the risk of PI for patients who spend a lot of time in bed or in a wheelchair. Local pressure relief devices such as wedges, pillows, and washers can be used to relieve pressure in specific areas, such as the heels, elbows, or ankles. For patients who need to sit for a long time, pressure relief chairs can provide additional support and pressure distribution. Wheelchair cushions are designed to fit the shape of the wheelchair and provide pressure relief for the sitting bones and other pressure areas. Knowledge of these pressure relief devices is important to know how to choose and use them to reduce the risk of PI”. (In-depth interview with nurses). |
|  |  | Types and characteristics of dressings | “We need to understand various wound dressings, including but not limited to film dressings, hydrogels, foam dressings, etc. Each dressing has its specific purpose. We need to choose the most suitable dressing according to the type, size, depth and amount of exudate of the wound. The choice of dressing should also take into account the comfort of the patient. Some dressings are not easy to damage the skin tissue when changing, which reduces the pain of the patient. On the premise of ensuring the effect, we also need to consider the cost-effectiveness of the dressing and choose cost-effective products for patients. However, since there are many types of wound dressings on the market, we sometimes cannot distinguish their functions, so the knowledge in this area is still lacking”. (Focus group with nursing assistants). |
|  |  | Psychological care for patients with PI | “We need to realize that PI are not only a physical condition, but also have an impact on the elderly's psychological state. Elderly people with PI may feel depressed, anxious, or lose self-esteem. We need to learn to provide emotional support and encouragement. By building a trusting relationship, we can help the elderly feel safer and more willing to accept care and treatment”. (Focus group with nursing assistants).  “Nursing assistants need to be able to identify whether the elderly have signs of psychological problems such as depression and anxiety and report them to medical staff in a timely manner. During the care process, they need to always respect the dignity and autonomy of the elderly and make them feel respected and valued”. (In-depth interview with nurses). |
| Comprehensive skills | Practical Skills | Tuning over and repositioning skills | “Turning and changing body positions are basic techniques for preventing PI. Correct turning techniques can help promote blood circulation in patients and reduce poor blood circulation caused by staying in the same position for a long time”. (In-depth interview with nurses).  “While helping patients turn over, we can check the skin condition and find redness, swelling or other abnormalities in time, which helps to identify the risk of PI early. Regular turning can reduce the discomfort caused by lying in the same position for a long time”. (Focus group with nursing assistants). |
|  |  | Position transfer techniques | “We must master the correct transfer techniques to ensure that we transfer patients safely and efficiently, and avoid falls or other accidental injuries. If the transfer is not performed properly, it may cause pain or discomfort to the patient. We need to ensure that the transfer process is as smooth and comfortable as possible. We often need to use various assistive devices, such as wheelchairs, walkers, lifts, or pulley sheets, to help transfer patients”. (Focus group with nursing assistants). |
|  |  | Wound dressing change technique | “Wound dressing is a basic nursing skill that we nursing assistants need to master in order to provide timely and appropriate wound care for our patients. Through proper cleaning and dressing changes, we can help keep wounds clean and create the best environment for healing”. (Focus group with nursing assistants).  “It is essential for nursing assistants to master basic wound dressing techniques to provide high-quality care, which helps ensure that patients' wounds are properly managed. Nursing assistants are an important part of our medical team. Their mastery of this technique allows them to collaborate more effectively with doctors and nurses to promote patient recovery”. (In-depth interview with nurses). |
|  |  | Use of walking aids | “Nursing assistants need to master the correct use of walkers to ensure that patients can move and transfer safely. Mastering the use of walkers can help reduce the risk of patients falling, especially when they first try to stand or walk”. (In-depth interview with nurses). |
|  |  | Selection and usage methods of dressings | “Choosing the right dressing is crucial to promoting wound healing. Different wound types and stages require different dressings to maintain a suitable healing environment. When using dressings, some precautions must be understood, such as following the principle of aseptic operation to prevent wound contamination. These methods need to be mastered by nursing assistants”. (In-depth interview with nurses). |
|  | Communication skills | Communication with nurses | “The prevention and management of PI requires the close cooperation of a multidisciplinary team. Effective communication between nursing assistants and nurses is the basis of teamwork. Nursing assistants are often the first to discover problems in daily patient care. They need to communicate with nurses in a timely manner about any changes in the patient's skin condition, including risk factors for PI”. (In-depth interview with nurses).  “We need to implement PIPM according to the PI prevention and management plan developed by nurses. Good communication ensures the consistency and coherence of the care plan”. (Focus group with nursing assistants). |
|  |  | Communication with the elderly and families | “Sometimes we encounter challenges in our work, such as it is difficult to communicate with some patients and their families, and we are not very good at using communication methods that they can accept to resolve conflicts encountered when implementing PIPM. For example, some patients are not very cooperative in turning over, changing clothes, wiping their bodies to keep their skin clean, etc. These will affect the occurrence of PI, we want to learn how to communicate effectively.” (Focus group with nursing assistants). |
|  | Collaboration skills | Collaboration with nurses | “The prevention and management of PI requires close collaboration between nurses and nursing assistants. Our professional capabilities and experience can complement each other and work together to provide comprehensive care for patients. The care plan developed by the nurses needs to be accurately implemented by the nursing assistants. This includes turning plans, skin care, nutritional support, and other PI prevention measures”. (In-depth interview from nurses). |
|  |  | Collaboration with other nursing assistants | “Collaboration ability is required. PI prevention and management is a team effort. We need to support each other, share responsibilities, and ensure that every nursing measure is properly implemented. Each nursing assistant has his or her own unique experience and skills. By working together, we can share each other's experiences and best practices and improve the level of care together. In daily work, timely information exchange is essential for patient care. We need to share the patient's condition changes and nursing needs with colleagues.” (Focus group with nursing assistants). |
|  | Observation skills | Observing the skin condition | “Nursing assistants’ ability to observe skin conditions is crucial for early identification of PI. Early detection of skin changes allows for preventive measures to be taken in advance. By observing skin color, temperature, moisture, and integrity, nurses can help us make accurate PI risk assessments”. (In-depth interview with nurses). |
|  |  | Observing the elderly’s physical and mental condition | “By observing the patient's behavior, expression, and speech, nursing assistants can detect possible psychological problems of the patient at an early stage, such as anxiety and depression, which may affect the patient's physical health and wound healing. Patients with a good psychological state are more likely to actively cooperate with nursing measures. Through observation and communication, nursing assistants can help patients maintain a positive attitude and improve nursing effectiveness”. (In-depth interview with nurses). |
| Self-concept | Professional identity | Enthusiasm for the elderly care profession | “Identity acknowledge and passion for the elderly care profession is the driving force for nursing assistants to provide quality care services. Passion for this job means that nursing assistants will be more dedicated and attentive to the care of elderly patients. Passion can motivate them to continue learning and improving, which is crucial for their professional development in PI prevention and management”. (In-depth interview with nurses). |
|  |  | Sense of belonging | “A sense of belonging makes nursing assistants feel that they are an indispensable part of the elderly care team. This sense of identity can inspire their pride and enthusiasm for their work. Nursing assistants who feel a sense of belonging to their profession are more likely to provide emotional support, which is very important for the mental health and recovery of PI of elderly patients.” (In-depth interview with nurses). |
|  |  | Commitment to work | “Dedicated nursing assistants always put the needs of patients first, which helps ensure the effective implementation of PI prevention and management measures”. (In-depth interview from nurses).  “We often take care of patients without fear of hardship, taking care of more than 5 elderly people every day, working more than 8 hours a day. This is our job. Since we have chosen this job of caring for the elderly, we must be prepared and committed to devote our time, energy and physical strength”. (Focus group from nursing assistants). |
|  | Ethical awareness | Comply with the laws and regulations | “Many ethical laws and regulations set standards and requirements for nursing services. Nursing assistants need to comply with these standards when implementing PI prevention and management to ensure the quality and safety of care. Laws and regulations protect the rights of patients, including the right to informed consent, privacy, and the right to receive appropriate care. Nursing assistants need to be aware of these rights and respect them in their daily work”. (In-depth interview from nurses). |
|  |  | Respect the elderly’s esteem and privacy | “Respecting the dignity and privacy of older people helps build trusting relationships between patients and us, which is the foundation of effective care. When patients feel respected, we are more likely to actively cooperate with care interventions, including the prevention and management of PI”. (Focus group from nursing assistants). |
|  |  | Treat the elderly equally | “We need to recognize that each elderly person is a unique individual whose needs and preferences should be respected and considered. When elderly people feel they are treated equally, they are more likely to actively participate in our care plans, including PI prevention measures”. (Focus group from nursing assistants). |
|  | Sense of responsibility | Pay attention to PI prevention | “PI are a serious complication that can increase patient suffering and healthcare costs. Nursing assistants need to fully recognize the seriousness of PI and regard them as a priority in their nursing work. Nursing assistants who pay attention to PI are more likely to follow clinical guidelines and best practices and ensure that all nursing activities are based on scientific evidence”. (In-depth interview with nurses). |
|  |  | Emphasis on care quality | “The quality of care is a key indicator of our service level. Nursing assistants must pay attention to the quality of care and ensure that every step meets the highest standards of care. Paying attention to the quality of care will help identify and implement effective PI prevention measures and reduce the occurrence of complications”. (In-depth interview with nurses). |
|  |  | Don't shirk responsibility | “When faced with challenges in PI prevention and management, nursing assistants need to proactively solve problems rather than avoid responsibility. This attitude helps resolve problems quickly and effectively. Nursing assistants who do not shirk responsibility are more likely to learn from their experiences, continue to grow, and improve their professional abilities”. (In-depth interview with nurses). |
| Traits | Carefulness and love | Checking the elderly's skin carefully | “Careful inspection of the skin is the key to early detection of PI and their risks. Only through careful observation can nursing assistants detect subtle changes in the skin and take timely measures. Each patient's skin condition is unique, and careful inspection can help nursing assistants understand the individual differences of patients and provide personalized care plans”. (In-depth interview with nurses). |
|  |  | Encourage the elderly to be self-reliant within their capabilities | “Self-reliance support can enhance the elderly's sense of self-efficacy, making them believe that they can control their own lives and health management. Encouraging self-reliance support for the elderly can help improve the efficiency of nursing work because the elderly can take care of themselves within their ability”. (In-depth interview with nurses). |
|  |  | Do not blame the elderly | “Elderly patients may not be able to fully control their behavior due to physical or cognitive limitations. We should treat them with respect and understanding, rather than blaming them. When we avoid blaming and adopt an encouraging and supportive approach, patients are more willing to open up and share their feelings and needs”. (Focus group with nursing assistants). |
|  |  | Actively focusing on the needs of the elderly | “By actively paying attention to the needs of the elderly, nursing assistants can identify potential PI risks early and take timely measures. When the needs of the elderly are paid attention to, they are more likely to actively participate in their own care process, including the prevention and management of PI”. (In-depth interview with nurses). |
|  | Empathy and patience | Ability to stand in elderly’ points | “Thinking from the perspective of the elderly allows me to better understand their feelings and needs, which is a manifestation of empathy. If I can understand the perspective of the elderly, communication will become smoother, and I can convey information more accurately and explain the importance of PI prevention and management”. (Focus group with nursing assistants). |
|  |  | Be patient with the elderly’s slow reaction | “Elderly people may become slow to respond for various reasons. We need to respect these individual differences and treat them with patience. Patience can help me build a trusting relationship with elderly patients and make them feel at ease and cared for”. (Focus group with nursing assistants). |
| Motives | Professional development opportunities | Competency enhancement | “Nursing can be challenging at times. However, when I thought these challenges could enhance my competency, I would have the motivation to conduct the nursing service. This belief has enabled me to maintain a positive attitude and not give up even when things get tough”. (Focus group with nursing assistants). |
|  |  | Job promotion | “I want to make progress in my career, which motivates me to continuously improve my work standards and ensure that every nursing care is of high standard. I hope to be a leader in the team, contribute to the team through my efforts and expertise, and jointly improve the level of nursing services”. (Focus group with nursing assistants). |
|  | Supportive institutional policies | Assessment and evaluation mechanism | “The assessment mechanism is the key to ensure that nursing assistants provide high-quality nursing services. Through regular and systematic assessment, we can ensure that the prevention and management measures for PI are effectively implemented. The assessment results help us identify the training needs of nursing assistants in the prevention and management of PI, to motivate them to get targeted education and training”. (In-depth interview with local government’s policy-maker). |
|  |  | Continuing education credits | “Continuing education credits can be used as a mechanism to motivate nursing assistants to actively participate in training and learning, thereby increasing their work enthusiasm. Encouraging nursing assistants to obtain continuing education credits can help improve the professional level of the entire nursing team and promote the overall development of the team”. (In-depth interview with nursing homes’ administrators). |
|  |  | Reward mechanism | “The reward mechanism can significantly improve our work enthusiasm and encourage us to provide higher quality nursing services. Through rewards, teamwork spirit can be promoted and we can be encouraged to collaborate with each other to jointly improve the effectiveness of PI management”. (Focus group with nursing assistants). |
